# Nutritional Advice in Older Patients at Risk of Malnutrition during Treatment for Chemotherapy: A Two-Year Randomized Controlled Trial

**DOI:** 10.1371/journal.pone.0108687

**Published:** 2014-09-29

**Authors:** Isabelle Bourdel-Marchasson, Christelle Blanc-Bisson, Adélaïde Doussau, Christine Germain, Jean-Frédéric Blanc, Jérôme Dauba, Cyril Lahmar, Eric Terrebonne, Cédric Lecaille, Joël Ceccaldi, Laurent Cany, Sandrine Lavau-Denes, Nadine Houede, François Chomy, Jessica Durrieu, Pierre Soubeyran, Pierre Senesse, Geneviève Chene, Mariane Fonck

**Affiliations:** 1 CHU Bordeaux, Clinical Gerontology, Bordeaux, France; 2 CNRS, RMSB, UMR 5536, Bordeaux, France; 3 Université Bordeaux, RMSB, UMR 5536, Bordeaux, France; 4 CHU Bordeaux, Unité d'épidémiologie clinique, Bordeaux, France; 5 INSERM, CIC-EC7, Bordeaux, France; 6 CHU Bordeaux, Hépatologie, gastroentérologie and oncologie digestive, Bordeaux, France; 7 Université Bordeaux, Bordeaux, France; 8 Centre Hospitalier Layné-Mont de Marsan, Service Oncologie, Mont de Marsan, France; 9 CHU Bordeaux, Service Hépato-gastro et oncologie digestive, Bordeaux, France; 10 Polyclinique Bordeaux Nord, Service hépato gastroentérologie, Bordeaux, France; 11 Centre Hospitalier de Libourne, Libourne, France; 12 Clinique Francheville, Périgueux, France; 13 CHU Limoges, Unité de recherche clinique en oncologie médicale, Limoges, France; 14 Institut Bergonié, Departement d'oncologie médicale, Bordeaux, France; 15 Universités Montpellier 1 et 3, Laboratoire Epsylon EA 4556, Montpellier, France; 16 CLCC du Val d'Aurelle, Unité de Nutrition clinique et de gastroentérologie, Montpellier, France; The Chinese University of Hong Kong, Hong Kong

## Abstract

**Objective:**

We tested the effect of dietary advice dedicated to increase intake in older patients at risk for malnutrition during chemotherapy, versus usual care, on one-year mortality.

**Method:**

We conducted a multicentre, open-label interventional, stratified (centre), parallel randomised controlled trial, with a 1∶1 ratio, with two-year follow-up. Patients were aged 70 years or older treated with chemotherapy for solid tumour and at risk of malnutrition (MNA, Mini Nutritional Assessment 17–23.5). Intervention consisted of diet counselling with the aim of achieving an energy intake of 30 kCal/kg body weight/d and 1.2 g protein/kg/d, by face-to-face discussion targeting the main nutritional symptoms, compared to usual care. Interviews were performed 6 times during the chemotherapy sessions for 3 to 6 months. The primary endpoint was 1-year mortality and secondary endpoints were 2-year mortality, toxicities and chemotherapy outcomes.

**Results:**

Between April 2007 and March 2010 we randomised 341 patients and 336 were analysed: mean (standard deviation) age of 78.0 y (4·9), 51.2% male, mean MNA 20.2 (2.1). Distribution of cancer types was similar in the two groups; the most frequent were colon (22.4%), lymphoma (14.9%), lung (10.4%), and pancreas (17.0%). Both groups increased their dietary intake, but to a larger extent with intervention (p<0.01). At the second visit, the energy target was achieved in 57 (40.4%) patients and the protein target in 66 (46.8%) with the intervention compared respectively to 13 (13.5%) and 20 (20.8%) in the controls. Death occurred during the first year in 143 patients (42.56%), without difference according to the intervention (p = 0.79). No difference in nutritional status changes was found. Response to chemotherapy was also similar between the groups.

**Conclusion:**

Early dietary counselling was efficient in increasing intake but had no beneficial effect on mortality or secondary outcomes. Cancer cachexia antianabolism may explain this lack of effect.

**Trial Registration:**

ClinicalTrials.gov NCT00459589

## Introduction

Weight loss in patients with cancer has long been linked to poor prognosis [1]. It is therefore recommended to assess nutritional status in patients undergoing nonsurgical oncologic treatment [2], [3]. Dietary counselling has been proposed in malnourished patients with gastrointestinal cancers during chemotherapy treatment [4].

A randomised controlled trial of a nutritional intervention was conducted in 358 adults with weight loss and treated with chemotherapy for metastatic or locally advanced digestive or non-small cell lung cancer [5]. The aim was to assess the effects of nutritional advice and the prescription of an oral nutritional supplement, either alone or combined for 6 weeks. None of the interventions produced any benefit on outcomes including one-year mortality and quality of life. The lack of effect of the interventions may be due to the fact that cancers were advanced in this study. Another similar trial of dietary support during 12 weeks of chemotherapy reported no benefit in 192 patients with non-small-cell lung and colon cancer [6]. A meta-analysis of nutritional intervention trials in cancer patients in very heterogeneous situations (nutrition, cancer, treatments) did not show any benefit of these interventions on mortality [7].

All these studies involved adult populations but without any specific analysis of older patients. Nevertheless, cancers are more and more frequent in older people and in 2005 it was estimated that 56% of new cancers occurred in people older than 65 y [8]. One-year mortality increased by two-fold in older patients with cancer undergoing chemotherapy who were malnourished or at risk of malnutrition according to the MNA© (Mini Nutritional Assessment) [9]. Very few patients were malnourished (3 out of 202) so the relationships mainly concerned those at risk. In a similar study including more malnourished patients (MNA<17, 13.8%), the relative risk (RR) of one-year mortality was higher (2.77) for those with an MNA©<24 [10]. This suggests an effect related to the severity of nutritional impairment according to the MNA©. This specific tool for older patients includes items directly associated to nutrition, such as anthropometric measures and nutritional intake, together with health-related quality of life measures such as comorbidities, mental health, autonomy and subjective health [11], was shown correlated with cancer cachexia features [12] and was one of the independent predictor for chemotherapy toxicity [13]. MNA is thus the consensual tool to assess malnutrition in older patients with cancer [3], [14]. In older patients, the increased risk of mortality associated with a lower MNA© score may be due to factors other than cancer. Thus, nutritional support targeted on gerontological assessment may better address the needs of older patients with cancer [15]. No clinical trial has yet tested the effect of nutritional intervention in older patients with cancer who were malnourished or at risk of malnutrition according to the MNA© scale.

In the present randomised clinical trial involving older patients at risk of malnutrition, we assessed the effect of dietary counselling implemented from the start of chemotherapy for solid tumours and lymphoma on one-year and subsequent mortality and on severe toxicities.

## Methods

The protocol for this trial and supporting CONSORT checklist are available as supporting information; see Checklist S1 and Protocol S1.

### Study design

This study was a multicenter superiority randomized controlled trial of patients with cancer receiving chemotherapy in 12 public and private settings in South-West France, comparing Usual Care to Usual Care + Nutritional Intervention in two parallel arms. Patients were enrolled between April 2007 and March 2010 and follow-up ended in April 2012. The randomization list was prepared and stored by the clinical trial unit biostatistician. Randomization was centralized by internet, with a 1∶1 ratio, stratified on recruitment centre. Informed written consent was retrieved from patients after eligibility criteria were checked. All assessments and dietary interviews were performed in the cancer treatment setting. The institutional Review Board of South-West France and Overseas French departments, France, approved the study protocol. The trial was recorded with ClinicalTrials.gov, number NCT00459589.

### Patient selection

Patients older than 70 y with lymphoma or carcinoma with an indication of chemotherapy and a Karnofsky index higher than 50% were screened for participation. Eligible carcinomas were from the colon, stomach, pancreas and biliary tract, ovary, prostate, bladder, and lung. Lymphoma types were any B cell lymphoma, any T lymphoma, low malignancy lymphomas such as follicular, lymphoplasmacytic, lymphocytic, mantle, MALT, and other marginal zone lymphoma. Patients with a carcinoma of unproven origin but compatible with any tumour in the abovementioned list could be included if a chemotherapy was planned. Patients could receive a first to third line of routine chemotherapy. Patients with cerebral metastasis were not eligible. Any inability to take part in follow-up according to the schedule of the study for geographical or treatment-related reasons was an exclusion criterion. The full MNA© was used to screen patients at risk of malnutrition. The full MNA© is an 18-item questionnaire including anthropometric, general, dietary and subjective assessments; the maximum score is 30 indicating the best nutritional status and a score below 17 indicating malnutrition [11]. To be included in the randomized trial, subjects had to be at risk of malnutrition with a full MNA© in the 17 to 23·5 point range.

### Intervention

The Usual Care group (UC) received the nutritional care routinely given in the cancer treatment settings and there were no restrictions for dietary advice, oral supplements or prescription of artificial nutrition. The Usual Care+Nutritional Intervention (UC+NI) group received usual care and nutritional intervention.

Nutritional intervention began the first day of chemotherapy. The study dietician, who did not belong to the staff of the cancer treatment setting, provided dietary advice with the aim of achieving an energy intake of 30 kCal/kg body weight/d and 1.2 g protein/kg/d [2]. Counselling was based on face-to face-interviewing and dietary advice cards, and involved caregivers or relatives if possible [16]. Actual dietary intake and gerontological assessment routinely applied [15] were taken into account to adapt the advice to each patient. The eight dietary cards addressed the issues of: 1-dietary balance, 2-loss of appetite and enrichment, 3- diarrhoea and constipation, 4-nausea and vomiting, 5-taste disturbances, 6-oral pain and feeling of dryness, 7- swallowing disorders, 8- diabetes. Dietary advice was complemented by prescription of an oral supplement if pertinent in order to increase intake. Six face-to-face visits were planned during chemotherapy sessions and contact was made by phone in the event of an interval between visits longer than two weeks due to the schedule of the chemotherapy. Intervention lasted 3 to 6 months according to the chemotherapy schedule of each patient. All study dieticians received training for the study, which was also given if a new dietician entered it. The duration of the intervention was 3 to 4 months according to the duration of chemotherapy. None of the staff in cancer treatment centres were aware of the content of the nutritional intervention.

### Dietary intake assessment

Patients in both groups were instructed to fill in a one-day dietary record of the day before the visit. The UC patients gave their record to the staff of the centre and the UC+IC gave theirs to the study dietician who needed it to adapt counselling. Any intake was taken into account, including oral nutritional supplements and artificial nutrition. Nutrisoft Billnut software (Tours, France) was used to calculate intake by the coordinating centre [17]. Percent of patients achieving the goal in energy or protein intake were shown.

### Outcomes

The main outcome was one-year mortality recorded in centres. Causes of death were recorded. General practitioners and patients' relatives were contacted if needed.

Chemotherapy management (dosage, changes and arrest), grade 3–4 toxicities including severe infections, weight changes, prescription of enteral or parenteral nutrition, and hospitalization for reasons other than chemotherapy were considered as secondary outcomes. These data were collected by investigators during the chemotherapy period. Two-year mortality was also assessed.

### Determination of sample size

A reduction of 10% in one-year mortality was considered as clinically significant, considering a mortality rate in the UC group of 50% [9]. On the basis of the O'Brien and Fleming rule [18], an alpha 5% risk and a 80% power, with an interim analysis, and loss of follow-up expected lower than 5%, 820 patients had to be enrolled. The interim analysis was planned when half of the participants were evaluated for the main outcome, but it was not performed because recruitment was lower than expected and the study was stopped before.

### Statistics

Analyses were performed on an intention-to-treat basis. Comparisons of dietary intake between groups were performed using a mixed linear model accounting for repeated measures. The Kaplan-Meier method was used to estimate survival probabilities and mortality was compared using the Wald Chi2 test in a Cox model adjusted on the recruiting centre of the participants. Proportions were used to describe qualitative variables of the secondary outcomes. Comparisons were made with the Chi2 test. For the outcomes that were recorded at several visits such as infections, the outcome was considered absent when the data was not available in the main analysis. In a robustness analysis, we used the missing = failure strategy. Quantitative variables were described with mean and standard deviation (SD) or Inter-Quartile Interval (IQR) and compared using Student's t test or the Wilcoxon test according to the distribution of the analysed variable.

## Results

### Patient flow

**Figure 1:**
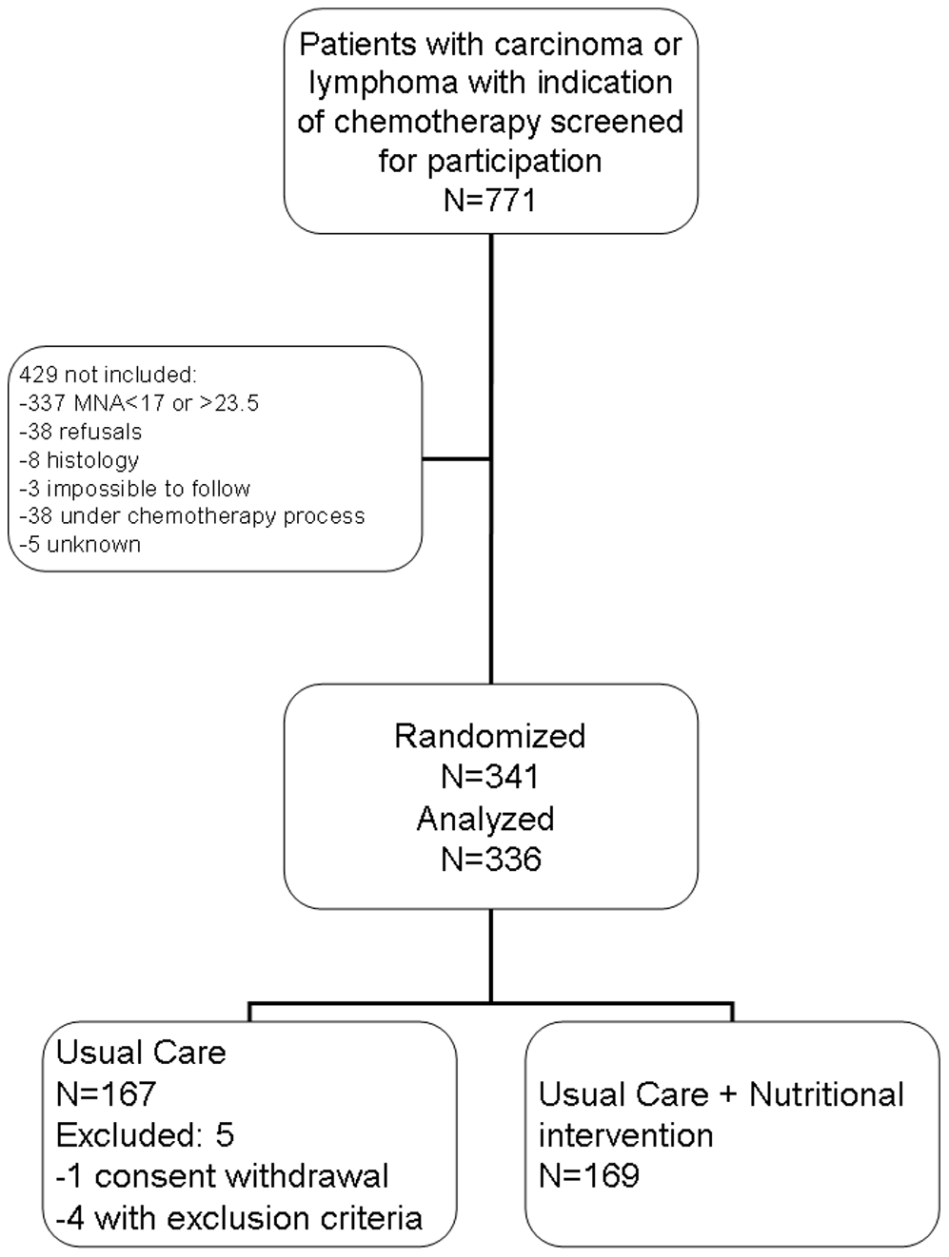
Flowchart of participant progression through a randomized controlled trial of nutritional intervention in older patients at risk of malnutrition.

Among 771 screened patients, 341 were randomized in the trial (**Figure** 1) and 336 were analyzed: mean (standard deviation, SD) age 78.0 y (SD 4.9), 51.2% male, mean MNA 20.2 (SD 2.1). Distribution of cancer types was similar in the two groups; the most frequent were colon (22.4%), lymphoma (14.9%), lung (10.4%) and pancreas (17.0%). Chemotherapy was first line in 83.9%. The trial was stopped before full recruitment at the end of the inclusion period due to an insufficient rate of inclusions. The slow recruitment was attributed to the existence of competitive trials for chemotherapy agents. The resulting power of the analysis was estimated 40% instead of 80% to detect a one-year mortality difference of 10% or was 80% to detect a 15% difference between the two groups. Among patients identified as eligible, reasons for non-inclusion were mainly chemotherapy treatments already started. Five more subjects were excluded from the analyzed sample due to one withdrawal of consent and 4 major deviations from the eligibility criteria in the UC group (head and neck tumour and chronic lymphatic leukaemia, or 5^th^ line of chemotherapy). Five patients with minor eligibility deviation (MNA<17 and one>23·5 and two with cardia cancer or endocrine carcinoma of the pancreas) were kept in the analysed sample after scientific committee approval. The resulting analyzed sample included 336 subjects. Groups were balanced for age, sex ratio, cancer characteristics, routine biochemical analyses and blood cell count (Table 1). Two participants from the UC+NI group refused to continue the intervention during the follow-up but were followed up for mortality. In the UC+NI group 877 dietician-visits were performed in relation to 990 planned visits so compliance with the intervention was estimated at 88.6%. There were also 450 phone contacts. None of the participants were lost to follow-up.

**Table 1:** Main baseline characteristics of participants in the trial.

	Usual Care	Usual Care + Nutritional Intervention
	N = 167	N = 169
Full MNA, mean (SD)	20.4 (2.1)	20.1 (2.0)
Age, years, mean (SD)	78.3 (4.7)	77.7 (5.2)
Gender, male % (n)	54.5 (91)	47.9 (81)
ECOG PS 0–1% (n)	71.6 (88)	78.4 (91)
Cancer % (n)		
Colon	19.2 (32)	25.6 (17)
Stomach	8.4 (14)	6.5 (11)
Pancreas and cholangiocarcinoma	17.4 (29)	19.7 (33)
Non-small cell lung	10.8 (18)	10.1 (17)
Prostate	5.4 (9)	2.4 (4)
Bladder	7.8 (13)	4.2 (7)
Ovary	7.8 (13)	7.1 (12)
Breast	7.2 (12)	9.5 (16)
Lymphoma	16.2 (27)	13.7 (23)
Metastasis (carcinoma), % (n)		
None	30.0 (42)	35·6 (52)
Mx (unknown at chemotherapy start)	8.6 (12)	8.2 (12)
IPI score 2–3 (lymphoma) % (n)	40·7 (11)	57.1 (12)
First line chemotherapy, % (n)	83.2 (139)	84.6 (143)
Weight change, % of usual body weight, mean (SD)	−8.6 (7.9)	−8.9 (6.6)
Lymphocytes/mm^3^, mean (SD)	1.6 (1.9)	1.6 (2.5)
Haemoglobin, g/100 ml, mean (SD)	12.0 (1.6)	11.8 (1.7)
Serum albumin, g/l, mean (SD)	36.8 (6.2)	36.9 (6.9)
C-reactive protein, mg/l, mean (SD)	34.7 (64.7)	34.1 (42.2)

ECOG PS: Eastern Cooperative Oncology Group performance status.

### Dietary intake

**Figure 2:**
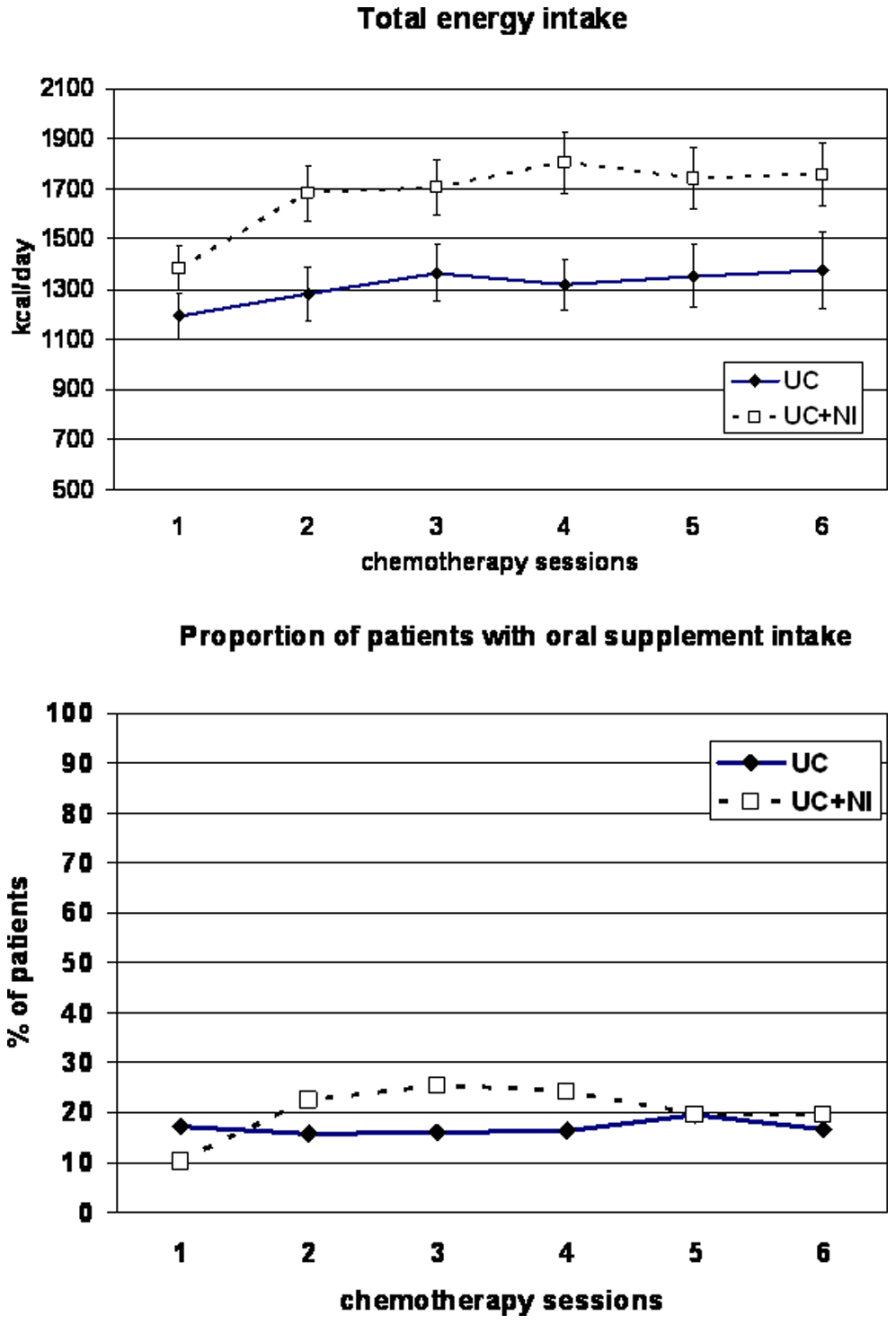
Dietary intake the day before each cycle during the chemotherapy period. Data are presented as mean and 95% CI, or proportion. Total dietary intake was analyzed with mixed models: increase of total intake at the second visit in both groups (UC+NI, P<0.0001; **UC, P = 0.02), with higher increased in UC+NI compared to UC, P<0.01.

The mixed model was applied in 1248 records, corresponding to 310 patients. At baseline, dietary intake was higher in the UC+NI group compared to the UC group (difference of 178 kcal/day, p<0.01). In both groups, dietary intake increased between visit 1 and visit 2 (UC+NI+328 kcal/day, p<0.0001; UC+132 kcal/day, p = 0.02) but the difference was higher in the UC+NI group than in the UC group (p<0.01) (**Figure** 2). At the visit 2, 57 (40.4%) patients in the UC+NI group compared to 13 (13.5%) in the UC group achieved the goal of 30 Kcal/kg/d or more and 66 (46.8%) in UC+NI group compared to 20 (20.8%) the goal of 1.2 g protein/kg/d.

In the UC group, 17.2% (23 patients) received a supplement at the first visit and the maximum rate during the follow-up was 19.6% (19 patients) (visit 5). In the UC+NI group, 10.3% (17 patients) received an oral nutritional supplement at the first visit and the maximum rate during the follow-up was 25.5% (42 patients) (visit 3).

### Outcomes

**Figure 3:**
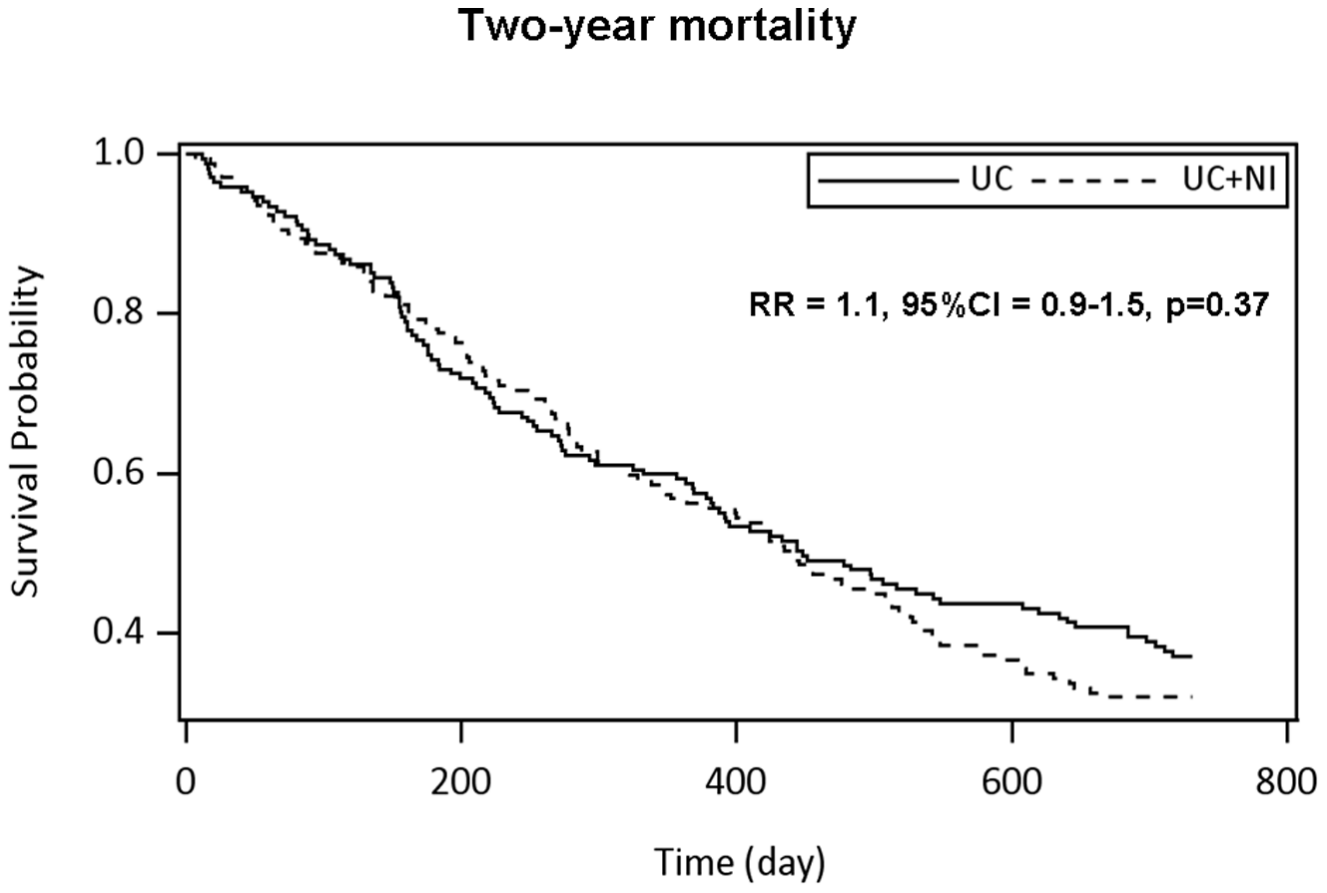
Two-year mortality according to groups UC and UC+NI. N = 336. Comparisons were performed with Cox model adjusted on recruiting centres.

One-year and two-year mortality were similar in both groups (**Figure** 3, respectively R = 1.1, 95%CI = 0.8–1.5, p = 0.74, and RR = 1.1, 95%CI = 0.9–1.5, p = 0.37). The main declared cause of death was cancer disease (Table 2). There was no difference in distribution of cause of death according to the groups. There was no lost of follow-up patient for the main outcome.

**Table 2:** Two-year mortality and other outcomes.

	Usual Care	Usual Care + Nutritional Intervention	P
	N = 167	N = 169	
One-year mortality, % (n)	41.3 (69)	43.8 (74)	0.74
Two-year mortality, % (n)	62.9 (105)	68.0 (115)	0.37
Mortality during chemotherapy, % (n)	3.7 (4)	6.4 (7)	
One-year mortality according to tumour origin, % of subject in tumour category, (n)			
Colon	18.8 (6)	30.2 (13)	
Stomach	50.0 (7)	54.5 (6)	
Pancreas and cholangiocarcinoma	62.1 (18)	66.7 (22)	
Non-small cell lung	77.8 (14)	76.5 (13)	
Prostate	22.2 (2)	75.0 (3)	
Bladder	53.8 (7)	57.1 (4)	
Ovary	15.4 (2)	8.3 (1)	
Breast	33.3 (4)	50.0 (8)	
Lymphoma	33.3 (9)	17.4 (4)	
Two-year mortality according to tumour origin, % of subject in tumour category, (n)			
Colon	40.6 (13)	48.8 (21)	
Stomach	78.6 (11)	81.8 (9)	
Pancreas and cholangiocarcinoma	75.9 (22)	87.9 (29)	
Non-small cell lung	94.4 (17)	94.1 (16)	
Prostate	88.9 (8)	100.0 (4)	
Bladder	84.6 (11)	71.4 (5)	
Ovary	15.4 (4)	41.7 (5)	
Breast	58.3 (7)	62.5 (10)	
Lymphoma	44.4 (12)	60.9 (14)	
Cause of one-year death, % (n)			
Cancer disease	89.9 (62)	86.5 (64)	
Chemotherapy toxicities	2.9 (2)	5.4 (4)	
Other	7.2 (5)	8.2 (6)	
Cause of two-year death, % (n)			
Cancer disease	93.3 (98)	90.4 (104)	
Chemotherapy toxicities	1.9 (2)	3.5 (4)	
Other	4.8 (5)	6.1 (7)	
Cause of two-year death, % (n)			
Cancer disease	93.3 (98)	90.4 (104)	
Chemotherapy toxicities	1.9 (2)	3.5 (4)	
Other	4.8 (5)	6.1 (7)	
Chemotherapy management % (n)			
At least one time chemotherapy not administrated,	65.0 (106)	64.8 (107)	0.97
At least one time change of protocol	62.0 (101)	64.2 (106)	0.67
Chemotherapy result at end of treatment, % (n)			
Full remission	11.1 (16)	7.0 (10)	0.39
Partial remission	23.6 (34)	30.8 (44)	
Stabilization	40.3 (58)	40.6 (58)	
Progression	25.0 (36)	21.7 (31)	
Weight change at end of chemotherapy, kg			
IQR	−4.0; 1	−5.0; 1	0.59
Proportion of patients with weight loss % (n)	57.7 (75)	56.9 (74)	
Change in serum albumin at end of treatment, g/l, IQR	−8.0; 1.4	−7.6; 3.0	0.59
Incident fall, pressure ulcer or fracture, % (n)	6.0 (9)	5.6 (8)	0.87
Infection grade 3–4, % (n)	10.4 (7)	4.2 (7)	0.03
Hospitalisation, % (n)	34.4 (56)	29.1 (48)	0.31
Enteral/parenteral nutrition, % (n)	9.2 (15)	5.5 (9)	0.19

Chemotherapy management and outcomes were similar in both groups (Table 2). There were more UC patients with grade 3–4 infections than UC+NI ones (Table 2, p = 0.03). However, a robustness analysis was performed due to the existence of missing data and did not confirm the difference in the incidence of severe infections. The rate of weight change and other secondary outcomes were similar in both groups.

## Discussion

This large randomised controlled trial investigated the effect of nutritional support in older patients treated by chemotherapy for cancer. Despite an increase in dietary intake that was higher in patients with dietary counselling, no improvement was noted in one- and two-year mortality in older subjects at risk of malnutrition and undergoing chemotherapy.

However, a lower rate of serious infections during chemotherapy was observed. This result should be interpreted with caution as it was not confirmed by the robustness analysis. A decreased rate of severe infections may have favourably impacted the prognosis but this event was relatively rare. The potential beneficial effect of nutritional intervention on this outcome was not sufficient to modify the survival of the group.

The trial was stopped before completion of the planned inclusions. However, it is unlikely that the lack of an observed effect of intervention is due to lack of power. In the whole sample, a trend of an increase in two-year mortality in the intervention group was seen. However, even if we had reached the planned sample size, such mortality rates in both arms would not have provided a significant difference in survival. This absence of effect on mortality and other outcomes was not due to unbalanced characteristics of patients between groups according to cancer disease, chemotherapy or nutritional baseline assessment. Furthermore, within each tumour type, one and two-year mortality rates were similar. Any non-drug intervention involving a person such as a dietician cannot be blind and patients and practitioners were aware of the allocation. Despite these limitations, the main result is reliable, all the more in that mortality is a very robust outcome and there were no patient lost to follow-up.

In adult patients with gastro-intestinal cancer, the increased mortality risk in patients with weight loss prior to chemotherapy has been attributed to decreased chemotherapy doses and increased toxicity rates [19]. The present findings do not support this hypothesis since we found no change in chemotherapy outcomes with nutritional support. However, we did not include malnourished patients (MNA©<17) or those with a very poor prognosis according to their Karnosky index in order to better focus on nutritional risk. Thus, we cannot draw any firm conclusions about the effect of malnutrition on chemotherapy outcomes.

In patients with cancer treated with chemotherapy, nutritional interventional trials are scarce and none have been devoted to older patients. A retrospective study showed that patients with colorectal cancers who benefited from dietary counselling improved their nutritional status and had better survival [20]. However, randomized controlled trials did not show in adult patients with cancer an effect of nutritional interventions on mortality [5], [7].

The absence of difference in survival between the intervention groups in the present study might be explained by two main hypotheses. First, the intervention might not sufficiently increase dietary intakes; second, the nutritional intervention might not be efficient to impact mortality in these patients.

In the present study, dietary counselling resulted in a positive effect on dietary intake in both groups after the start of chemotherapy. However, the targets of 30 kcal/kg/d or 1.2 g protein/d were not achieved in more than half of the patients, questioning the efficacy of dietary counselling in patients with cancer. On the other side, the patients in the UC group may have received more attention regarding their dietary intake than usual. This could have been a source of contamination bias, resulting in a lower difference in intakes between groups. The improvement in intake in both groups at cycle 2 might also be due to an improvement in health status owing to the global care given to these patients. The assessment of intake was different between the groups: in UC patients it was a self-report questionnaire with no help from a dietician; in UC+NI ones, the study dietician commented and improved the quality of the record with the patient during the interview. However, the baseline difference could be interpreted as a proxy of the measurement bias due to the assessment procedure, and the linear mixed model showed that, despite the baseline difference, the increase in intake was significantly higher in the intervention group between cycle 1 and 2. The other possible limitation regarding dietary intake assessment was the schedule of the records. We assessed the period distant from the chemotherapy session, so we probably assessed the highest intake during the period between two cycles. We made no assessment of the effects of chemotherapy on appetite just after the treatment. However, chemotherapy management was similar in both groups and the effects on appetite were likely the same. Two randomized studies previously showed an increase of actual dietary intake with dietary counselling in patients with solid tumours [21],[22].

Despite the higher dietary intake found in the intervention group, there were no differences in weight change between the groups. This finding is similar to the conclusion of other randomised control trials performed in adults [5],[22] in which no weight change difference was seen between the groups. The sample was not large enough to explore subgroups responses to intervention on intention to treat basis, all the more that they had not been planned in the trial design. It was thus not possible to identify if any, a sub-group of patients on the basis of their baseline characteristics, able to take advantage on the intervention, for example those who did or not lose weight prior to chemotherapy. In the already described RCT performed in adults selected on the basis of weight loss, irrespective of the intervention group, those who gained weight had better prognosis [5]. This suggested that weight gain was related to favourable response to cancer treatment and not to the tested intervention.

The increase in dietary intake was not efficient to prevent weight loss in our study. As in younger adults, [5] weight loss in these patients was probably mainly due to cancer disease within the framework of cancer cachexia and very unlikely due to other causes, as we hypothesized when constructing the trial.

The reason why increased energy intake fails to change body mass may involve the activation of anti-anabolic pathways during cancer cachexia [23], [24]. It is likely that pharmaconutrition targeting cachexia related metabolism alterations should be tested. This trial cannot however provide hypothesis to drive pharmaconutrition related to cancer cachexia.

In patients receiving chemotherapy, increase in dietary intake just before treatment may also not be favourable. Indeed, it has been reported that short-term fasting (48 hours) can protect normal cells but not cancer cells from chemotherapy agents in mice and in cell culture [25]. An interventional study in patients undergoing chemotherapy for breast or prostate cancer is ongoing to test the effects of a controlled low calorie diet on the side effects and response to treatment (NCT01802346).

The situation in patients receiving radiotherapy might be different from that in those with chemotherapy. In head and neck cancer patients, several trials found an improvement in dietary intake [26], quality of life [27], and nutritional status [28]. It is noticeable that we also found no differences in quality of life items (data not shown) among the intervention groups in surviving patients at the end of chemotherapy. A trial of dietary counselling or oral supplementation in 111 patients with colon cancer treated with radiotherapy showed the superiority of dietary counselling compared to oral nutritional supplements and to control on outcomes during treatment [29]. Mortality was not analyzed in these patients with good prognosis. Nutritional support in dysphagic patients is probably mandatory unlike in patients with anorexia owing to the cancer disease itself. In dysphagic patients with oesophageal cancer, enteral support led to the same prognosis as in patients with the same cancer but without swallowing problems [30]. The question of the prognostic value of anorexia and subsequent weight loss related to cancer cachexia is thus crucial. On one hand, it is very difficult to overcome anorexia; on the other, the intensity of anorexia may reflect the severity of the cancer disease [31].

In conclusion, individual dietary counselling in older patients at risk for malnutrition during their chemotherapy treatment for cancer was associated with an increase in dietary intake but did not decrease mortality.

## Supporting Information

Consort S1 **INOGAD Consort.**
(DOC)

Consort S2 **INOGAD CONSORT Abstract.**
(DOC)

Protocol S1 **Protocol extract.**
(DOC)

Protocol S2 **Protocol full in French.**
(PDF)
