# Supplementary material for: Nutritional Advice in Older Patients at Risk of Malnutrition during Treatment for Chemotherapy: A Two-Year Randomized Controlled Trial
Source: PLoS One. 2014 Sep 29;9(9):e108687. doi: 10.1371/journal.pone.0108687 (PMC4181649; doi:10.1371/journal.pone.0108687)
Supplement: Consort S2 — INOGAD CONSORT Abstract. (DOC) [file pone.0108687.s002.doc]

**Items to include when reporting a randomized trial in a journal or conference abstract**

| **Item** | **Description** | **Reported on line number** |
| --- | --- | --- |
| Title | Identification of the study as randomized | Title |
| Authors * | Contact details for the corresponding author |  |
| Trial design | Description of the trial design (e.g. parallel, cluster, non-inferiority) | Method |
| Methods |  |  |
| Participants | Eligibility criteria for participants and the settings where the data were collected | Method |
| Interventions | Interventions intended for each group | Method |
| Objective | Specific objective or hypothesis | Method |
| Outcome | Clearly defined primary outcome for this report | Method |
| Randomization | How participants were allocated to interventions | Method 8 |
| Blinding (masking) | Whether or not participants, care givers, and those assessing the outcomes were blinded to group assignment | Open label study |
| Results |  |  |
| Numbers randomized | Number of participants randomized to each group | Results |
| Recruitment | Trial status | Results |
| Numbers analysed | Number of participants analysed in each group | Results |
| Outcome | For the primary outcome, a result for each group and the estimated effect size and its precision | Results |
| Harms | Important adverse events or side effects | none |
| Conclusions | General interpretation of the results | Conclusion |
| Trial registration | Registration number and name of trial register | bottom |
| Funding | Source of funding | Not included in the abstract |

**this item is specific to conference abstracts*
