# Supplementary material for: Nutritional Advice in Older Patients at Risk of Malnutrition during Treatment for Chemotherapy: A Two-Year Randomized Controlled Trial
Source: PLoS One. 2014 Sep 29;9(9):e108687. doi: 10.1371/journal.pone.0108687 (PMC4181649; doi:10.1371/journal.pone.0108687)
Supplement: Protocol S1 — Protocol extract. (DOC) [file pone.0108687.s003.doc]

# Extracts of the French protocol translated for:

• Selection of patients, including both eligibility and ineligibility criteria

• Schema and treatment plan, including administration schedule

• Rules for dose modification

• Measurement of treatment effect including response criteria, definitions of response and survival, and methods of measurement

• Reasons for early cessation of trial therapy

• Objectives and entire statistical section (including endpoints)

1. OBJECTIves
   1. Main objective

The main objective is to assess the effects of an individualized nutritional support compared to usual care during their chemotherapy treatment, on one-year mortality in older patients (>70y) at risk for malnutrition according to full MNA.

- 1. Secondary objectives
- The secondary objectives are to assess the effects of this nutritional support applied to this population at risk for malnutrition on:
- Two-year mortality,
- Chemotherapy tolerance (grade 3-4 toxicities rate)
- Functional status (decline or improvement) at the end of chemotherapy
- Quality of life at the end of chemotherapy
- Nutritional status at the end of chemotherapy

And to study:

- Compliance
- Clinical and biological determinant of chemotherapy tolerance and survival.

1. Schéma and randomization
   1. Schéma / Méthods

This is an open label multicenter superiority randomized controlled clinical trial comparing Usual Care to Usual Care + Nutritional Intervention in two parallel arms:

- **« usual care »** **group**: usual care (according to the policies of the oncological team) and no nutritional support specific to the study
- **« usual care + specific nutritional intervention »** **group**: individualized nutritional support (performed by a dietician, from the first day and during the whole chemotherapy treatment, aiming to provide 30 kCal/kg/d or more and 1.2 g protein/kg/d)
  1. shedule
- Inclusion period : 36 months
- Participation duration for each participants: 24 months including initial chemotherapy duration, full assessment at the end of chemotherapy (3 to 4 months or 6 cycles), and survival follow-up with phone contact (at 12 month and utill 2 years).
- Total trial duration: 5 years

It is planned to start trial at first quarter 2007.

- 1. Randomization

The randomization list is established by the study statistician of the Methodological and Management Centre before trial start. The sizes of the 2 groups are balanced with 1:1 ratio. The randomization is stratified on recruitment centre. A descriptive document is confidentially preserved in Methodological and Management Centre.

The randomization iss centralized by internet *via* a study dedicated Internet site. This centralization permits to control eligibility criteria and to communicate to the investigator for each patient the informations related to randomization.

1. Eligibility critèria

The eligibility critèria, are checked during the pre-inclusion visit (visit 0) which is performed the same day of the inclusion visit (Visit 1) and the same day of the begin of the randomized intervention; this is also the begin of the chemotherapy treatment.

The free informed and written consent is collect at the latest, during the inclusion visit and before any procedure related to the trial.

- 1. pré inclusion Critèria
- Adults of both gender aged ≥ 70 ans at pré-inclusion visit
- Lymphoma or carcinoma histologically prouven
- Colon cancer with chemotherapy indication *(adjuvant or palliative)*
- Breast cancer with chemotherapy indication
- Stomach cancer with chemotherapy indication *(adjuvant or palliative)*
- Pancreas cancer with chemotherapy indication
- Biliary duct cancer: cholangiocarcinome with chemotherapy indication
- Ovary cancer with chemotherapy indication *(adjuvant or palliative)*
- Any B cell lymphoma, any T lymphoma
- Any low malignancy lymphomas such as follicular, lymphoplasmacytic, lymphocytic, mantle, MALT, and other marginal zone lymphoma
- Prostate cancer with chemotherapy indication
- Bladder cancer with chemotherapy indication
- Non small cell lung cancer with chemotherapy indication
- Adenocarcinoma with unknown origin but compatible with one of the above mentioned origin and with chemotherapy indication
- Cancer with chemotherapy indication up to the third line according to a routine protocol according to the good uses established in each of the treatment centre. The protocol list will be written before the start of the trial and updated each quarter.
- Life expectancy > 12 weeks, Karnofsky index over or equal to 50%
- Full MNA included in 17 et 23,5 : patients at risk for malnutrition.
- Patient with a social insurance.
  1. inclusion Critèria
- Preinclusion criteria
- Free informed and written consent signed by the patient and the investigator
  1. non inclusion criterai
- MNA < 17 ou ≥ 24
- Impossibility to follow-up for geographical, psychological or social reason: subjects for whom the treatment will be performed in a centre not allowing neither the dietician intervention or the gerontological assessments at the start and the end of treatment
- Symptomatic brain or meningeal metastasis,
- Patients with non routine chemotherapy treatment,
- Dosage or treatment schedules not compatible with the trial follow-up, such as lack of visit between 3 to 4 month and not allowing the nutritional intervention.
- Subject under guardianship,

1. Compared interventions

The compared intervention needs a priori standardization

For this purpose:

- In the « usual care » group

Before the start of the study, the good usual care practice for each treatment centre will be collected. It will be recommend following them

- In the « usual care + specific nutritional care » group

A training will be organised by the coordinating dietician and will be given to each of the study dietician in order to standardize the nutritional intervention specific of the study.

After written consent signature and checking of the inclusion criteria, the patients will be randomized in each of the following groups:

- 1. « usual care »  Group

The care is those uually performed in the centre.

The resort to dietician care is allowed according to the local uses but this help must be performed by a dietician different from the study dietician (the one in charge of the nutritional intervention specific to the study). Indeed, the dietician in charge of the « usual care + specific nutritional care » patients,  must neither see the patients of the « usual care » group, or give advices to the prescribing doctors.

The actual dietary intake will be assessed with self questionnaires collected during the first visit and each of the visits until the last one.

- 1. « usual care + specific nutritional care » group

The specific nutritional intervention begins at the very first visit (visit 1) and goes on during all the chemotherapy duration. It is given by a dietician (study dietician) which is not the one of the usual care group. She (or he) must neither see the patients of the « usual care » group, or give advices to the prescribing doctors

The talk with the study dietician aims to ensure 30 kCal/kg/d or more and 1.2 g proteins/kg/d.

The study dietician must implement the means adapted to functional autonomy (ADL and IADL) of the patient to help. For this the geriatric team will help. The adequacy of the nutritional advises will be assess with a phone call the week following the first visit.

The subjects will be seen by the study dietician at each chemotherapy cycle if the schedule allows a visit each 2 weeks. If the cycles are spaced by 3 weeks or more, a phone call will be performed in between. The compliance to the intervention will be assessed with numbering visits and phone calls.

In practice, the study dietician sees the patient at each chemotherapy session, En pratique, gives phone call if pertinent and adapts advises to nutritional problems of the patient untill the last visit at the end of chemotherapy.

The dietary intake will be collected by investigators with a standardized questionnaire at each visit and interpreted by the study dietician.

In both groups, the dietary intake assessments with the standardized questionnaires will be done by the coordinating dietician.

Rules for dose modification are not applicable here

1. Associated treatments

Surgery and radiotherapy are authorized along with any associated treatments considered as necessary to the patient care.

The oncologists are free to give advises and nutritional supplements or artificial nutrition if they estimate them necessary to the care according to the evolution of the health status of the patient..

The ESPEN guidelines for artificial nutrition in patients with cancer and treated with non-surgical treatment should be applied. In pertinent, indication and follow-up will be documented.

1. endpoints
   1. principal endpoint

The main endpoint is the one-year survival after randomization (start of the nutritional intervention).

Vital status will be assessed after talk with medical practionners. Any death should be declared by the medical practitionner within 15 days. A phone contact is planned each 6 months with the patient, relatives to collect vital status and to anticipate any address move.

Any potential information way will be used to assess vital status if the patient is lost of follow-up before the end of the first year, in particular national cancer registry and the RNIPP (Répertoire National d’Identification des Personnes Physiques, national registry of identification of physical persons).

- 1. secondary enpoints

The secondary endpoints are:

- Two-year survival,
- Chemotherapy tolerance : assessed with the toxicities scale (NCI CTCv3) at each chemotherapie session (each visit from V1 to End Visit),
- Thymia (GDS) (each visit from V1 to End Visit),
- Fonction : IADL, ADL, get up and go test, one leg stance (V1 and End Visit),
- Quality of life  (QLQ–C30) (V1 and End Visit),
- MNA (V1 and End Visit),
- Weight (each visit from V1 to End Visit)
- Biology : CRP, albumin, haemoglobinémia (V1 and End Visit),
- Dietary intake (each visit from V1 to End Visit):
  - - 24-h recall method with auto-questionnaire with the help of care giver if pertinent
    - Number and type of nutritional supplements,
    - Artificial nutrition (type, volume, quality, duration, complications),
- Hospitalisations (number and number of days),
- Infections with clinical symptomatology (feber, dysuria, dyspnea, antibiotics),
- Mortality during chemotherapie period,
- Others : fractures, falls, pressure ulcers.
  1. recapitulative table of patient follow-up

|  | Pre-inclusion | Inclusion | Follow-up visits | | |  |  |
| --- | --- | --- | --- | --- | --- | --- | --- |
|  | Visite 0 | Visite 1  (1^er^ cycle de chimiothérapie) | Visite 2  (2^ième^ cycle de chimiothérapie) | … | Visite N  (N^ième^ cycle de chemotherapy) | End visit  (end of chemotherapy) | Visite 1 et 2ans  Téléphone ou courrier |
| Information | **X** |  |  |  |  |  |  |
| Informed consent |  | **X** |  |  |  |  |  |
| MNA | **X** |  |  |  |  | **X** |  |
| Comprehensive Gerontological assessment^1^ |  | **X** |  |  |  | **X** |  |
| Weight |  | **X** | **X** | **X** | **X** | **X** |  |
| Dietary intake^5^ |  | **X** | **X** | **X** | **X** | **X** |  |
| Oncological assessmentoncologique^2^ |  | **X** | **X** | **X** | **X** | **X** |  |
| Biology^3^ |  | **X** |  |  |  | **X** |  |
| Serotheque^4^ |  | **X** |  |  |  | **X** |  |
| Vital status |  | **X** | **X** | **X** | **X** | **X** | **X** |

^1^ function (ADL, IADL Get Up and Go test, 3month fall recall), cognition (MMSe), thymia (GDS), quality of life (QLQ-C30**), non mandatory assessment**

^2^ Initial cancer description, co-morbidity at baseline and chemotherapy assessment at each visit

^3^ see detailed biology variabeles

^4^ Conditionnée au niveau de chaque centre

^5^ 24h dietary recall method

1. Statistical ASPECTS
   1. statistical Méthods
      1. strategy

An interim analysis is planned when half of the participants are evaluated for the main outcome, at the end of the first year of follow-up. This analyse targets the main outcome, one-year survival. This analysis allows on one side to estimate confidence interval of difference between groups, and on the other side to test the existence of an important difference between the two groups in order to take the decision of trial stooping to generalize as soon as possible the specific nutritional intervention of the trial to all subjects at risk for malnutrition if it is able to increase survival. To preserve the global risk α of 5%, the O’Brien & Fleming method will be used for intermediary and final analysis.

Main analysis is performed

- on intention to treat basis, i.e. all randomized patients will be included in the initial allocated group and all of their data will be used whatever changes in intervention occurring during the trial.
- Using the strategy « missing data = failure » : any missing data will be replaced with the failure, here the death.

This analysis will be completed by a sensitive analysis for missing data using the strategy of maximum bias.

Description will be done globaly and for each intervention groups.

Comparison between groups are systematically performed :

1) without ajustement,

2) with adjustment on stratification variable : centre : duration of artifical nutrition and chemotherapy type

All tests are done with error risk of first type α =5%.

- - 1. Software

The analysis are realized with sont réalisées SAS®  software (version n°9.1).

- 1. Determination of sample size

In this trial, patients are randomized into 2 groups:

- group 1 (« usual care »  Group) : no study specific nutritional support
- group 2 (« usual care + specific nutritional care » group)

Comparison of the groups is about the proportion of deceased patients one-year after baseline. The trail is a superiority trial, bilateral test. An interim analysis is planned when half of the participants are evaluated for the main outcome, one year after randomization.

We hypothezised that proportion of deceased patients in usual care group will be 50% one-year after inclusions according to the preliminary results of an oncogeriatric cohort of 364 subjects assessed with comprehensive gerontological assessment, and we consider that specific nutritional intervention assessed in this trial (individualized nutritional support) will be of clinical interest if a reduction of at least 10% in one-year mortality is observed.

On the basis of the O’Brien and Fleming rule to take into account the interim analysis, an alpha 5% risk and a 1-beta 80% power, with an interim analysis, and loss of follow-up expected lower than 5%, at least 390 patients in each group, in total 780 had to be enrolled

To take into account a proportion of lost of follow-up lower than 5%, we decide to enrol a total of 820 patients, 410 per group.

- 1. Plan of analysis
     1. Description of included patients and follow-up

Pre-included and non included patients in the trial are described and compared to included patients for socio-démographic data and cancer types.

The number of included patients, inclusions curve (evolution of included patients between the first and the last patient included), the number of theoretical visits corresponding to the number of included patients, the number of actually performed visits, and the ratio of both number of actually performed visits / number of theoretical visits) are shown according to groups. The cumulative duration of follow-up is calculated (sum of participation duration for each included patient, i.e. the difference in number of days between inclusion date and last news date in the trial) and the ratio cumulative follow-up duration/ expected cumulative follow-up duration is presented..

- - 1. Patients included in analysis

The exclusive causes of exclusion of analyses are:

- Patients wrongly included without informed written consent,
- Patients wrongly included because of non respect of major exclusion Patients having withdrawn consent.

This exclusion decision is taken by the scientific committee after documentation of the observation by Methodological and Management Centre blind for group allocation and follow-up data.

Lost of follow-up patients or having given-up the trial are included in the analysis.

- - 1. patients Caractéristics at inclusion, before the intervention start

The patients are described for following variables:

- respect of eligibility criteria;

- epidemiological characteristics;

- clinical characteristics;

- biological characteristics;

- treatment characteristics;

A description of protocol violations and patients distribution according them is done.

A description of abandon causes is realized in patients lost for follow-up or having given up the trial.

- - 1. description of usual care

For each centre, the usual nutritional care is written.

- - 1. Principal outcome

The proportions of deaths are described as number, percent, confidence interval and compared between intervention groups with a χ^2^ test or corrected χ ^2^ to the number of expected values according to independence hypothesis.

Surival curves are estimated according to survival probability and confidence interval with Kaplan Meyer method. The date of origin is the randomization day and the delay used in the analysis is the difference between randomization date and the death date or last follow-up visit date before one year if the patient is lost for follow-up despite adequate procedure to collect vital status. The two groups are compared using the log rank test.

- - 1. secondary outcome(s)

Numbers, proportions, and confidence interval are used to describe qualitative variables of the secondary outcomes. Comparisons were made with the Chi2 test or corrected χ ^2^ or exact Fisher test according to the expected values according to independence hypothesis..

Quantitative variables are described with mean and standard deviation (SD), and compared using Student’s t test or the Wilcoxon test according to the distribution of the analysed variable. Les variables quantitatives sont décrites en termes d’effectif, moyenne, écart-type et intervalle de confiance de la moyenne, médiane, étendue et étendue interquartile. Variables could be transformed if necessary.

Clinical and biological prognosis factors of chemotherapy tolerance and survival are studied with logistical regression model or Cox model according to the type of explained variable.

If appropriated a graphic representation will be associated to the analysis.

All tests are done with a primary type risk α = 5% (bilateral test).
