# Supplementary material for: Nutritional Advice in Older Patients at Risk of Malnutrition during Treatment for Chemotherapy: A Two-Year Randomized Controlled Trial
Source: PLoS One. 2014 Sep 29;9(9):e108687. doi: 10.1371/journal.pone.0108687 (PMC4181649; doi:10.1371/journal.pone.0108687)
Supplement: Protocol S2 — Protocol full in French. (PDF) [file pone.0108687.s004.pdf]

Intervention Nutritionnelle en Onco-Gériatrie chez des patients A risque de Dénutrition; Etude interventionnelle randomisée

INOGAD

PROTOCOLE D'ETUDE CLINIQUE

Version n°1.11 du 11 avril 2007
ayant reçu l'avis favorable du CPP Sud-Ouest et Outre Mer III le 31 janvier 2007

Numéro ID RCB 2006-A00682-49
Numéro d'enregistrement dans **http://www.clinicaltrials.gov** : ***à compléter***

Promoteur :

Centre Hospitalier Universitaire de Bordeaux
12 rue Dubernat,
33 400 TALENCE
Tel : 05 56 79 47 98
Fax : 05 56 79 49 26

Investigateur Coordonnateur :
Pr Isabelle Bourdel-Marchasson
Département de gériatrie
Centre de gériatrie Henri Choussat, Hôpital Xavier Arnozan
33604 PESSAC Cedex
Tel : 05 57 65 65 71 - Fax : 05 57 65 65 60
E-mail: isabelle.bourdel-marchasson@chu-bordeaux.fr

Centre de Méthodologie et de Gestion :
Pr Geneviève CHENE
Unité de Soutien Méthodologique à la Recherche Clinique et Epidémiologique du CHU de Bordeaux,
146 rue Léo-Saignat, case n°11
33076 BORDEAUX Cedex

Tel : 05 57 57 11 29/12 57 - Fax : 05 57 57 11 78
Courriel : genevieve.chene@isped.u-bordeaux2.fr

PRINCIPAUX CORRESPONDANTS

Investigateur Coordonnateur
Pr Isabelle BOURDEL-MARCHASSON
Centre de gériatrie Henri Choussat,
Hôpital Xavier Arnozan
33604 Pessac Cedex
Tel : 05 57 65 65 71 - Fax : 05 57 65 65 60
E-Mail : isabelle.bourdel-marchasson@chu-bordeaux.fr

Co-Investigateur Coordonnateur
Dr Marianne FONCK
Institut Bergonié,
229 Cours de l'Argonne,
33076 Bordeaux Cedex
Tel : 05 56 33 32 42- fax : 05 56 33 33 83
E-Mail: Fonck@bergonie.org

Co-Investigateurs
Pr Muriel RAINFRAY
Centre de gériatrie Henri Choussat,
Hôpital Xavier Arnozan
33604 Pessac Cedex
Tel : 05 57 65 65 57 - Fax : 05 57 65 65 60
E-mail : muriel.rainfray@chu-bordeaux.fr

Dr Pierre SOUBEYRAN
Institut Bergonié,
229 Cours de l'Argonne
33076 Bordeaux Cedex
Tel : 05 56 33 32 43- fax : 05 56 33 33 83
E-Mail : Soubeyran_p@bergonie.org

Coordination Diététique
Mme M-Dominique DANIEL-LAMAZIERE
Hôpital Haut-Lévêque
Avenue Magellan
33604 Pessac cedex
E-Mail : dominique.daniel-lamaziere@chu-bordeaux.fr

ARC Coordonnateur
Dr Christèle BLANC-BISSON
Centre de gériatrie Henri Choussat,
Hôpital Xavier Arnozan
33604 Pessac cedex
Tel : 05 57 65 65 65 poste 30045
Fax : 05 57 65 65 60
E-Mail :christele.blanc-bisson@chu-bordeaux.fr

Promoteur
Centre Hospitalier Universitaire de Bordeaux
12 rue Dubernat,
33 400 Talence - FRANCE
Tél. : 05 56 79 47 98- Fax : 05 56 79 49 26

Centre de Méthodologie et de Gestion
Unité de **S**outien **M**éthodologique à la **R**echerche Clinique et Epidémiologique du CHU de Bordeaux
146 rue Léo-Saignat, case n°11
33076 Bordeaux Cedex - FRANCE

- Coordination méthodologique
Professeur Geneviève CHENE
Tél : 05 57 57 11 29- Fax : 05 57 57 15 78
courriel : genevieve.chene@isped.u-bordeaux2.fr

- Attachée de Recherche Clinique Référente
Sandrine DESJARDINS
Tél : 05 57 57 45 76 - Fax : 05 57 57 15 78
courriel :sandrine.desjardins@isped.u-bordeaux2.fr

- Statisticienne
Christine GERMAIN
Tél : 05 57 57 45 14 - Fax : 05 57 57 15 78
courriel : christine.germain@isped.u-bordeaux2.fr

- Analyste programmeur
Guillaume DUPOUY
Tél : 05 57 57 15 73 - Fax : 05 57 57 15 78
courriel : guillaume.dupouy@isped.u-bordeaux2.fr

- Secrétariat
Karine SURLANNE
Tél : 05 57 57 11 29 - Fax : 05 57 57 15 78
courriel : karine.surlanne@isped.u-bordeaux2.fr

SOMMAIRE

1. RESUME **7**

2. JUSTIFICATION **11**

2.1. ETAT ACTUEL DES CONNAISSANCES 11
2.1.1. L'impact de la dénutrition sur le pronostic de la maladie néoplasique 11
2.1.2. Paramètres nutritionnels et chimiothérapie 12
2.1.3. L'intervention nutritionnelle en cancérologie 12
2.1.4. Malnutrition et sujet âgé 13
2.2. HYPOTHESES DE L'ETUDE 14
2.3. SOUTIEN NUTRITIONNEL SPECIFIQUE A L'ETUDE 15
2.4. JUSTIFICATION DES CHOIX METHODOLOGIQUES 16

3. OBJECTIFS **16**

3.1. OBJECTIF PRINCIPAL 16
3.2. OBJECTIFS SECONDAIRES 16

4. SCHEMA ET RANDOMISATION **16**

4.1. SCHEMA / METHODES 16
4.2. ECHEANCIER DE L'ESSAI 16
4.3. RANDOMISATION 17

5. CRITERES D'ÉLIGIBILITE **17**

5.1. CRITERES DE PRE INCLUSION 17
5.2. CRITERES D'INCLUSION 17
5.3. CRITERES DE NON INCLUSION 18

6. INTERVENTIONS COMPAREES **18**

6.1. GROUPE SOINS USUELS 18
6.2. GROUPE SOINS USUELS PLUS INTERVENTION NUTRITIONNELLE SPECIFIQUE 18

7. TRAITEMENTS ASSOCIES **19**

8. CRITERES DE JUGEMENT **19**

8.1. CRITERE DE JUGEMENT PRINCIPAL 19
8.2. CRITERES DE JUGEMENT SECONDAIRES 19

9. DEROULEMENT DE L'ESSAI **20**

9.1. MODALITES DE RECUEIL DU CONSENTEMENT 20
9.2. TABLEAU RECAPITULATIF DU SUIVI PATIENT 21
9.3. VISITE DE PRE INCLUSION (VISITE 0) 21
9.4. VISITE D'INCLUSION (VISITE 1) 21
9.4.1. Recueil du consentement 21
9.4.2. Randomisation 21
9.4.3. Déroulement de la visite 22
9.5. VISITES DE SUIVI 22
9.6. VISITES FC (FIN DE CHIMIOTHERAPIE) 23
9.7. VISITES A 1 ET 2 ANS 23
9.8. CONDUITE A TENIR EN CAS DE NUTRITION ARTIFICIELLE 23
9.9. DEVIATIONS AU PROTOCOLE 23
9.9.1. Arrêt prématuré et définitif de la stratégie d'intervention de l'essai 23
9.9.2. Patient perdu de vue 23
9.9.3. Autres déviations 24
9.10. RETRAIT DE CONSENTEMENT ET ABANDON 24

9.10.1. Abandon *24*
9.10.2. Retrait de consentement *24*

10. ÉVÉNEMENTS INDÉSIRABLES 24

10.1. EVENEMENTS INDESIRABLES 24
10.1.1. Définitions *24*
10.1.2. Notification d'un évènement indésirable non grave *24*
10.1.3. Suivi d'un évènement indésirable non grave *24*
10.2. EVENEMENTS INDESIRABLES GRAVES (EIG) 25
10.2.1. Définitions *25*
10.2.2. Notification et suivi des Evènements Indésirables Graves *25*

11. SURVEILLANCE DE L'ESSAI 26

11.1. CONSEIL SCIENTIFIQUE 26
11.1.1. Composition *26*
11.1.2. Rythme des réunions *26*
11.1.3. Rôle *26*
11.2. COMITE INDEPENDANT DE SURVEILLANCE 27
11.2.1. Composition *27*
11.2.2. Rythme des réunions *27*
11.2.3. Rôle *27*
11.3. COMITE DE VALIDATION DES EVENEMENTS 27
11.3.1. Composition *27*
11.3.2. Rythme des réunions *27*
11.3.3. Rôle *27*
11.4. CENTRE DE METHODOLOGIE ET DE GESTION 27
11.4.1. Composition *27*
11.4.2. Rythme des réunions *28*
11.4.3. Rôle *28*

12. ASPECTS STATISTIQUES 28

12.1. METHODES STATISTIQUES EMPLOYEES 28
12.1.1. Stratégie d'analyse *28*
12.1.2. Logiciels utilisés *29*
12.2. CALCUL DE LA TAILLE D'ETUDE 29
12.3. PLAN D'ANALYSE 29
12.3.1. Description des inclusions et du suivi *29*
12.3.2. Patients inclus dans l'analyse *29*
12.3.3. Caractéristiques des patients à l'inclusion, avant le début de l'intervention *29*
12.3.4. description des prises en charge usuelles *30*
12.3.5. Critère de jugement principal *30*
12.3.6. Critère(s) de jugement secondaire(s) *30*

13. ASPECTS ETHIQUES ET RÉGLEMENTAIRES 30

13.1. CONFORMITE AUX TEXTES DE REFERENCE 30
13.2. AMENDEMENT AU PROTOCOLE 31
13.3. CONFIDENTIALITE DES DONNEES 31
13.4. ASSURANCE 31
13.5. ARCHIVAGE ET STOCKAGE DES DOCUMENTS A LA FIN DE L'ETUDE 31
13.6. ENREGISTREMENT DE L'ESSAI 32

14. MONITORAGE ET BONNES PRATIQUES CLINIQUES 32

14.1. ORGANISATION GENERALE 32
14.1.1. Rôle de l'ARC référent de l'USMR *32*
14.1.2. Rôle de l'ARC du centre investigateur coordonnateur *32*

14.2. CONSIGNES POUR LE RECUEIL DES DONNEES 33
14.3. MONITORAGE DES CENTRES INVESTIGATEURS 33
14.4. AUDIT - INSPECTION 33

15. PUBLICATION DES RESULTATS 33

16. AUTRES RESULTATS ATTENDUS 34

17. CESSION DES DONNEES 34

18. REFERENCES BIBLIOGRAPHIQUES 35

ANNEXES 38

ANNEXE 1 NOTE D'INFORMATION – FORMULAIRE DE CONSENTEMENT 38
ANNEXE 2 : AVIS DU CPP 44
ANNEXE 3 : LISTE DES CENTRES INVESTIGATEURS 45
ANNEXE 4 :QUESTIONAIRES D'EVALUATION GERIATRIQUE STANDARDISEE 47
ANNEXE 5 : DECLARATION D'HELSINKI 59
ANNEXE 6 : CRITÈRES DE TOXICITÉ (NCI-CTC V3.0) 62

PAGE DE SIGNATURE DU PROTOCOLE

Intervention Nutritionnelle en Onco-Gériatrie chez des patients A Risque de Dénutrition

ETUDE INOGAD

Version n°1.11 du 11 avril 2007
ayant reçu l'avis favorable du CPP Sud Ouest et Outre Mer III le 31 janvier 2007

Promoteur

Centre Hospitalier Universitaire de Bordeaux à Talence, le : <*date*>

12 rue Dubernat,

33 400 Talence

Tel : 05 56 79 47 98

Fax : 05 56 79 49 26

Le Directeur Général du CHU de Bordeaux

A. HERIAUD

Par délégation, le Directeur des Affaires Médicales et de la Recherche Clinique,

J.F. VINET

Investigateur coordonnateur : à Pessac, le : <*date*>

Pr Isabelle Bourdel-Marchasson

Centre de gériatrie Henri Choussat,

Hôpital Xavier Arnozan

33604 PESSAC Cedex

Tel : 05 57 65 65 71

Fax : 05 57 65 65 60

Pr Isabelle Bourdel-Marchasson

1. RESUME

Titre : Intervention Nutritionnelle en Onco-Gériatrie chez des patients A Risque de Dénutrition; Etude interventionnelle randomisée: INOGAD

- **Version :** 1.11 du 11/04/2007
- **Promoteur** : CHU de Bordeaux
- **Investigateur coordonnateur** : Pr. Isabelle Bourdel-Marchasson
- **Justificatif de l'étude** :

La malnutrition touchant les patients porteurs de cancer, appelée cachexie tumorale, atteint approximativement 50 % d'entre eux durant l'évolution de leur maladie. La prévalence la plus élevée est observée chez les patients atteints de localisations tumorales digestives. Il est établi que la perte de poids est un facteur de mauvais pronostic pour les patients recevant une chimiothérapie pour des cancers digestifs. A notre connaissance, il existe un nombre limité d'études du rôle de la correction de la dénutrition pour améliorer le pronostic. La plupart de ces études concerne des patients opérés et la proportion de sujets âgés y est peu décrite. Une première étude descriptive d'oncogériatrie menée par l'équipe qui présente le projet a réuni en Aquitaine 364 patients âgés en indication de chimiothérapie suivis sur une médiane de 13 mois. Elle est le résultat d'une collaboration large entre les oncologues et les gériatres d'Aquitaine, appartenant à des structures publiques ou privées. L'analyse intermédiaire des résultats des 155 premiers patients inclus d'une étude onco-gériatrique réalisée en Aquitaine, a montré qu'à l'évaluation gérontologique (EGS) initiale, 28 (18,7 %) étaient dénutris (MNA<17) et 72 (48,0 %) à risque de dénutrition (MNA 17-23,5). A la fin du suivi dont la médiane était de 1 an, la mortalité était d'autant plus importante que l'atteinte nutritionnelle était initialement grave : 20 (71,4%) des dénutris étaient décédés, 34 (47,2 %) des patients à risque de dénutrition et 13 (26,0 %) des 50 sujets non identifiés comme ayant des problèmes nutritionnels. Le soutien nutritionnel systématique des patients cancéreux dénutris fait d'ores et déjà l'objet d'un consensus, tandis que la conduite à tenir pour les patients dits à risque de dénutrition n'est pas clairement établie. Nous faisons l'hypothèse que le soutien nutritionnel des patients à risque de dénutrition et détectés lors de l'EGS pourrait améliorer leur état nutritionnel ou freiner sa dégradation pendant le traitement. Cette préservation de l'état nutritionnel pourrait améliorer la survie, la tolérance du traitement ainsi que l'état fonctionnel et la qualité de vie de ces patients.

- **Objectif principal** : L'objectif principal est d'évaluer les effets sur la survie à un an d'un soutien nutritionnel individualisé par rapport à la prise en charge habituelle chez les patients âgés (> 70 ans) à risque de dénutrition selon le MNA (Mini Nutritional Assessment) pendant leur traitement oncologique.
- **Objectifs secondaires :** Les objectifs secondaires sont d'évaluer les effets de ce soutien nutritionnel appliqué à cette population de sujets à risque de dénutrition sur :
  - la survie à deux ans
  - la tolérance de la chimiothérapie (fréquence des toxicités sévères ou de grade 3-4)
  - l'état fonctionnel (maintien ou amélioration) en fin de traitement par chimiothérapie
  - la qualité de vie en fin de traitement par chimiothérapie
  - l'état nutritionnel en fin de traitement par chimiothérapie

et d'étudier :
  - l'observance
  - les facteurs pronostiques cliniques et biologiques de la tolérance de la chimiothérapie et de la survie.

- **Méthode** :

Essai clinique randomisé de supériorité, sans insu, multicentrique (Cancéropole du grand Sud-Ouest) comparant 2 groupes en parallèle (groupe « soins usuels» vs groupe « soins usuels + intervention nutritionnelle spécifique »)

- **Principaux critères d'éligibilité** :
  - **Pré-inclusion**

  Adultes des 2 sexes âgés de ≥ 70 ans à la visite de pré inclusion ; Lymphome ou carcinome histologiquement prouvé ; cancer du colon en indication de chimiothérapie *(adjuvante ou palliative)* ;cancer du sein en indication de chimiothérapie ; cancer de l'estomac en indication de chimiothérapie *(d'induction ou palliative)* ; cancer du pancréas en indication de chimiothérapie ; cancer de l'ovaire en indication de chimiothérapie *(adjuvante ou palliative)* ; tous lymphomes diffus à

grandes cellules B, tous lymphomes T périphériques; tous lymphomes de faible malignité : lymphocytiques, lympho-plasmocytaires, folliculaires, manteau, zone marginale (MALT et autres) ; Cancer de la prostate en indication de chimiothérapie ; Cancer de la vessie en indication de chimiothérapie ; Cancer bronchique en indication de chimiothérapie ; Adénocarcinome de primitif inconnu compatible avec une origine citée ci-dessus en indication de chimiothérapie ; Cancer relevant d'un traitement par chimiothérapie jusqu'à la troisième ligne de chimiothérapie selon un protocole selon un protocole conforme aux accords de bon usage établis au sein de chaque établissement. La liste de ces protocoles sera établie au sein de chaque établissement avant le démarrage de l'étude et mise à jour tous les 3 mois ; Espérance de vie > 12 semaines, l'Index de Karnofsky doit être supérieur ou égal à 50% ; MNA compris entre 17 et 23,5 : patients à risque de dénutrition ; Patient affilié ou bénéficiaire d'un régime de sécurité sociale.

- **Inclusion**

Critères de pré-nclusion et consentement libre, éclairé et écrit signé par le patient et l'investigateur

- **Critères de non inclusion :**

MNA < 17 ou ≥ 24 ; Impossibilité de suivi pour raison géographique, psychologique ou sociale : les sujets dont le traitement de chimiothérapie se ferait dans un centre ne permettant ni l'intervention de la diététicienne, ni les évaluations gérontologiques de début et de fin d'essai ; Métastases cérébrales ou méningées symptomatiques ; Patients sous protocoles de chimiothérapie non publiés sous forme d'article ; Posologie et schéma de traitement non compatibles avec le suivi, comme l'absence de visite entre 3 et 4 mois et ne permettant donc pas les évaluations gérontologiques de début et de fin de chimiothérapie, ni l'intervention nutritionnelle spécifique ; Personnes placées sous sauvegarde de justice,

- **Interventions comparées de l'essai :**

- groupe « soins usuels » : soins usuels (prise en charge habituelle de l'équipe d'oncologie) et pas de soutien nutritionnel spécifique à l'étude
- groupe « soins usuels + intervention nutritionnelle » : soutien nutritionnel individualisé (réalisé par une diététicienne, dès la première visite et pendant toute la durée de la chimiothérapie, visant à assurer un apport de 30 kCal/kg/j ou plus et 1,2 g protides/kg/j)

- **Schéma de l'étude :**

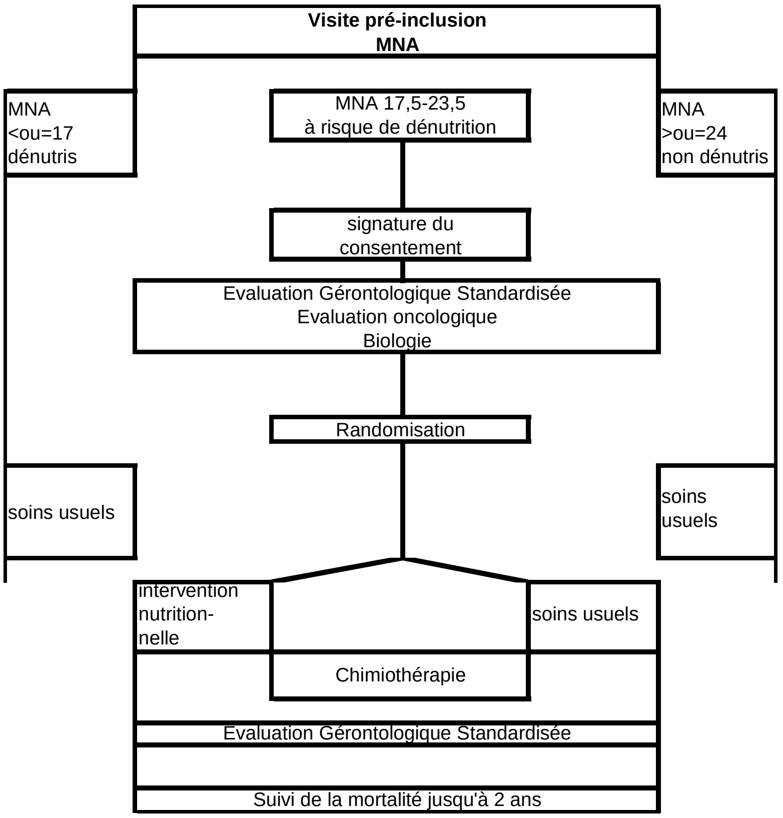

- **Critère de jugement principal :**
  - Survie à un an
- **Principaux critères de jugement secondaires:**
  - Survie à 2 ans,
  - Tolérance de la chimiothérapie : évaluée par l'échelle des grades de toxicité selon la NCI CTCv3 à chaque visite de chimiothérapie,
  - Thymie (GDS) (à chaque visite de V 1 et V FC),
  - Fonction : IADL, ADL, get up and go test, appui monopodal (V1 et V FC),
  - Qualité de vie (QLQ–C30) (V 1 et V FC),
  - MNA (V 1 et V FC),
  - Poids (à chaque visite de V 1 à V FC)
  - Biologie : CRP, albumine, hémoglobine (V 1 et V FC),
  - Enquêtes alimentaires (à chaque visite de V 1 à V FC) pour évaluer l'observance :
    - Méthode du rappel des 24 heures en auto-questionnaire avec éventuellement aide d'un accompagnant,
    - Nombre et type de suppléments consommés,
    - Alimentation artificielle (type, quantité, qualité, durée, complications),
  - Hospitalisations (nombre de jours),
  - Infections de traduction clinique générale (fièvre, dysurie, dyspnée, nécessité d'antibiothérapie),
  - Mortalité pendant la phase de chimiothérapie, et à deux ans
  - Autres : fractures, chutes, escarres.

- **Taille prévue de l'étude : 820** patients, soit 410 dans chaque groupe. Ceci représente environ 1640 sujets devant passer un MNA

- **Stratégie d'analyse**

Une analyse intermédiaire est prévue lorsque la moitié des patients inclus sera parvenue à la fin du recueil du critère de jugement principal, soit 1 an après avoir été inclus dans l'essai. Afin de préserver un risque global α de 5%, on utilise la méthode d'O'Brien & Fleming pour réaliser l'analyse intermédiaire et l'analyse finale.
L'analyse principale est effectuée en intention de traiter.

- **Echéancier :**

- Période d'inclusion : deux ans
- Durée de participation par patient : deux ans de suivi du statut vital (dont 3 à 4 mois de traitement ou 6 cycles de chimiothérapie)
- Durée de l'essai : 4 ans

- **Tableau récapitulatif du suivi des patients**

	Pré-inclusion	Inclusion	Visites de suivi				
	Visite 0	Visite 1 (1er cycle de chimiothérapie)	Visite 2 (2ème cycle de chimiothérapie)	…	Visite N (Nème cycle de chimiothérapie)	Visite FC (fin de chimiothérapie)	Visite 1 et 2ans Téléphone ou courrier
Consentement éclairé		X					
MNA	X					X	
Evaluation gérontologique[1]		X				X	
Poids		X	X	X	X	X	
Enquête diététique		X	X	X	X	X	
Evaluation oncologique[2]		X	X	X	X	X	
Bilan biologique[3]		X				X	
Sérothèque [4]		X				X	
Statut vital		X	X	X	X	X	X

[1] Automonie physique (ADL, IADL risque de chute évalué par version chronométrée du Get Up and Go test, recueil de nombre de chutes dans les 3 mois précedents), fonctions cognitives (par MMS), humeur (par GDS), qualité de vie (QLQ-C30), enquête alimentaire par la méthode de rappels des 24 heures

[2] Description initiale du cancer, recueil des co-morbidités à l'inclusion et évaluation de la tolérance de la chimiothérapie à chaque cycle détaillée dans le paragraphe correspondant

[3] CRP, albumine, hémoglobine

[4] Au niveau de chaque centre

- **Résultats et retombées attendues**

- Evaluation de l'impact d'une intervention individualisée selon un niveau élevé de preuve
- Etablissement de protocole de soutien nutritionnel des patients suivis en oncogériatrie

2. JUSTIFICATION

2.1. ETAT ACTUEL DES CONNAISSANCES

La malnutrition touchant les patients porteurs de cancer, appelée cachexie tumorale, atteint approximativement 50 % d'entre eux durant l'évolution de leur maladie (1) en raison d'une part du caractère hypercatabolique de ces pathologies et d'autre part de l'impact du cancer et de son traitement sur le comportement alimentaire et l'appareil digestif. La prévalence la plus élevée est observée chez les patients atteints de localisations tumorales digestives, notamment l'œsophage, l'estomac et le pancréas (2). Les cancers font donc partie des pathologies à la source de cachexie : anorexie, asthénie et diminution progressive de la masse corporelle liée à un état inflammatoire chronique (3). Les causes de cette dénutrition sont multiples (1) associant d'une façon variable selon le cancer et le patient :

- L'anorexie, symptôme présent dans plus de 50 % des cas au cours des cancers, et dont l'origine n'est en fait pas explicitée et pas toujours reliée à l'hypercatabolisme : relation avec les cytokines pro-inflammatoires ? (4)
- Les troubles de la déglutition, les obstacles anatomiques ou fonctionnels sur le tube digestif ou les douleurs induites par la prise alimentaire, en rapport avec la tumeur, la radiothérapie (5) ou les suites chirurgicales (6),
- Les perturbations des métabolismes glucidiques, lipidiques et protéiques : augmentation de la néoglucogenèse, insulino-résistance, augmentation des taux circulants d'acides gras libres, traduisant un état hypercatabolique. Une augmentation des dépenses énergétiques et de la consommation d'oxygène en découle pour certains d'entre eux sans que cela soit spécifique d'un cancer donné (7),
- La consommation métabolique de la tumeur elle-même qui favoriserait la néoglucogenèse (8). Le métabolisme tumoral en devenant préférentiellement anaérobie génèrerait plus de lactate dont le métabolisme hépatique (cycle de Cori) activerait la néoglucogenèse aux dépends des acides gras et des acides aminés. Ceux-ci obtenus respectivement à partir du tissu adipeux et du tissu musculaire expliquerait la fonte de ces deux compartiments. La sécrétion par la tumeur d'un proteoglycane induisant un hypercatabolisme musculaire a été montrée chez l'animal sans confirmation chez l'homme (9). L'action lipolytique d'un facteur extrait d'urines de patients cancéreux cachectiques a également été montrée sur la souris (10).
- Les perturbations nutritionnelles plus ou moins sévères liées aux traitements antinéoplasiques (atteintes des muqueuses, nausées, vomissements ou diarrhées après chimiothérapie et / ou radiothérapie).

2.1.1. L'IMPACT DE LA DENUTRITION SUR LE PRONOSTIC DE LA MALADIE NEOPLASIQUE

La relation entre dénutrition et pronostic a été établie dans plusieurs études. Au sein d'une cohorte de patients de 38 à 90 ans atteints de cancer pulmonaire, ceux qui ont la meilleure survie sont ceux qui ont les valeurs les plus élevées pour le pli cutané tricipital reflétant la masse grasse. Toutefois cette valeur pronostique disparaît quand on ajuste l'analyse sur les autres caractéristiques des patients, telles le stade du cancer, l'« ECOG performance status », l'albuminémie et l'haptoglobine, suggérant que l'état nutritionnel est un reflet de la gravité de l'atteinte du patient (11). Dans un groupe de 66 patients atteints de cancer pulmonaire dont l'âge n'était pas précisé, le pronostic en analyse multivariée était lié à un taux élevé d'IL-6, à un taux bas d'interféron γ, à un âge avancé et au caractère métastatique de la tumeur. Le caractère péjoratif de la perte de poids ou d'un bas BMI, de l'hypoalbuminémie, visible en analyse univarié n'était plus significatif en multivarié (12).Un taux bas de leptine est également un facteur de mauvais pronostic dans les cancers pulmonaires, le seul étant fixé à 2,4µg/l (13). Pour ces 76 patients, ce taux était inversement corrélé à l'intensité de la réaction inflammatoire et d'autant plus bas que la masse grasse était basse. En revanche, il est établi que la perte de poids est un facteur de mauvais pronostic pour les patients recevant une chimiothérapie pour des cancers digestifs colo-rectaux et gastriques (14) et par ailleurs, la médiane de survie est significativement diminuée chez les patients ayant perdu du poids dans les 6 mois précédant la chimiothérapie. La perte de poids serait associée à un traitement plus court en raison d'arrêt pour toxicité de la chimiothérapie et à l'administration de doses plus faibles de produits, ceci pouvant expliquer partiellement une moins bonne réponse au traitement (14). Pour les cancers pulmonaires une perte de poids définie comme le pourcentage de perte entre le poids lors de la bonne santé et le poids au moment du diagnostic est un facteur très significatif de mauvaise survie lorsqu'elle est supérieure ou égale à 11% (15). La dénutrition sévère diminue significativement la médiane de survie d'un groupe de 44 enfants traités

pour LAL indépendamment de la réponse initiale à la chimiothérapie (16). La dénutrition sévère était définie par un poids inférieur d'au moins 10% en dessous d'une déviation standard du poids minimum normal pour l'âge et le sexe. La proportion de rechutes à 5 ans est augmentée dans le groupe « dénutris », expliquée par une toxicité limitante avec moindre dose-intensité.

2.1.2. PARAMETRES NUTRITIONNELS ET CHIMIOTHERAPIE

A notre connaissance, il existe un nombre limité d'études du rôle des marqueurs biologiques de la dénutrition en tant que facteur pronostique au cours de la maladie néoplasique et du rôle de la correction de la dénutrition pour améliorer le pronostic. La plupart de ces études concerne des patients opérés. Parmi les multiples paramètres biologiques et morphologiques étudiés (amaigrissement, Indice de Masse Corporelle (IMC), transthyrétine, NRI (nutritional risk index), PINI (prognostic and inflammatory nutritional index)…), l'albuminémie est le principal facteur pronostique de la survenue de complications –infectieuses et non infectieuses- ainsi que de la mortalité dans les 30 jours suivant l'intervention (17). L'intérêt d'une nutrition parentérale péri-opératoire n'est établi que chez les patients présentant une hypoalbuminémie très sévère (alb<35g/l) : les complications notamment infectieuses, sont alors diminuées d'un tiers et une mortalité hospitalière n'existait que dans le groupe contrôle de cette étude portant sur 90 sujets (18).

Les facteurs pronostiques nutritionnels sont moins bien connus au cours des traitements médicaux. Aucun marqueur « spécifique » de la dénutrition n'a pu être clairement identifié en pratique. Cependant, au-delà de toute spécificité tumorale, on constate à des degrés certes divers mais de façon relativement constante l'existence d'une anorexie centrale, associée à un dysfonctionnement hypothalamique interférant par l'intermédiaire du syndrome inflammatoire avec les systèmes sérotoninergiques ou neuropeptidiques de contrôle de la prise alimentaire. L'existence d'une interaction entre ces médiateurs inflammatoires, l'état nutritionnel, l'évolution de la tumeur voire l'impact de la chimiothérapie et la survie est probable. Ainsi chez des patients traités pour cancer bronchique, une corrélation entre le taux d'IL6 et l'extension tumorale a été retrouvée au sein d'une série de 66 patients (12), et un taux de leptine bas associé à de mauvais paramètres nutritionnels et à une moindre survie au sein d'une série de 76 patients (13). Le taux de ghréline, peptide-hormone gastrique impliquée dans la stimulation de la prise alimentaire et le stockage des lipides par les adipocytes, était significativement augmenté chez 21 patients avec cancer du poumon souffrant de cachexie cancéreuse comparé à celui retrouvé chez des patients non-cachectiques, suggérant l'apparition d'une « ghrélinorésistance » (19). Cette hypothèse était confortée par l'élévation du taux de ghréline survenant chez les sujets souffrant d'anorexie pendant la chimiothérapie dans cette même étude. L'administration de ghréline, seule hormone stimulante de l'appétit actuellement connue, à des patients cancéreux – essai randomisé sur 7 patients – ou porteurs de pathologies cardiorespiratoires (quelques dizaines de patients au total) s'est traduite par une augmentation de l'apport alimentaire (30% de l'apport énergétique), une amélioration des conditions de vie et des fonctions vitales et une diminution de l'activité sympathique (20).

2.1.3. L'INTERVENTION NUTRITIONNELLE EN CANCEROLOGIE

Au total, si l'impact de la re-nutrition chez le patient sévèrement amaigri (> 10% de perte de poids) a été clairement démontré dans la période pré et post opératoire (18), elle reste encore peu étudiée dans le temps médical de la prise en charge de la maladie cancéreuse.

Le soutien nutritionnel des patients cancéreux dénutris semble améliorer leur état nutritionnel, leur qualité de vie et leur pronostic. Les modalités de soutien décrites dans la littérature sont en majorité entérales ou parentérales et administrées à des patients ayant perdu du poids. Par exemple, le soutien nutritionnel entéral a montré son efficacité dans la réduction du taux de réhospitalisations des patients dénutris porteurs d'un cancer du haut appareil digestif (21). Cependant, dans cette dernière étude, le nombre de sujets était faible et il n'y avait pas de prise en charge diététique des patients sous nutrition orale seule. Une autre étude n'a pas montré d'efficacité d'une nutrition entérale pré opératoire administrée à des patients dénutris porteurs d'un cancer tête et cou (45 sujets, étude randomisée) (22). En soins palliatifs, un soutien nutritionnel avec recours éventuel à une nutrition parentérale n'aboutit pas à une amélioration du pronostic ou de la qualité de vie lorsque l'analyse est faite en intention de traiter (23).

En effet, le soutien nutritionnel oral préopératoire semblerait aussi efficace que le soutien périopératoire artificiel (entéral et parentéral) sur la réduction du taux des infections hospitalières et de la durée de séjour (24). Cette approche préventive a concerné d'autres traitements que les traitements chirurgicaux. Parmi 50 patients dénutris porteurs d'un cancer de l'œsophage soumis à une association de chimiothérapie et radiothérapie, les plus dénutris avaient reçu un soutien nutritionnel artificiel entéral tandis que les autres recevaient une alimentation orale spontanée pendant un an (25). Les premiers ont gardé un poids stable à la différence des derniers qui ont perdu du poids. Dans une autre étude, 600 patients adultes traités en ambulatoire par radiothérapie pour un cancer du cou ou de la tête ont été randomisés en deux groupes dont l'un a reçu un soutien nutritionnel individualisé visant à améliorer le choix et la quantité des aliments dès le début du traitement. Le groupe témoin recevait les conseils habituels du centre (plaquette

d'information et conseils généraux). Les patients ayant bénéficié de l'aide maintenaient mieux leur poids et leur qualité de vie que les témoins (26). De plus ces sujets étaient plus souvent satisfaits de la prise en charge globale de leur état de santé (27). Le soutien nutritionnel individualisé de patients porteurs de cancers gastriques ou coliques montrait un bénéfice pondéral à long terme (12 à 24 mois) comparés aux soins usuels. Ce groupe de 67 patients dans cette étude randomisée n'a malheureusement pas été décrit pour la qualité de vie et la mortalité et l'analyse n'a pas été faite en fonction du statut nutritionnel à l'inclusion (28). En revanche, une méta-analyse récente ne met pas en évidence l'intérêt de la prise en charge nutritionnelle des patients traités par radiothérapie pour un cancer pelvien (29). Cependant les études analysées visaient principalement à traiter les symptômes gastro-intestinaux liés à la radiothérapie et les seules 4 études randomisées de nutrition entérale ne montraient pas de bénéfice en terme de tolérance ni en survie. Il vient d'être montré que des patients porteurs d'un cancer de prostate et compliants à une supplémentation orale d'un produit standard avaient une prise énergétique supérieure de 500kCal/j et avaient pris du poids comparés aux non-compliants (30).

La réponse à la chimiothérapie a été étudiée chez des patients porteurs de cancer du poumon ou de cancer colo-rectal selon qu'ils recevaient ou non des conseils diététiques visant à augmenter leur apport calorique de 25 %. Il n'a pas été constaté de différence de réponse à la chimiothérapie entre les deux groupes mais ceux-ci n'étaient pas décrits sur le plan nutritionnel (31).

Les recommandations de la société européenne de nutrition entérale et parentérale (ESPEN) parues en 2006, proposent une intervention nutritionnelle par nutrition orale ou artificielle, de préférence entérale, avant le début d'un traitement par chimiothérapie de manière à atteindre un apport calorique de 30-35 kCal/kg/j pour des personnes autonomes et de 20-25 kCal/kg/j pour les alités (32). Les apports protidiques recommandés sont d'un minimum de 1 g/kg/j avec pour objectif d'atteindre 1,2 g/kg/j. Il est conseillé de suivre les recommandations générales pour les vitamines et les minéraux mais d'être attentif aux apports en anti-oxydants, le statut antioxydant plasmatique étant altéré chez les sujets cancéreux. Dans ces recommandations, il n'est pas fait mention de l'âge mais les apports conseillés répondant à ceux utilisés chez la personne âgée (33).

Cependant en pratique, le recours au soutien nutritionnel des patients cancéreux de tous âges ne semble pas être systématique même lorsque les informations nutritionnelles sont disponibles. Une étude portant sur plus de 900 patients consultant pour un cancer gastro-intestinal dans un centre de traitement ambulatoire disposant d'une aide diététique dévolue à cette activité a montré que seuls 36 % d'entre eux en bénéficiaient (34). La probabilité de ce recours augmentait avec le temps. Elle était supérieure pour ceux ayant perdu plus de 10% de leur poids (56% à 12 mois) et était la même pour les patients sans perte de poids ou avec 5-10% de perte de poids (40 % à 12 mois).

2.1.4. MALNUTRITION ET SUJET AGE

La malnutrition protéino-énergétique survient fréquemment chez la personne âgée pour de multiples raisons et en particulier en raison de la prévalence élevée des pathologies chroniques cachectisantes dans cette tranche d'âge. Il n'y a pas d'étude montrant l'efficacité du soutien nutritionnel artificiel entéral ou parenteral mais les indications sont le plus souvent les problèmes d'alimentation liés aux Accidents Vasculaires Cérébraux (AVC), aux démences et aux maladies neurodégénératives (35, 36). Pour les démences sévères, la nutrition entérale au long cours s'est révélée être, en fait, un facteur pronostique indépendant de mortalité (37). Dans une étude portant sur 150 patients consécutifs dont 13 % avaient un cancer, aucun bénéfice sur la survie n'était associé avec la nutrition entérale au long cours chez les patients âgés (38).

Ces résultats peu encourageants concernant la nutrition entérale ont conduit les praticiens à privilégier le soutien nutritionnel oral dans la population gériatrique. Un peu plus de la moitié des personnes âgées accepte la prise de tout ou partie de la supplémentation orale par des produits industriels lors d'une hospitalisation (39-41). Cette supplémentation orale améliore la prise énergétique et surtout protidique (39, 42). Une supplémentation orale (540 kcal, 22,5 g protides par jour) proposée à la moitié de 381 patients hospitalisés pour une pathologie aiguë a montré qu'un gain de poids et une amélioration fonctionnelle pouvait être observée chez les plus dénutris (42). L'efficacité de cette supplémentation sur les affections liées à la pathologie aiguë est plus délicate à mettre en évidence. Pour une population de plus de 70 ans hospitalisée pour une affection immobilisante, cette supplémentation était associée à une diminution du risque d'escarre de stade I (672 sujets) (39) mais d'autres études de plus faible puissance (environ 100 sujets pour chacune d'elles) n'ont pas montré d'effet) (41, 43, 44).

A domicile, l'adhésion semble être supérieure à celle observée chez le patient hospitalisé. Une supplémentation orale (de type industrielle : 300 à 500 kCal, 10 à 20 g de protéines) administrée pendant 3 mois à domicile à des sujets âgés présentant une démence et un risque nutritionnel a amélioré la prise alimentaire d'environ 5 kCal/kg et 0,12 g protéine/kg de poids (45). Ceci s'accompagnait d'une prise de poids et d'un gain de masse maigre sans toutefois provoquer d'amélioration fonctionnelle. Le MNA (Mini Nutritional Assessment) était utilisé pour identifier les sujets dénutris et à risque de dénutrition (46). Cette

échelle dont le score maximal est de 30 décrit des paramètres anthropométriques, fonctionnels, mentaux, le comportement alimentaire, la comorbidité et la polymédication. Un score inférieur ou égal à 17 traduit une dénutrition et un score entre 17,5 et 23,5 identifie les sujets à risque de dénutrition. Dans cette dernière étude (45) les sujets avaient un score inférieur à 23,5 et le gain de MNA était de 3,4+/-3,1 dans le groupe d'intervention contre 1.9+/-3.5 chez les témoins. Cependant le nombre de sujets était limité (37 dans le groupe intervention, 43 dans le groupe témoin). Les patients devant recevoir une chimiothérapie étaient par ailleurs exclus.

Ces études ne différenciaient pas clairement les sujets dénutris et ceux à risque de dénutrition. Pour un groupe réduit (66 sujets institutionnalisés) une supplémentation orale administrée à des sujets à risque de dénutrition (MNA 17-23,5) diminuait la prise alimentaire normale pour une prise quotidienne totale inchangée tandis que les dénutris (MNA< 17) augmentaient significativement leurs apports (47). Ceci justifie la mesure des ingestas totaux avant et après supplémentation.

L'utilisation de suppléments industriels ou préparés sur place n'est pas le seul moyen d'améliorer la prise alimentaire. L'aide humaine, pour le choix, la quantité et l'aide à la prise alimentaire elle-même peut-être testée. Cependant, une étude de l'efficacité de cette aide humaine portant sur près de 600 patients de médecine interne de plus de 65 ans, mais non sélectionnés et non décrits pour leur statut nutritionnel, n'a pas montré d'efficacité qualitative et quantitative sur la prise alimentaire à l'hôpital (48).

2.2. HYPOTHESES DE L'ETUDE

Depuis 2000, des consultations d'oncogériatrie ont été mises en place progressivement dans 10 villes d'Aquitaine (INACAN) afin d'évaluer de manière prospective l'état de santé global des patients de plus de 70 ans recevant une chimiothérapie pour cancer ou lymphome. Trois cent soixante patients ont été sélectionnés par les oncologues des centres hospitaliers publics et/ou privés et évalués selon le protocole classique de l'Evaluation Gérontologique Standardisée (EGS) par un gériatre et un infirmier. Les évaluations étaient effectuées avant le premier, le deuxième et le quatrième cycle de chimiothérapie puis à la fin du traitement (3 – 4 mois) (49). L'EGS permet de compléter l'examen clinique par l'étude exhaustive de l'état fonctionnel, cognitif, thymique, nutritionnel ainsi que la co-morbidité, la continence, la marche et le risque de chutes, les traitements et les conditions de vie. L'EGS repose sur des échelles et des tests ainsi que sur une synthèse médico-sociale. Elle apporte une aide à la résolution des problèmes gérontologiques complexes en dirigeant les interventions thérapeutiques médicales, psychologiques, et sociales sur les résultats de l'évaluation (50). Une telle approche a montré son efficacité en améliorant l'autonomie des patients instables, en diminuant le risque d'hospitalisation et en diminuant le risque de syndrome confusionnel (51). En oncologie, l'EGS associée avec la prise en charge du cancer peut offrir les moyens d'une prévention optimale de la perte d'autonomie et maintenir la qualité de vie (52). De plus une intervention précoce, basée sur l'EGS et pratiquée par une équipe infirmière à domicile après chirurgie a permis d'augmenter la survie de patients âgés cancéreux comparée à celle de ceux recevant des soins usuels (53). Aussi cette prise en charge basée sur l'EGS des patients âgés cancéreux est maintenant recommandée par la Société Internationale d'Oncogériatrie (SIOG) (54).

L'analyse intermédiaire des résultats des 155 premiers patients inclus d'INACAN (dont 150 ont eu un MNA), a montré qu'à l'évaluation gérontologique initiale, 28 (18,7 %) étaient dénutris (MNA<17) et 72 (48,0 %) à risque de dénutrition (MNA 17-23,5). Cette proportion de patients dénutris ou à risque de dénutrition est à rapprocher des 65 % retrouvés par une autre équipe d'oncogériatrie française (52). A la fin du suivi des patients d'INACAN dont la médiane était de 1 an, la mortalité était d'autant plus importante que l'atteinte nutritionnelle était initialement grave : 20 (71,4%) des dénutris étaient décédés, 34 (47,2 %) des patients à risque de dénutrition et 13 (26,0 %) des 50 sujets non identifiés comme ayant des problèmes nutritionnels. La mortalité précoce (avant 3 mois) suivait également cette tendance : 11 des 28 dénutris, 10 des 72 sujets à risque et 2 des 50 « indemnes ». Cette mortalité variable pourrait concerner certains cancers, ayant d'une part un mauvais pronostic et étant d'autre part souvent associé à une dégradation de l'état nutritionnel. Il est à noter cependant que parmi les patients d'INACAN, comportant 30% de lymphome et 30 % de cancers du colon, aucune différence significative de l'état nutritionnel n'était constatée entre cancers digestifs et non-digestifs.

Tableau 1. Mortalité en fonction de l'état nutritionnel chez 150 patients âgés atteints de cancer. INACAN (coordonnateur : P Soubeyran, données non publiées)

	MNA < 17<br>n=28 (19 %)	MNA 17–23,5<br>n=72 (48,0%)	MNA ≥ 24<br>n=50 (33,3%)
Mortalité : n (%)			
Avant 3 mois	11 (39,3)	10 (13,9)	2 (4,0)
Suivi 1 an	20 (71,4)	34 (47,2)	13 (26,0)

Les études de soutien nutritionnel des patients porteurs de cancer n'ont pas concerné spécifiquement les sujets de plus de 65 ans et leur proportion dans l'ensemble des patients n'était pas décrite. Néanmoins quelques essais prospectifs encore de petite taille ont été publiés au cours de ces dernières années, évaluant dans le cadre de l'Evaluation Gérontologique Globale (EGS) la tolérance à la chimiothérapie. Dans ces séries, plus de la moitié des patients présentaient une dégradation de leur état nutritionnel en cours de traitement, avec un retentissement sur leur qualité de vie. Ces modifications n'étaient pas corrélées de façon significative à la survenue de toxicité limitante de la chimiothérapie (55, 56).

Parmi les 50 patients atteints de cancer de l'œsophage et traités par chimiothérapie et radiothérapie de l'étude de Bozzetti et coll (25), 29 sujets avaient reçu un soutien nutritionnel entéral et 21 un soutien oral, les 29 premiers présentant un amaigrissement plus important. La survie était cependant similaire chez les deux groupes de patients, suggérant l'efficacité du soutien des plus dénutris. Chez 67 patients atteints de cancers colo-rectaux ou gastriques, l'intervention nutritionnelle orale était associée à une amélioration non statistiquement significative de la survie (28).

Nous faisons l'hypothèse que le soutien nutritionnel des sujets à risque de dénutrition et détectés lors de l'EGS pourrait améliorer leur état nutritionnel ou freiner sa dégradation pendant le traitement. Cette préservation de l'état nutritionnel pourrait améliorer la survie à court, moyen, et long terme, la tolérance du traitement ainsi que l'état fonctionnel et la qualité de vie des patients.

L'objectif de l'étude interventionnelle que nous proposons est d'évaluer l'impact d'une intervention visant à améliorer l'état nutritionnel des sujets âgés, à risque de dénutrition pendant leur traitement oncologique, et par là même à améliorer leur survie et leur tolérance à la chimiothérapie. Un suivi biologique de paramètres nutritionnels et généraux sera effectué au cours de l'étude à la recherche de déterminants biologiques de la tolérance au traitement et éventuellement de la survie. Une numération formule sanguine, un dosage de l'albuminémie et de la CRP seront effectués en début et fin d'étude. Une sérothéque sera constituée en vue de l'étude ultérieure des facteurs neuro-hormonaux et immunitaires associés à la dénutrition du patient cancéreux âgé.

Nous proposons donc de recruter parmi les patients âgés devant bénéficier d'une chimiothérapie, un groupe de sujets à risque de dénutrition selon le MNA qui sera pratiqué spécifiquement pour cette étude. Pour recruter le nombre de sujets à risque de dénutrition voulu (**820**), nous pensons devoir faire passer le MNA à **1640** sujets.

Si lors de la réalisation de ce MNA, des patients dénutris sont repérés, ils seront systématiquement dirigés vers le soutien nutritionnel habituel proposé à ce type de patients, confiés aux équipes d'oncologie. Ces sujets ne seront pas suivis dans le cadre de cette étude, ainsi que les patients non-dénutris.

Les patients à risque de dénutrition seront randomisés en deux groupes : le groupe « soins usuels et intervention nutritionnelle spécifique» recevra un soutien nutritionnel individualisé tandis que le groupe « soins usuels » bénéficiera de la prise en charge habituelle de l'équipe d'oncologie informée des résultats de l'EGS. Cette étude originale est pragmatique.

2.3. SOUTIEN NUTRITIONNEL SPECIFIQUE A L'ETUDE

Un entretien avec la diététicienne de l'étude dès l'inclusion visera à assurer un apport de 30 kCal/kg/j ou plus et 1,2 g protides/kg/j. La diététicienne devra mettre en œuvre les moyens adaptés à l'autonomie (Activités élémentaires de la vie quotidienne : ADL et Activités instrumentales de la vie quotidienne : IADL) de la personne à aider. Pour cela l'équipe de gériatrie apportera son aide. La pertinence des conseils donnés sera vérifiée par un appel téléphonique la semaine qui suit la première visite. Il sera possible d'utiliser des suppléments (industriels ou non) si nécessaire ou encore de mettre en place une nutrition artificielle si celle-ci est formellement indiquée pour atteindre les objectifs. Les sujets seront revus par la

diététicienne à chaque cycle de chimiothérapie. L'adhésion à l'intervention sera évaluée par enquête alimentaire.

2.4. JUSTIFICATION DES CHOIX METHODOLOGIQUES

Les patients atteints d'un cancer et dénutris semblent, selon la littérature, bénéficier du soutien nutritionnel et dans l'état actuel des connaissances (57), il ne paraît pas éthique de randomiser un soutien nutritionnel. Le bénéfice que les sujets à risque de dénutrition peuvent espérer de ce soutien, est lui non évalué. Pourtant leur taux de mortalité est intermédiaire entre celui des dénutris et celui des non-dénutris. Enfin, le soutien nutritionnel de patients non-dénutris n'a pas de sens. Pour cette raison, nous n'inclurons dans l'étude que les patients à risque de dénutrition pour leur proposer une intervention randomisée.

3. OBJECTIFS

3.1. OBJECTIF PRINCIPAL

L'objectif principal est d'évaluer les effets d'un soutien nutritionnel individualisé par rapport à la prise en charge habituelle sur la survie à 1 an des patients âgés (≥ 70 ans) à risque de dénutrition selon le MNA pendant leur traitement oncologique.

3.2. OBJECTIFS SECONDAIRES

- Les objectifs secondaires sont d'évaluer les effets de ce soutien nutritionnel appliqué à cette population de sujets à risque de dénutrition sur :
  - la survie à 2 ans,
  - la tolérance de la chimiothérapie (fréquence des toxicités sévères ou de grade 3-4),
  - l'état fonctionnel (maintien ou amélioration) en fin de traitement par chimiothérapie
  - la qualité de vie en fin de traitement par chimiothérapie
  - l'état nutritionnel en fin de traitement par chimiothérapie

et d'étudier :
  - l'observance,
  - les déterminants cliniques et biologiques de la tolérance de la chimiothérapie et de la survie.

4. SCHEMA ET RANDOMISATION

4.1. SCHEMA / METHODES

Il s'agit d'un essai clinique comparatif randomisé de supériorité, sans insu et multicentrique en 2 groupes parallèles :

- **groupe « soins usuels »** : soins usuels (prise en charge habituelle de l'équipe d'oncologie) et pas de soutien nutritionnel spécifique à l'étude
- **groupe « soins usuels + intervention nutritionnelle spécifique »** : soutien nutritionnel individualisé (réalisé par une diététicienne, dès la première visite et pendant toute la durée de la chimiothérapie, visant à assurer un apport de 30 kCal/kg/j ou plus et 1,2 g protides/kg/j)

4.2. ECHEANCIER DE L'ESSAI

- Période d'inclusion : 24 mois
- Durée de participation pour chaque patient : 24 mois dont la durée initiale de la chimiothérapie avec l'évaluation complète en fin de chimiothérapie (3 à 4 mois ou 6 cycles), puis le suivi de la survie par contact téléphonique (à 12 mois et jusqu'à 2 ans).
- Durée totale de l'essai : 4 ans

Il est prévu que l'essai débute au premier trimestre 2007.

4.3. Randomisation

La liste de randomisation est établie par le statisticien du Centre de Méthodologie et de Gestion avant le début de l'essai. Les effectifs des 2 groupes d'intervention sont équilibrés avec un ratio de 1:1. La randomisation est stratifiée sur le centre. Un document décrivant la procédure de randomisation est conservé de manière confidentielle au sein du Centre de Méthodologie et de Gestion.

La randomisation des patients est effectuée de façon centralisée *via* un site Internet dédié à l'étude. Cette centralisation permet de contrôler l'éligibilité des patients et de communiquer pour chacun d'entre eux les informations relatives à la randomisation à l'investigateur et d'autres correspondants éventuellement.

La procédure à suivre pour la randomisation d'un patient est présentée dans le paragraphe §9.4 du protocole.

5. CRITERES D'ÉLIGIBILITE

Les critères d'éligibilité, sont vérifiés lors de la visite de pré-inclusion (Visite 0) qui a lieu le même jour que la visite d'inclusion (Visite 1) et que le début de la prise en charge nutritionnelle randomisée ; c'est aussi le début du traitement par chimiothérapie.

Le consentement libre, éclairé et écrit du patient est recueilli au plus tard, lors de la visite d'inclusion et avant tout examen nécessité par l'essai.

5.1. Criteres de pre inclusion

- Adultes des 2 sexes âgés de ≥ 70 ans à la visite de pré-inclusion
- Lymphome ou carcinome histologiquement prouvé
- cancer du colon en indication de chimiothérapie *(adjuvante ou palliative)*
- cancer du sein en indication de chimiothérapie
- cancer de l'estomac en indication de chimiothérapie *(d'induction ou palliative)*
- cancer du pancréas en indication de chimiothérapie
- cancer de l'ovaire en indication de chimiothérapie *(adjuvante ou palliative)*
- tous lymphomes diffus à grandes cellules B, tous lymphomes T périphériques
- tous lymphomes de faible malignité : lymphocytiques, lympho-plasmocytaires, folliculaires, manteau, zone marginale (MALT et autres)
- Cancer de la prostate en indication de chimiothérapie
- Cancer de la vessie en indication de chimiothérapie
- Cancer bronchique en indication de chimiothérapie
- Adénocarcinome de primitif inconnu compatible avec une origine citée ci-dessus en indication de chimiothérapie.
- Cancer relevant d'un traitement par chimiothérapie jusqu'à la troisième ligne de chimiothérapie selon un protocole selon un protocole conforme aux accords de bon usage établis au sein de chaque établissement. La liste de ces protocoles sera établie au sein de chaque établissement avant le démarrage de l'étude et mise à jour tous les 3 mois
- Espérance de vie > 12 semaines, l'Index de Karnofsky doit être supérieur ou égal à 50%
- MNA compris entre 17 et 23,5 : patients à risque de dénutrition.
- Patient affilié ou bénéficiaire d'un régime de sécurité sociale.

5.2. Criteres d'inclusion

- Critères de pré inclusion,

- Consentement libre, éclairé et écrit signé par le patient et l'investigateur,

5.3. CRITERES DE NON INCLUSION

- MNA < 17 ou ≥ 24
- Impossibilité de suivi pour raison géographique, psychologique ou sociale : les sujets dont le traitement de chimiothérapie se ferait dans un centre ne permettant ni l'intervention de la diététicienne, ni les évaluations gérontologiques de début et de fin d'essai.
- Métastases cérébrales ou méningées symptomatiques,
- Patients sous protocoles de chimiothérapie non publiés sous forme d'article,
- Posologie et schéma de traitement non compatibles avec le suivi, comme l'absence de visite entre 3 et 4 mois et ne permettant donc pas les évaluations gérontologiques de début et de fin de chimiothérapie, ni l'intervention nutritionnelle spécifique
- Personnes placées sous sauvegarde de justice,

6. INTERVENTIONS COMPAREES

Les interventions comparées nécessitent une standardisation à priori.

A cette fin :

- Dans le groupe « soins usuels »

  Avant le démarrage de l'étude, les bonnes pratiques propres à chaque centre seront recueillies. Il sera recommandé de suivre ces bonnes pratiques.

- Dans le groupe « soins usuels plus intervention nutritionnelle spécifique »

  Une formation sera organisée par la diététicienne coordinatrice de l'étude et sera dispensée dans chacun des centres participants afin de standardiser l'intervention nutritionnelle spécifique à l'étude.

Après signature du consentement éclairé et vérification des critères d'éligibilité, les patients sont randomisés sans insu dans l'un des groupes suivants :

6.1. GROUPE SOINS USUELS

La prise en charge correspond à celle habituellement effectuée dans le service.

Le recours à une aide diététique selon les procédures habituelles du centre est autorisé mais cette aide doit être apportée par une diététicienne différente de celle qui intervient dans l'autre groupe (groupe soins usuels plus intervention spécifique à l'étude). En effet, la diététicienne qui prendra en charge les patients du groupe «« soins usuels plus intervention nutritionnelle spécifique», ne doit ni voir les patients du groupe « soins usuels », ni donner des conseils pour ces patients aux prescripteurs.

Les prises alimentaires effectives seront évaluées par les auto-questionnaires diététiques lors de la visite 1, à chaque visite de cycle jusqu'à la visite FC.

6.2. GROUPE SOINS USUELS PLUS INTERVENTION NUTRITIONNELLE SPECIFIQUE

L'intervention nutritionnelle spécifique débute dès la première visite (Visite 1) et se poursuit pendant toute la durée de la chimiothérapie. Elle est effectuée par une diététicienne différente de celle qui intervient dans le groupe « soins usuels ». Elle ne doit ni voir les patients du groupe « soins usuels », ni donner des conseils pour ces patients aux prescripteurs

L'entretien avec la diététicienne de l'étude dès l'inclusion vise à assurer un apport de 30 kCal/kg/j ou plus et 1,2 g protides/kg/j.

La diététicienne de l'étude doit mettre en œuvre les moyens adaptés à l'autonomie (ADL et IADL) de la personne à aider. Pour cela l'équipe de gériatrie apporte son aide. La pertinence des conseils diététiques donnés est vérifiée par un appel téléphonique la semaine qui suit la première visite.

Les sujets seront revus par la diététicienne à chaque cycle de chimiothérapie si ceux ci permettent une visite tous les 15 jours. Si les cycles de chimiothérapies sont espacés de 3 semaines, un entretien

téléphonique aura lieu entre les deux visites. S'ils sont espacés d'un mois, le patient reviendra au bout de 15 jours pour rencontrer la diététicienne. L'observance à l'intervention est évaluée par enquête alimentaire.

En pratique, la diététicienne de l'étude voit les patients à chaque cycle de chimiothérapie, assure un suivi téléphonique si nécessaire, donne ses conseils et s'adapte aux problèmes nutritionnels du patient jusqu'à la visite de fin de chimiothérapie (visite FC).

Les enquêtes diététiques seront recueillies par l'investigateur à chaque visite de V-1 à V-FC selon un auto-questionnaire standardisé, puis interprétées par la diététicienne selon les mêmes modalités que pour le groupe « soins usuels ».

7. TRAITEMENTS ASSOCIES

La chirurgie et la radiothérapie sont autorisées ainsi que tout traitement associé jugé nécessaire à la prise en charge du patient.

Les oncologues sont libres de prodiguer des conseils et de prescrire des suppléments oraux (industriels ou non) s'ils les jugent nécessaires en fonction de l'évolution de l'état du patient au cours du suivi ou encore de mettre en place une nutrition artificielle, si celle-ci est formellement indiquée pour atteindre les objectifs.

Les recommandations de l'ESPEN en matière de nutrition artificielle pour les patients cancéreux traités non chirurgicalement seront suivies (32). Dans ce cas, les indications et le suivi de cette nutrition artificielle seront documentés auprès de l'ARC coordonnateur et dans le cahier d'observation.

8. CRITERES DE JUGEMENT

8.1. CRITERE DE JUGEMENT PRINCIPAL

Le critère de jugement principal est la survie un an après la randomisation (début de l'intervention nutritionnelle).

Le recueil du statut vital se fera auprès du médecin traitant auquel on demandera de signaler tout décès du patient dans les 15 jours. Il est prévu également un contact tous les 6 mois avec le patient ou sa famille pour recueillir le statut vital et anticiper les changements d'adresse.

Toute source d'information potentielle sera utilisée pour connaître le statut vital si le patient est perdu de vue avant la fin de la première année, notamment le registre du cancer et le RNIPP (Répertoire National d'Identification des Personnes Physiques).

8.2. CRITERES DE JUGEMENT SECONDAIRES

Les critères de jugement secondaires sont :

- Survie à 2 ans,
- Tolérance de la chimiothérapie : évaluée par l'échelle des grades de toxicité selon la NCI CTCv3 à chaque visite de chimiothérapie,
- Thymie (GDS) (à chaque visite de V 1 et V FC),
- Fonction : IADL, ADL, get up and go test, appui monopodal (V1 et V FC),
- Qualité de vie (QLQ–C30) (V 1 et V FC),
- MNA (V 1 et V FC),
- Poids (à chaque visite de V 1 à V FC)
- Biologie : CRP, albumine, hémoglobine (V 1 et V FC),
- Enquêtes alimentaires (à chaque visite de V 1 à V FC) pour évaluer l'observance :
    - Méthode du rappel des 24 heures en auto-questionnaire avec éventuellement aide d'un accompagnant,
    - Nombre et type de suppléments consommés,
    - Alimentation artificielle (type, quantité, qualité, durée, complications),
- Hospitalisations (nombre de jours),

- Infections de traduction clinique générale (fièvre, dysurie, dyspnée, nécessité d'antibiothérapie),
- Mortalité pendant la phase de chimiothérapie, et à deux ans
- Autres : fractures, chutes, escarres.

9. DEROULEMENT DE L'ESSAI

9.1. MODALITES DE RECUEIL DU CONSENTEMENT

Lors de la visite de pré inclusion (visite V0), le médecin investigateur informe le patient et son représentant légal (personne de confiance nommément désignée ou membre de la famille) et répond à toutes leurs questions concernant l'objectif, la nature des contraintes, les risques prévisibles et les bénéfices attendus de l'étude. Il précise également les droits du patient dans le cadre d'une recherche biomédicale.

Il précise également au patient, et à son représentant légal, avant la réalisation du MNA, que si le patient est identifié comme à risque de dénutrition (MNA entre 17 et 23,5), il pourra, s'il donne son consentement, être inclus dans l'étude. S'il est identifié comme dénutri (MNA < 17), il sera dirigé vers une prise en charge hors étude. Comme les sujets non dénutris, sa participation à l'étude s'arrêtera là.

Avant la réalisation de tout examen clinique ou para clinique nécessité par l'étude, lors de la visite d'inclusion (visite V1), la note d'information tripliquée et le formulaire de consentement tripliqué sont alors remis au patient ou au représentant légal (cf. annexe 1) par le médecin investigateur, responsable de l'obtention du consentement éclairé écrit du patient ou du représentant légal.

Les visites V0 et V1 peuvent se succéder le même jour.

Ces dispositions sont prises en conformité avec les articles L 1122-1 et L1122-2 du Code de la Santé Publique.

Si le patient, ou son représentant légal, donne son accord de participation, ce dernier et l'investigateur inscrivent leurs noms et prénoms en clair, datent et signent le formulaire de consentement.

Les différents exemplaires de la note d'information et du formulaire de consentement sont alors repartis comme suit :

- un exemplaire du consentement signé est remis au patient ou à son représentant légal,
- l'exemplaire original est conservé par le médecin investigateur (même en cas de déménagement du patient pendant la durée de l'essai) dans un lieu sûr inaccessible à des tiers, pour une durée de 30 ans après la fin de l'essai,
- à la fin des inclusions ou au plus tard à la fin de l'essai, un exemplaire de chaque formulaire de consentement est transmis au promoteur ou à son représentant selon des modalités communiquées en temps utile aux investigateurs.

Tout amendement qui modifie la prise en charge des patients fait l'objet d'une nouvelle note d'information et d'un nouveau formulaire de consentement dont le recueil suit la même procédure que celle pré-citée.

9.2. TABLEAU RECAPITULATIF DU SUIVI PATIENT

	Pré-inclusion	Inclusion	Visites de suivi				
	Visite 0	Visite 1 (1er cycle de chimiothérapie)	Visite 2 (2ème cycle de chimiothérapie)	…	Visite N (Nième cycle de chimiothérapie)	Visite FC (fin de chimiothérapie)	Visite 1 et 2ans Téléphone ou courrier
Information	X						
Consentement éclairé		X					
MNA	X					X	
Evaluation gérontologique[1]		X				X	
Poids		X	X	X	X	X	
Enquête diététique		X	X	X	X	X	
Evaluation oncologique[2]		X	X	X	X	X	
Bilan biologique[3]		X				X	
Sérothèque[4]		X				X	
Statut vital		X	X	X	X	X	X

[1] Automonie physique (ADL, IADL risque de chute évalué par version chronométrée du Get Up and Go test, recueil de nombre de chutes dans les 3 mois précedents), fonctions cognitives (par MMS), humeur (par GDS), qualité de vie (QLQ-C30), enquête alimentaire par la méthode de rappels des 24 heures ;
[2] Description initiale du cancer, recueil des co-morbidités à l'inclusion et évaluation de la tolérance de la chimiothérapie à chaque cycle détaillé dans le paragraphe correspondant
[3] Bilan biologique détaillé dans le paragraphe correspondant
[4] Conditionnée au niveau de chaque centre

9.3. VISITE DE PRE INCLUSION (VISITE 0)

La visite de pré inclusion, qui peut être contemporaine de la visite d'inclusion, est assurée par le médecin investigateur.

Au cours de cette visite, l'investigateur :

- Informe le patient
- réalise le MNA
- vérifie les critères d'éligibilité
- classe le patient en dénutris, à risque de dénutrition ou non-dénutri

Tous les patients qui auront passé un MNA seront enregistrés.

9.4. VISITE D'INCLUSION (VISITE 1)

La visite d'inclusion est assurée par le médecin investigateur, oncologue ou gériatre. Celui-ci évalue le patient en collaboration avec, selon l'appartenance du médecin investigateur, l'équipe d'onco-gériatrie et l'oncologue.
Cette visite correspond au premier jour de traitement de chimiothérapie.

9.4.1. RECUEIL DU CONSENTEMENT

Au cours de cette visite, le médecin investigateur recueille le consentement libre, éclairé et écrit du patient si son MNA est compris entre 17 et 23,5

- MNA < 17 : *patients dénutris*
- MNA compris entre 17 et 23,5 : *patients à risque de dénutrition*
- MNA ≥ 24 : *patients sans problèmes nutritionnels ou non-dénutris*

9.4.2. RANDOMISATION

Si tous les critères d'éligibilité sont respectés, l'investigateur se connecte sur le site Internet de l'USMR : http://usmr.isped.u-bordeaux2.fr . L'investigateur complète la page web « randomisation » après avoir préalablement confirmé tous les critères d'éligibilité du patient sur le site. Il valide le contenu ; l'inclusion est effectuée et le site communique immédiatement le résultat de la randomisation à

l'investigateur, en particulier le groupe d'intervention alloué au patient et le numéro unique du patient dans l'essai.

En cas d'impossibilité de se connecter au site Internet, l'investigateur contactera le Centre de Méthodologie et de Gestion par téléphone ou par fax pour réaliser la randomisation.

9.4.3. DEROULEMENT DE LA VISITE

- Les patients âgés seront évalués par l'équipe de gériatrie (Evaluation gérontologique) au moment de la mise en route de la chimiothérapie.
  Ces évaluations comportent une série de tests mesurant :
  - l'autonomie physique : ADL (activités élémentaires de la vie quotidienne) (58), (IADL (activités instrumentales de la vie quotidienne) (59), le risque de chute par l'appui monopodal et la version chronométrée du Get Up and Go test (60) et le recueil du nombre de chutes les trois mois précédents,
  - les fonctions cognitives par le Mini Mental State (MMS) de Folstein (61),
  - l'humeur par la Geriatric Depression Scale (GDS) (62),
  - l'état nutritionnel par le Mini Nutritionnal Assessment (MNA) qui aura déjà été fait (46), le poids, et une enquête alimentaire par la méthode du rappel des 24 heures (auto-questionnaire) (45). La perte de poids devra être particulièrement documentée : poids antérieur il a 1 mois, 3 mois et 6 mois. L'impossibilité de retrouver cette information sera considérée comme une donnée à analyser.
  - les comorbidités (Cumulative Illness Rating Scale for Geriatrics [CIRS–G]) (63),
  - la qualité de vie par le questionnaire QLQ–C30 (64) ,

  Cette consultation est réalisée par un binôme infirmier et médecin :
  - infirmier : questionnaires ADL, IADL, et aide aux auto–questionnaires (GDS, QLQ–C30)
  - médecin : Get up and Go test, MMS, MNA, CIRS–G

  Sa durée totale excède rarement 40 minutes.
- Les patients seront tous évalués par l'oncologue qui :
  - décrit la tumeur, la date de son diagnostic, les thérapeutiques antérieures
  - prescrit les examens biologiques suivants : CRP, albumine, hémoglobine, créatininémie
- L'infirmier organise la réalisation du bilan biologique : CRP, albumine, hémoglobine, créatininémie et 2 tubes secs pour la sérothèque.
  La sérothèque sera gérée par chaque institut.

9.5. VISITES DE SUIVI

Tous les patients randomisés doivent être suivis, selon le calendrier défini, jusqu'à la fin de l'essai, même s'ils n'ont jamais bénéficié de l'intervention nutritionnelle ou s'ils l'interrompent.

Les visites auront lieu au début de chaque cycle de chimiothérapie.

Les visites sont numérotées de 2 à N, selon le nombre de cycle de chimiothérapie administré.

La visite à chaque cycle de chimiothérapie, est réalisée par l'oncologue

L'oncologue investigateur recueille à chaque visite :

- les données de tolérance de la chimiothérapie selon les grades de toxicité de la NCI CTCv3 (Annexe 6).
- L'auto-questionnaire diététique
- Les évènements intercurrents (hospitalisation, infections, escarres, traumatisme)
- Le poids

Il renseigne le statut vital du sujet.

A l'occasion de ces visites, un entretien avec la diététicienne sera organisé pour les patients dans le groupe « Soins usuels + Intervention Nutritionnelle spécifique à l'étude» pour la poursuite de la prise en charge. Néanmoins, les patients rencontreront la diététicienne à chaque cycle de chimiothérapie si ceux ci permettent une visite tous les 15 jours. Si les cycles de chimiothérapies sont espacés de 3 semaines, un entretien téléphonique aura lieu entre les deux visites. S'ils sont espacés d'un mois, le patient reviendra au bout de 15 jours pour rencontrer la diététicienne.

Les auto-questionnaires diététiques seront interprétés secondairement à l'aide du logiciel Bilnut par la diététicienne (Tours, tables de composition alimentaire).

9.6. VISITES FC (FIN DE CHIMIOTHERAPIE)

Le patient bénéficie :

- d'une évaluation gérontologique comportant comme à la visite initiale :
  - l'autonomie physique : ADL, IADL, le risque de chute par l'appui monopodal et la version chronométrée du Get Up and Go test et le recueil du nombre de chutes les trois mois précédents
  - les fonctions cognitives par le Mini Mental State (MMS) de Folstein
  - l'humeur par la Geriatric Depression Scale (GDS)
  - l'état nutritionnel par le Mini Nutritionnal Assessment (MNA), le poids et l'auto-questionnaire diététique
  - les comorbidités (Cumulative Illness Rating Scale for Geriatrics [CIRS–G])
  - la qualité de vie par le questionnaire QLQ–C30
- d'une évaluation oncologique
  L'oncologue investigateur recueille comme à chaque visite
  - les données de tolérance de la chimiothérapie selon les grades de toxicité de la NCI-CTCv3
- d'un bilan biologique réalisé par l'infirmier : CRP, albumine, hémoglobine, créatininémie et tubes secs pour la sérothèque.

9.7. VISITES A 1 ET 2 ANS

Le statut vital de chaque patient sera recherché pendant les deux ans qui suivent son inclusion.

Le médecin traitant du patient est informé de l'inclusion de son patient dans l'essai et il lui est demandé de notifier au médecin investigateur, dès qu'il en a connaissance, le décès éventuel du patient.

Sans information concernant le statut vital d'un patient, il est prévu de contacter (par courrier/téléphone) tous les 6 mois le patient et le cas échéant le médecin traitant et la famille. Ces contacts réguliers permettront également d'anticiper les changements d'adresse éventuels

En cas de décès, l'investigateur renseignera la date de celui-ci.

9.8. CONDUITE A TENIR EN CAS DE NUTRITION ARTIFICIELLE

Si une nutrition artificielle est mise en place (indication formelle selon les recommandations en vigueur), l'investigateur doit le notifier aussi vite que possible, par fax, à l'ARC coordonnateur de l'étude à l'aide du formulaire disponible dans le cahier d'observation.

S'agissant d'un événement majeur d'intérêt dans le contexte de l'étude, il est important que cet événement soit soigneusement documenté dans le cahier d'observation.

9.9. DEVIATIONS AU PROTOCOLE

9.9.1. ARRET PREMATURE ET DEFINITIF DE LA STRATEGIE D'INTERVENTION DE L'ESSAI

Un patient est considéré en arrêt de la stratégie d'intervention quand il refuse les visites avec la diététicienne de l'essai mais qu'il continue le suivi prévu dans le cadre du protocole (visites, prélèvements, examens complémentaires…).

En dehors des décès, un arrêt prématuré est considéré comme une déviation au protocole.

Les arrêts prématurés de stratégie de l'étude doivent être notifiés rapidement à l'ARC coordonnateur, par fax via la fiche correspondante du classeur d'observation.

Les raisons ainsi que la date de cet arrêt prématuré doivent être documentées.

Le patient qui arrête la stratégie doit faire l'objet de la meilleure prise en charge possible compte tenu de son état de santé et de l'état des connaissances du moment.

9.9.2. PATIENT PERDU DE VUE

On considère qu'un patient est perdu de vue s'il n'est pas possible de le recontacter (courrier, téléphone) et si son médecin traitant n'a plus de nouvelle de lui.

L'ARC coordonnateur, doit être informé qu'un patient est perdu de vue via la fiche correspondante du cahier d'observation.

Toute source d'information potentielle sera utilisée pour connaître le statut vital si le patient est perdu de vue avant la fin de la première année, notamment le registre du cancer et le RNIPP (Répertoire National d'Identification des Personnes Physiques).

9.9.3. AUTRES DEVIATIONS

Les déviations au protocole doivent être documentées et justifiées.

Sont considérées comme violations majeures les déviations portant sur :

- les aspects réglementaires,
- les critères d'éligibilité majeurs,
- le critère de jugement principal.

Toutes les violations majeures font l'objet d'une présentation au Conseil Scientifique de l'essai.

9.10. RETRAIT DE CONSENTEMENT ET ABANDON

Le patient qui souhaite abandonner ou retirer son consentement de participation à l'essai comme il est en droit de le faire à tout moment, n'est plus suivi dans le cadre du protocole, mais doit faire l'objet de la meilleure prise en charge possible compte tenu de son état de santé et de l'état des connaissances du moment.

9.10.1. ABANDON

Un patient est considéré en abandon d'essai quand il refuse de poursuivre le suivi prévu dans le cadre du protocole.

L'investigateur doit identifier la cause de l'abandon.

Les abandons d'essai doivent être notifiés rapidement à l'ARC coordonnateur de l'essai par fax via la fiche correspondante du cahier d'observation. Les raisons et la date d'abandon doivent être documentées.

9.10.2. RETRAIT DE CONSENTEMENT

Lorsqu'un patient retire son consentement de participation à l'essai, l'investigateur doit contacter l'ARC coordonnateur de l'essai qui lui transmettra la procédure à suivre.

Les données concernant le patient sont retirées de la base de données conformément à la loi relative à l'informatique et aux libertés (loi n°78-17 du 6 janvier 1978 modifiée, article 38 et les échantillons biologiques sont détruits.

10. ÉVÉNEMENTS INDÉSIRABLES

10.1. EVENEMENTS INDESIRABLES

10.1.1. DEFINITIONS

Un **événement indésirable** est tout symptôme, signe, maladie ou réaction qui se développe ou s'aggrave pendant la durée de participation d'un patient à une recherche biomédicale. Toute maladie ou accident intercurrent doit être considéré comme un événement indésirable.

Un événement indésirable n'est pas nécessairement lié à la stratégie d'intervention de l'étude ou aux conditions de la recherche.

10.1.2. NOTIFICATION D'UN EVENEMENT INDESIRABLE NON GRAVE

Les événements indésirables non graves doivent être notifiés par l'investigateur dans le cahier d'observation du patient. Ils sont recueillis et consignés dans le cahier à partir de la signature du consentement par le patient dans l'étude.

10.1.3. SUIVI D'UN EVENEMENT INDESIRABLE NON GRAVE

Après sa notification initiale, l'événement indésirable non grave doit obligatoirement faire l'objet d'un suivi jusqu'à sa résolution.

Si l'événement indésirable devient grave, il doit être déclaré et suivi conformément à la procédure indiquée au paragraphe 10.2.2.

10.2. EVENEMENTS INDESIRABLES GRAVES (EIG)

10.2.1. DEFINITIONS

Un **événement indésirable grave** répond à l'un au moins des critères suivants :

- est **fatal**
- met en jeu le **pronostic vital**
- nécessite ou prolonge une **hospitalisation**
- entraîne un **handicap** ou une **incapacité** ayant un caractère important ou durable
- se traduit par une anomalie ou une malformation congénitale

Est un événement **jugé potentiellement grave** par l'investigateur ou nécessitant une intervention pour prévenir l'évolution vers un des états précités.

Pour tout événement indésirable grave, l'investigateur se prononce sur le lien de causalité éventuel avec l'étude et le caractère attendu ou non de l'événement.

« Effet indésirable inattendu : tout effet indésirable dont la nature, la sévérité ou l'évolution ne concorde pas avec les informations relatives aux produits, actes pratiqués et méthodes utilisées au cours de la recherche. »

10.2.2. NOTIFICATION ET SUIVI DES EVENEMENTS INDESIRABLES GRAVES

Tout EIG doit être déclaré, s'il survient :

- à partir de la date de signature du consentement,
- pendant toute la durée de suivi du patient prévue par l'étude,

L'investigateur notifie au promoteur (Délégation à la Recherche Clinique et à l'Innovation du CHU de Bordeaux – DRCI) sans délai à compter du jour où il en a connaissance tous les événements indésirables graves, selon la procédure suivante **:**

- **L'investigateur transmet par fax au promoteur DRCI du CHU de Bordeaux (fax n° 05 56 79 49 26)** le formulaire de « *déclaration initiale d'un événement indésirable grave* » se trouvant dans le cahier d'observation du patient accompagné des copies anonymisées des résultats et comptes-rendus d'examens ou d'hospitalisation pertinents, sans omettre d'inscrire l'identifiant patient.

- La **DRCI** du CHU de Bordeaux transmet par courriel la fiche de déclaration et les documents joints :
    - au Centre de Méthodologie et de Gestion (USMR du CHU de Bordeaux) pour analyse et évaluation de l'imputabilité de l'EIG. Cette évaluation sera faite en collaboration avec l'investigateur ayant déclaré l'EIG
    - à l'investigateur coordonnateur du CHU de Bordeaux.

- Le Centre de Méthodologie et de Gestion (USMR) du CHU de Bordeaux transmet par fax le rapport d'imputabilité à la DRCI (copie Investigateur Coordonnateur et investigateur ayant déclaré l'EIG)

- La DRCI du CHU de Bordeaux déclare à l'autorité compétente et au CPP les EIG définis par les dispositions règlementaires.

Supprimé : '

Supprimé : '

Mise en forme : Puces et numéros

Supprimé : '

Supprimé : '

Supprimé : '

Supprimé : '

Supprimé :

10.2.3 SUIVI DES EVENEMENTS INDESIRABLES GRAVES

Après sa notification initiale, l'EIG doit faire l'objet d'un suivi jusqu'à sa résolution.

L'investigateur communique au promoteur (DRCI du CHU de Bordeaux) les informations complémentaires concernant les événements indésirables graves. **Cette notification s'effectue selon le même circuit que pour la notification initiale.**

10.2.4 EIG ATTENDUS

On distinguera les EIG attendus des EIG inattendus. **Les EIG attendus hormis les décès ne feront pas l'objet d'une déclaration immédiate**, ils seront transmis en même temps que les données de monitoring du cahier d'observation et seront inclus dans le rapport annuel. Leur recensement sera fréquent pour juger de l'imputabilité et identifier éventuellement des EIG inattendus.

Les EIG attendus concernent

- Les effets des traitements de chimiothérapie qui sont référencés dans les RCP des molécules utilisées,
- les pathologies liées à l'âge référencées dans MeDRA et leurs thérapeutiques référencées dans les RCP des molécules utilisées,
- l'évolution des pathologies cancéreuses référencées dans MeDRA.

11. SURVEILLANCE DE L'ESSAI

11.1. CONSEIL SCIENTIFIQUE

11.1.1. COMPOSITION

Il est composé des personnes suivantes : I Bourdel-Marchasson (président), les co-investigateurs de l'essai, les investigateurs cancérologues et gériatres de Limoges, Montpellier et Toulouse, G Chêne, la statisticienne de l'étude Christine Germain.

Les observateurs permanents sont : l'ARC et la coordonnatrice de l'action diététique.

11.1.2. RYTHME DES REUNIONS

Le Conseil Scientifique de l'étude se réunit avant le démarrage de l'étude puis au moins 2 fois par an jusqu'à sa clôture, physiquement ou téléphoniquement, dont une fois entre 1 et 2 mois après la date anniversaire de l'avis favorable donné à l'étude par l'autorité de santé pour validation du rapport annuel de sécurité.

11.1.3. ROLE

Il a pour mission de prendre toute décision importante à la demande de l'investigateur coordonnateur concernant la bonne marche de l'étude, le respect du protocole.

Il vérifie le respect de l'éthique.

Il s'informe auprès du centre investigateur coordonnateur et du Centre de Méthodologie et de Gestion de l'état d'avancement de l'étude, des problèmes éventuels et des résultats disponibles.

Il décide de toute modification pertinente du protocole nécessaire à la poursuite de l'étude, notamment :

- Les mesures permettant de faciliter le recrutement dans l'étude,
- Les amendements au protocole avant leur présentation au CPP et au ministre chargé de la Santé,
- Les décisions d'ouvrir ou de fermer des sites participant à l'essai,
- Les mesures qui assurent aux personnes participant à l'étude la meilleure sécurité,
- La discussion des résultats et la stratégie de publication de ces résultats.

Il approuve la constitution et la composition du comité indépendant qui est proposée par l'investigateur coordonnateur au cours de ses premières réunions.

Le Conseil Scientifique peut proposer (après avis du Comité Indépendant) de prolonger ou d'interrompre l'étude en cas de rythme d'inclusion trop lent, d'un trop grand nombre de perdus de vue, de violations majeures du protocole ou bien pour des raisons médicales et/ou administratives. Il précise les modalités éventuelles du suivi prolongé des patients inclus dans l'essai.

Il donne son avis pour toute utilisation de l'étude pour des études secondaires proposées par un ou plusieurs des investigateurs.

A l'issue de la réunion, le président du Conseil Scientifique doit informer le promoteur des décisions arrêtées. Les décisions concernant un amendement majeur ou une modification de budget doivent être approuvées par le promoteur.

11.2. COMITE INDEPENDANT DE SURVEILLANCE

11.2.1. COMPOSITION

Il sera constitué avant le démarrage de l'étude de deux praticiens, un oncologue et un gériatre autre que les investigateurs de l'étude, et d'un méthodologiste.

11.2.2. RYTHME DES REUNIONS

Le Comité Indépendant se réunit une première fois au démarrage de l'étude puis de façon régulière tout au long de l'étude, de sa propre initiative ou à la demande du Conseil Scientifique ou du promoteur.

11.2.3. ROLE

Ce comité a un rôle consultatif pour l'investigateur coordonnateur et le conseil scientifique. Il donne un avis général sur la marche de l'étude. Il peut aider à prendre en cours d'étude les décisions difficiles pour lesquelles un jugement indépendant est souhaitable. Il peut donner un avis dans les circonstances suivantes :

- Avis sur un arrêt prématuré de l'étude (parce que l'essai n'est plus réalisable, ou parce que les éléments permettant de conclure sont déjà rassemblés) ;
- Avis sur des modifications profondes du protocole devenues nécessaires à cause du recrutement ou du suivi de l'étude, ou pour tenir compte de données scientifiques nouvelles ;
- Analyse intermédiaire : interprétation des résultats de l'analyse, demande d'analyses ou de données complémentaires de l'étude.

L'avis du Comité Indépendant est transmis par écrit au promoteur et au Conseil Scientifique.

Toute modification profonde du protocole est soumise au Comité Indépendant par le Conseil Scientifique de l'étude avant d'être soumise au promoteur puis au Comité de Protection des Personnes et au ministre chargé de la Santé.

11.3. COMITE DE VALIDATION DES EVENEMENTS

11.3.1. COMPOSITION

Le Comité de Validation des Evénements est composé des personnes suivantes : I Bourdel-Marchasson, M Fonck, P Soubeyran, M Rainfray, la statisticienne de l'étude, l'ARC coordonnateur.

11.3.2. RYTHME DES REUNIONS

Il se réunit régulièrement en fonction du rythme des évènements.

11.3.3. ROLE

Le Comité de Validation des Evènements revoit les événements indésirables survenant au cours de l'essai et l'ensemble des autres critères de jugement. Il est chargé de valider les caractéristiques de certains événements, dont il aura établi au préalable la liste. La validation se fait en insu du groupe de traitement.

Ceci implique que tous les événements soient rapportés en temps réel au Centre de Méthodologie et de Gestion.

11.4. CENTRE DE METHODOLOGIE ET DE GESTION

Le Centre de Méthodologie et de Gestion est situé à l'Unité de Soutien Méthodologique à la Recherche Clinique et Epidémiologique du CHU de Bordeaux (USMR) (coordonnateur méthodologiste : Pr. G. Chêne).

11.4.1. COMPOSITION

L'équipe projet est composée d'un méthodologiste, d'un statisticien, d'un attachée de recherche clinique (ARC) référent, et d'un analyste programmeur.

Par ailleurs, deux opérateurs de saisie sont chargés de la double saisie des fiches du cahier d'observation.

11.4.2. RYTHME DES REUNIONS

Des réunions régulières de l'équipe projet permettent de suivre l'avancement de l'essai.

11.4.3. ROLE

L'équipe de l'USMR :
- supervise la conception méthodologique de l'étude,
- collabore à la conception du protocole avec l'investigateur coordonnateur. Elle finalise la rédaction du protocole, des notes d'informations et formulaires de consentement et du cahier d'observation avant soumission au CPP et au ministre chargé de la santé,
- déclare sa mise en conformité à la méthodologie de référence MR-001 de la CNIL.
- réalise le site Internet permettant l'accès sécurisé aux programmes de randomisation,
- réalise et gère la base de données informatique dédiée à l'étude,
- prépare en collaboration avec l'ARC du centre coordonnateur la mise en place et le suivi de l'essai, participe à la coordination à la logistique de l'essai,
- effectue l'analyse statistique des données,
- participe aux publications et autres valorisations des résultats de l'étude.

Le Centre de Méthodologie et de Gestion, en collaboration avec l'ARC du centre coordonnateur, participe à la préparation des dossiers facilitant la prise de décisions pour le Comité Indépendant de Surveillance, les résumés présentant l'état d'avancement de l'essai et les fichiers permettant l'analyse des données pour le Conseil Scientifique.

En collaboration avec l'ARC du centre coordonnateur, il informe le Conseil Scientifique du déroulement de l'essai, prépare les réunions des différents comités et les assemblées générales des investigateurs.

12. ASPECTS STATISTIQUES

12.1. METHODES STATISTIQUES EMPLOYEES

12.1.1. STRATEGIE D'ANALYSE

Une analyse intermédiaire est prévue lorsque la moitié des patients inclus sera parvenue à la fin de leur suivi, soit 1 an après avoir été inclus dans l'essai. Cette analyse porte sur le critère de jugement principal de l'essai, la survie à 1 an. Cette analyse permet, d'une part d'estimer l'intervalle de confiance de la différence entre les deux groupes, et d'autre part de tester l'existence d'une différence importante entre les deux groupes, qui peut conduire à interrompre l'essai pour généraliser, le plus rapidement possible, la prise en charge nutritionnelle spécifique testée dans l'essai à l'ensemble des patients à risque de dénutrition vus dans le service, si elle s'avère améliorer de manière importante la survie de ces patients. Afin de préserver un risque global α de 5%, on utilise la méthode d'O'Brien & Fleming pour réaliser l'analyse intermédiaire et l'analyse finale.

L'analyse principale est effectuée :

- en intention de traiter, c'est-à-dire que tous les patients randomisés sont inclus dans l'analyse dans le groupe dans lequel ils ont été initialement randomisés et toutes leurs données sont utilisées quels que soient les changements d'intervention au cours de l'essai.
- en utilisant la stratégie « donnée manquante = échec » : toute valeur manquante sera remplacée par la valeur correspondant à l'échec, c'est-à-dire le décès.

Cette analyse sera complétée par une analyse de sensibilité aux données manquantes en utilisant la stratégie du biais maximum.

La description se fait toujours globalement et par groupe d'intervention.

Les comparaisons entre groupes d'intervention se font systématiquement :

1) sans ajustement,

2) en ajustant sur la variable de stratification : le centre ; la durée de nutrition artificielle et le type de chimiothérapie.

Tous les tests sont effectués au risque d'erreur de première espèce α =5%.

12.1.2. Logiciels utilises

Les analyses sont réalisées avec le logiciel SAS® (version n°9.1).

12.2. Calcul de la taille d'etude

Dans cet essai, les patients sont randomisés en 2 groupes :

- groupe 1 (groupe « soins usuels ») : pas de soutien nutritionnel spécifique à l'étude (prise en charge habituelle de l'équipe d'oncologie)
- groupe 2 (groupe « soins usuels + intervention nutritionnelle spécifique ») : soutien nutritionnel individualisé

La comparaison des groupes porte sur la proportion de patients décédés à un an. L'essai est un essai de supériorité, test bilatéral. Une analyse intermédiaire est prévue lorsque la moitié des patients inclus aura atteint le temps principal de jugement, soit 1 an après avoir été randomisé dans l'essai.

On fait l'hypothèse que dans le groupe « soins usuels », sans soutien nutritionnel spécifique à l'essai, la proportion de patients décédés sera de 50% un an après l'inclusion dans l'essai, selon les résultats préliminaires observés sur la cohorte de 364 patients évalués selon le protocole de l'EGS (cf §2.2) et on considère que l'intervention nutritionnelle spécifique évaluée dans le cadre de cet essai (soutien nutritionnel individualisé) présenterait un intérêt clinique si la proportion de patients décédés diminuait de 10 % au moins.

Dans cette situation, le calcul fondé sur la formule du test du Chi2, avec un risque global α de 5% pour les 2 analyses prévues (intermédiaire et finale) et un risque α de 4,92% pour l'analyse finale selon la méthode d'O'Brien & Fleming pour tenir compte de l'analyse intermédiaire, et une puissance de $1-\beta$ de 80%, il faut inclure au moins 390 patients dans chaque groupe, soient 780 patients au total.

Afin de tenir compte d'une proportion de perdus de vue qui ne dépassera pas 5%, on décide de recruter 820 patients au total, soient 410 par groupe.

12.3. Plan d'analyse

12.3.1. Description des inclusions et du suivi

Les patients pré-inclus et non inclus dans l'essai sont décrits et comparés aux patients inclus pour les caractéristiques socio-démographiques et le typer de cancer.

Le nombre de patients inclus, la courbe des inclusions (évolution du nombre des patients inclus entre la première et la dernière inclusion), le nombre de visites théoriques correspondant au nombre de patients inclus, le nombre de visites réellement effectuées et le rapport des deux (nombre de visite effectuées/nombre de visites théoriques) sont présentés par groupe. La durée cumulée de suivi est calculée (somme du temps de participation pour chacun des patients inclus, c'est-à-dire de la différence, en nombre de jours, entre la date d'inclusion et la date des dernières nouvelles dans l'essai) et le rapport durée cumulée de suivi/durée cumulée attendue est présenté.

12.3.2. Patients inclus dans l'analyse

Ne peuvent être exclus de l'analyse que les patients qui présentent au moins une des conditions suivantes :

- Patients n'ayant jamais bénéficié de prise en charge nutritionnelle au cours du suivi (quelque soit le groupe de traitement : intervention usuelle ou intervention usuelle plus soutien nutritionnel spécifique à l'étude),
- Patients inclus à tort pour consentement non signé,
- Patients inclus à tort pour un critère majeur d'éligibilité non respecté,
- Patients ayant retiré leur consentement.

Cette décision d'exclusion est prise par le Conseil Scientifique après documentation de l'observation par le Centre de Méthodologie et de Gestion, en insu du groupe d'intervention et de l'évolution du patient après l'inclusion.

Les patients perdus de vue ou ayant abandonné l'essai sont tous inclus dans l'analyse.

12.3.3. Caracteristiques des patients a l'inclusion, avant le debut de l'intervention

Les patients sont décrits selon les variables suivantes :

- respect des critères d'éligibilité ;
- caractéristiques épidémiologiques ;
- caractéristiques cliniques ;
- caractéristiques biologiques ;
- caractéristiques de traitements ;

Une description des violations du protocole et des patients répartis selon ces violations est faite.

Une description des causes d'abandon est réalisée et les patients perdus de vue ou ayant abandonné l'essai sont décrits et comparés aux autres patients.

12.3.4. DESCRIPTION DES PRISES EN CHARGE USUELLES

Pour chaque centre, la prise en charge nutritionnelle usuelle effectuée dans le service sera décrite.

12.3.5. CRITERE DE JUGEMENT PRINCIPAL

Les proportions de décès sont décrites en termes d'effectif, de pourcentage et d'intervalle de confiance et comparés entre groupes d'intervention par un test du χ^2 ou du χ^2 corrigé selon les valeurs des effectifs attendus sous l'hypothèse d'indépendance.

Les courbes de survie sont estimées en termes de probabilité de survie et d'intervalle de confiance par la méthode de Kaplan-Meier. La date d'origine est la date de randomisation dans l'essai et le délai pour l'analyse est la différence entre la date de randomisation et la date de décès ou de dernier suivi avant un an si le patient est perdu de vue après les procédures de recherche de son statut vital. Les différents groupes d'intervention sont comparés par le test du log-rank.

12.3.6. CRITERE(S) DE JUGEMENT SECONDAIRE(S)

Les variables qualitatives sont décrites en termes d'effectif, de pourcentage et d'intervalle de confiance. Les comparaisons entre groupes se font par des tests du χ^2, ou du χ^2 corrigé, ou de Fisher exact, selon les valeurs des effectifs attendus sous l'hypothèse d'indépendance.

Les variables quantitatives sont décrites en termes d'effectif, moyenne, écart-type et intervalle de confiance de la moyenne, médiane, étendue et étendue interquartile. Les comparaisons se font par des tests du t de Student (comparaisons de moyennes) ou de Wilcoxon (comparaisons de distributions) selon la distribution de la variable d'intérêt. Des transformations pour normaliser la variable peuvent être effectuées si nécessaire.

La probabilité de survenue d'un événement d'intérêt est estimée grâce à la méthode de Kaplan-Meier. La date d'origine est la date d'inclusion dans l'essai et le délai de survenue est la différence entre la date d'inclusion et la date de diagnostic de l'événement d'intérêt ou la date de dernier suivi avant l'échéance des deux ans ou la date de décès (censure, prise en compte des risques compétitifs). Les différents groupes d'intervention sont comparés par le test du log-rank.

Les facteurs pronostiques cliniques et biologiques de la tolérance de la chimiothérapie et de la survie sont étudiés à l'aide d'un modèle de régression logistique ou d'un modèle de Cox selon le type de variable expliquée.

On essaye autant que possible d'associer une représentation graphique aux analyses.

Tous les tests sont effectués au risque d'erreur de première espèce $\alpha = 5\%$ (test bilatéral).

13. ASPECTS ETHIQUES ET RÉGLEMENTAIRES

13.1. CONFORMITE AUX TEXTES DE REFERENCE

Le promoteur et les investigateurs s'engagent à ce que cette étude soit réalisée en conformité avec la loi du 9 août 2004 (N°2004-806), ainsi qu'en accord avec les Bonnes Pratiques Cliniques (I.C.H. version 4 du 1er mai 1996)[1] et la déclaration d'Helsinki (Principes éthiques applicables aux recherches médicales sur des sujets humains, Tokyo 2004)[2] qui figure en annexe 5.

Le protocole, le(s) amendement(s), la note d'information et le formulaire de consentement de cette étude (cf. annexe 1) ont reçu l'avis favorable du Comité de Protection des Personnes (CPP) Sud Ouest et Outre Mer III le date (cf. annexe 2), et du ministre chargé de la santé .

1 http://www.ich.org/
2 http://www.wma.net/

La version n°1.10 du 08/02/2007 (avis favorable du CPP le 31 janvier 2007) est approuvée par l'investigateur coordonnateur et par le promoteur.

Les données enregistrées à l'occasion de cette étude font l'objet d'un traitement informatisé à l'Unité de Soutien Méthodologique à la Recherche Clinique et Epidémiologique du CHU de Bordeaux dans le respect de la loi n°78-17 relative à l'Informatique et aux Libertés du 6 janvier 1978 modifiée par la loi 2004-801 du 6 août 2004.

Le CHU de Bordeaux a adressé à la Commission Nationale de l'Informatique et des Libertés (C.N.I.L.)[3] l'engagement de conformité à la Méthodologie de Référence MR-001 (janvier 2006) attestant que les traitements des données réalisées par l'USMR sont conformes à l'ensemble des dispositions définies dans la MR-001.

La recherche du statut vital des patients perdus de vue sera effectuée par le biais du RNIPP (Répertoire National d'Identification des Personnes Physiques) après avis du CCTIRS et autorisation de la CNIL.

L'étude est conduite conformément au présent protocole, hormis dans les situations d'urgence nécessitant la mise en place d'actes thérapeutiques précis, les investigateurs s'engagent à respecter le protocole en tous points en particulier en ce qui concerne le recueil du consentement (cf. § 9.1 RECUEIL DU CONSENTEMENT) et la notification et le suivi des événements indésirables graves (cf. § 10.2 EVENEMENTS INDESIRABLES GRAVES).

13.2. AMENDEMENT AU PROTOCOLE

Toute modification substantielle c'est à dire toute modification de nature à avoir un impact significatif sur la protection des personnes, sur les conditions de validité et sur les résultats de la recherche, sur l'interprétation des documents scientifiques qui viennent appuyer le déroulement de la recherche ou sur les modalités de conduite de celle-ci, fait l'objet d'un amendement écrit qui est soumis au Conseil Scientifique de l'étude, au promoteur, puis au CPP et à ministre de la santé.

Après accord du CPP et du ministre de la santé, l'amendement est signé par l'investigateur coordonnateur et par le promoteur (de la même manière que pour la version définitive du protocole).

Les amendements mineurs, ne modifiant pas le sens du protocole, sont communiqués au CPP à titre d'information.

Tous les amendements au protocole doivent être portés à la connaissance de tous les investigateurs qui participent à l'étude. Les investigateurs s'engagent à en respecter le contenu.

13.3. CONFIDENTIALITE DES DONNEES

Afin de respecter l'anonymat des patients, chaque patient se verra attribuer un code anomyme d'identification, composé du numéro de centre investigateur sur 2 chiffres et d'un numéro de patient 4 chiffres et d'un code lettre (4 lettres), construit à partir des initiales de ses nom et prénom à l'aide d'une procédure rendant le code anonyme.

La procédure d'attribution du code anonyme est disponible dans le cahier d'observation.

13.4. ASSURANCE

Le CHU de Bordeaux, promoteur de cette étude, a souscrit un contrat d'assurance en responsabilité civile auprès de Gerling BioMédicinsure conformément aux dispositions de l'article L1121-10 du Code de la Santé Publique.

13.5. ARCHIVAGE ET STOCKAGE DES DOCUMENTS A LA FIN DE L'ETUDE

Les documents suivants relatifs à cette étude sont archivés conformément aux Bonnes Pratiques Cliniques :

Par les médecins investigateurs :

- pour une durée de 15 ans suivant la fin de l'étude

- Le protocole et les amendements éventuels au protocole
- Les classeurs d'observation

---

3 http://www.cnil.fr/

• Les dossiers source des participants ayant signé un consentement
• Tous les autres documents et courriers relatifs à l'étude

- pour une durée de 30 ans suivant la fin de l'étude

• L'exemplaire original des consentements éclairés signés des participants

Tous ces documents sont sous la responsabilité de l'investigateur pendant la durée réglementaire d'archivage.

13.6. ENREGISTREMENT DE L'ESSAI

Cette étude est enregistrée sur le site http://clinicaltrials.gov/ sous le n°*numéro.*

14. MONITORAGE ET BONNES PRATIQUES CLINIQUES

14.1. ORGANISATION GENERALE

Le monitorage est assuré par l'Attaché de Recherche Clinique (ARC) du centre coordonnateur, dans le respect de la réglementation en vigueur et des recommandations de Bonnes Pratiques Cliniques.

Le centre de Méthodologie et de Gestion est l'Unité de Soutien Méthodologique à la Recherche Clinique et Epidémiologique du CHU de Bordeaux (USMR).

A tout moment, l'investigateur coordonnateur, l'ARC en charge de l'essai et le Centre de Méthodologie et de Gestion peuvent être contactés pour toute question relative au protocole, à son application pratique, ou aux conduites à tenir devant certains événements

14.1.1. ROLE DE L'ARC REFERENT DE L'USMR

L'ARC de l'USMR assure la supervision de l'étude. Il intervient pour conseiller, aider l'ARC du centre coordonnateur, dans toutes les démarches administratives et réglementaires nécessaires. Il s'assure du bon déroulement de l'étude et du respect des BPC. L'ARC pourra être mandaté pour s'assurer de la qualité des données recueillies dans le cadre de l'étude

14.1.2. ROLE DE L'ARC DU CENTRE INVESTIGATEUR COORDONNATEUR

L'ARC du centre coordonnateur est chargé :

- d'organiser et de gérer la logistique de l'étude (visites d'ouverture, de monitorage et de fermeture des centres, mise à disposition des documents de l'étude dans les centres,
- de rédiger un manuel d'opération pour les centres investigateurs et de fournir les procédures applicables dans le cadre de l'étude,
- de vérifier que les consentements de participation sont correctement remplis et conservés,
- de contrôler l'exactitude et la complétude des données recueillies sur site,
- d'assurer un lien permanent avec les centres investigateurs,
- de gérer les inclusions et de tenir à jour la courbe des inclusions,
- de gérer les déclarations des événements indésirables graves selon les modalités décrites dans le § 10.2 EVENEMENTS INDESIRABLES GRAVES du protocole,
- de documenter et tenir à jour un bilan des événements indésirables graves et de leur évolution,
- de transmettre un bilan de l'évolution de l'étude tous les 15 jours à l' USMR : bilan des EIG, courbe des inclusions, inclusions par centre, visites de monitorage réalisées, survenue d'événements particuliers (arrêts prématurés,..) ou de modifications envisagées ou constatées dans le déroulement de l'étude, anomalies constatées lors des visites dans les centres,
- d'assurer la vérification et le codage des fiches des centres participants, et de rédiger les demandes de correction éventuelles,
- de gérer le flux des fiches et de les adresser au service de saisie.
- Et pour son propre centre (centre investigateur coordonnateur), d'aider les investigateurs à organiser le recrutement, l'inclusion, les demandes de randomisation et le suivi des patients.

14.2. CONSIGNES POUR LE RECUEIL DES DONNEES

Pour cet essai, le recueil des données est réalisé à partir d'un cahier papier.

Un cahier d'observation est attribué à chaque patient.

Un ensemble de consignes, facilitant le remplissage des fiches du cahier, la gestion et la logistique de l'étude est intégré au cahier d'observation.

Toutes les fiches du cahier d'observation doivent être complétées à partir du dossier médical par le médecin investigateur ou son représentant nommément désigné au fur et à mesure des visites (toutefois, les données recueillies restent sous la responsabilité du médecin investigateur principal du centre).

Le premier feuillet des fiches auto-dupliquantes (feuillet original) et une photocopie des fiches de recueil continu, datée et signée, seront adressés par courrier à l'ARC du centre coordonnateur après la visite du patient, accompagnés de la copie anonymisée des résultats des examens cliniques ou biologiques. Le deuxième feuillet des fiches auto-dupliquantes et l'original des fiches de recueil continu restent dans le cahier.

14.3. MONITORAGE DES CENTRES INVESTIGATEURS

Le centre investigateur est visité de façon régulière par l'ARC, mandaté par le promoteur.

Une visite a lieu lors de la mise en place de l'étude, une ou plusieurs ont lieu en cours d'étude et une visite en fin d'étude.

Les visites intermédiaires en cours d'étude ont pour objet de vérifier que :

- l'étude se déroule conformément au protocole et aux Bonnes Pratiques Cliniques et en particulier, que :
    - les consentements éclairés et écrits des patients ont bien été datés et signés par l'investigateur et par le patient, avant la réalisation de tout examen demandé par le protocole de l'étude,
    - que tous les événements indésirables graves ont bien été déclarés.
- les données recueillies dans les fiches du cahier d'observation sont conformes aux dossiers source.

Les données des patients sont consultées dans le respect de la confidentialité et du secret professionnel.

La vérification porte au minimum sur 100% des données réglementaires (consentement éclairé, et événements indésirables graves) et 5% des données suivantes : dates de visite, de début et fin de traitement de chimiothérapie, de prélèvement biologiques, de mesure du poids et des enquêtes diététiques.

Un rapport établissant l'ensemble des éléments vérifiés lors de la visite est systématiquement établi et transmis à l'ARC référent de Centre de Méthodologie et de Gestion et à l'investigateur du centre concerné.

En fin l'étude, tout matériel non utilisé est récupéré par l'ARC en charge de l'essai lors d'une visite de fermeture du centre.

14.4. AUDIT - INSPECTION

Un audit peut être réalisé à tout moment par des personnes mandatées par le promoteur et indépendantes des responsables de l'étude. Il a pour objectif de s'assurer de la qualité de l'étude, de la validité de ses résultats et du respect de la loi et des règlementations en vigueur.

Dans le même but, une inspection peut être réalisée, à tout moment, par des représentants des autorités de santé compétentes.

15. PUBLICATION DES RESULTATS

Toute communication écrite ou orale des résultats de l'essai doit recevoir l'accord préalable de l'investigateur coordonnateur et du Conseil Scientifique de l'essai.

L'analyse des données fournies par les centres investigateurs est réalisée par le Centre de Méthodologie et de Gestion. Cette analyse donne lieu à un rapport écrit qui est soumis au Conseil Scientifique pour accord. Ce rapport permet la préparation d'une ou plusieurs publication(s) dont la version finale doit recevoir l'approbation du Conseil Scientifique.

La publication des résultats principaux mentionne le nom du promoteur, de tous les investigateurs ayant inclus ou suivi des patients dans l'essai, la composition du Conseil Scientifique.

Le Conseil Scientifique est seul compétent pour décider de mentionner dans les publications relatives à l'étude le nom de toute autre personne s'il le juge souhaitable. Il sera néanmoins tenu par les règles ou procédures de publication en vigueur dans les essais du promoteur.

16. AUTRES RESULTATS ATTENDUS

En dehors de l'exploitation scientifique des résultats, cette étude devrait permettre de proposer des protocoles de soins basés sur la pratique diététique chez le patient en onco-gériatrie avec l'accord du Conseil Scientifique.

17. CESSION DES DONNEES

Le recueil et la gestion des données sont assurés par le Centre de Méthodologie et de Gestion.

Les conditions de cession de tout ou partie de la base de données de l'essai sont décidées par le promoteur de l'essai et font l'objet d'un contrat écrit.

18. REFERENCES BIBLIOGRAPHIQUES

1. Segura A, Pardo J, Jara C, Zugazabeitia L, Carulla J, de Las Penas R, et al. An epidemiological evaluation of the prevalence of malnutrition in Spanish patients with locally advanced or metastatic cancer. Clin Nutr 2005;24:801-14.
2. Dewys WD, Begg C, Lavin PT, Band PR, Bennett JM, Bertino JR, et al. Prognostic effect of weight loss prior to chemotherapy in cancer patients. Eastern Cooperative Oncology Group. Am J Med 1980;69:491-7.
3. van Halteren HK, Bongaerts GP, Wagener DJ. Cancer cachexia: what is known about its etiology and what should be the current treatment approach? Anticancer Res 2003;23:5111-5.
4. Davis MP, Dreicer R, Walsh D, Lagman R, LeGrand SB. Appetite and cancer-associated anorexia: a review. J Clin Oncol 2004;22:1510-7.
5. Morris H. Dysphagia in the elderly--a management challenge for nurses. Br J Nurs 2006;15:558-62.
6. Grant JP, Chapman G, Russell MK. Malabsorption associated with surgical procedures and its treatment. Nutr Clin Pract 1996;11:43-52.
7. Knox LS, Crosby LO, Feurer ID, Buzby GP, Miller CL, Mullen JL. Energy expenditure in malnourished cancer patients. Ann Surg 1983;197:152-62.
8. Bongaerts GP, van Halteren HK, Verhagen CA, Wagener DJ. Cancer cachexia demonstrates the energetic impact of gluconeogenesis in human metabolism. Med Hypotheses 2006;sous presse.
9. Todorov PT, McDevitt TM, Cariuk P, Coles B, Deacon M, Tisdale MJ. Induction of muscle protein degradation and weight loss by a tumor product. Cancer Res 1996;56:1256-61.
10. Russell ST, Tisdale MJ. Effect of a tumour-derived lipid-mobilising factor on glucose and lipid metabolism in vivo. Br J Cancer 2002;87:580-4.
11. Ferrigno D, Buccheri G. Anthropometric measurements in non-small-cell lung cancer. Support Care Cancer 2001;9:522-7.
12. Martin F, Santolaria F, Batista N, Milena A, Gonzalez-Reimers E, Brito MJ, et al. Cytokine levels (IL-6 and IFN-gamma), acute phase response and nutritional status as prognostic factors in lung cancer. Cytokine 1999;11:80-6.
13. Aleman MR, Santolaria F, Batista N, de La Vega M, Gonzalez-Reimers E, Milena A, et al. Leptin role in advanced lung cancer. A mediator of the acute phase response or a marker of the status of nutrition? Cytokine 2002;19:21-6.
14. Andreyev HJ, Norman AR, Oates J, Cunningham D. Why do patients with weight loss have a worse outcome when undergoing chemotherapy for gastrointestinal malignancies? Eur J Cancer 1998;34:503-9.
15. Buccheri G, Ferrigno D. Importance of weight loss definition in the prognostic evaluation of non-small-cell lung cancer. Lung Cancer 2001;34:433-40.
16. Lobato-Mendizabal E, Ruiz-Arguelles GJ, Marin-Lopez A. Leukaemia and nutrition. I: Malnutrition is an adverse prognostic factor in the outcome of treatment of patients with standard-risk acute lymphoblastic leukaemia. Leuk Res 1989;13:899-906.
17. Hickman DM, Miller RA, Rombeau JL, Twomey PL, Frey CF. Serum albumin and body weight as predictors of postoperative course in colorectal cancer. JPEN J Parenter Enteral Nutr 1980;4:314-6.
18. Bozzetti F, Gavazzi C, Miceli R, Rossi N, Mariani L, Cozzaglio L, et al. Perioperative total parenteral nutrition in malnourished, gastrointestinal cancer patients: a randomized, clinical trial. JPEN J Parenter Enteral Nutr 2000;24:7-14.
19. Shimizu Y, Nagaya N, Isobe T, Imazu M, Okumura H, Hosoda H, et al. Increased plasma ghrelin level in lung cancer cachexia. Clin Cancer Res 2003;9:774-8.
20. Neary NM, Small CJ, Wren AM, Lee JL, Druce MR, Palmieri C, et al. Ghrelin increases energy intake in cancer patients with impaired appetite: acute, randomized, placebo-controlled trial. J Clin Endocrinol Metab 2004;89:2832-6.
21. Daly JM, Weintraub FN, Shou J, Rosato EF, Lucia M. Enteral nutrition during multimodality therapy in upper gastrointestinal cancer patients. Ann Surg 1995;221:327-38.
22. van Bokhorst-De Van Der Schueren MA, Quak JJ, von Blomberg-van der Flier BM, Kuik DJ, Langendoen SI, Snow GB, et al. Effect of perioperative nutrition, with and without arginine supplementation, on nutritional status, immune function, postoperative morbidity, and survival in severely malnourished head and neck cancer patients. Am J Clin Nutr 2001;73:323-32.
23. Lundholm K, Daneryd P, Bosaeus I, Korner U, Lindholm E. Palliative nutritional intervention in addition to cyclooxygenase and erythropoietin treatment for patients with malignant disease: Effects on survival, metabolism, and function. Cancer 2004;100:1967-77.

24. Gianotti L, Braga M, Nespoli L, Radaelli G, Beneduce A, Di Carlo V. A randomized controlled trial of preoperative oral supplementation with a specialized diet in patients with gastrointestinal cancer. Gastroenterology 2002;122:1763-70.
25. Bozzetti F, Cozzaglio L, Gavazzi C, Bidoli P, Bonfanti G, Montalto F, et al. Nutritional support in patients with cancer of the esophagus: impact on nutritional status, patient compliance to therapy, and survival. Tumori 1998;84:681-6.
26. Isenring EA, Capra S, Bauer JD. Nutrition intervention is beneficial in oncology outpatients receiving radiotherapy to the gastrointestinal or head and neck area. Br J Cancer 2004;91:447-52.
27. Isenring E, Capra S, Bauer J. Patient satisfaction is rated higher by radiation oncology outpatients receiving nutrition intervention compared with usual care. J Hum Nutr Diet 2004;17:145-52.
28. Persson CR, Johansson BB, Sjoden PO, Glimelius BL. A randomized study of nutritional support in patients with colorectal and gastric cancer. Nutr Cancer 2002;42:48-58.
29. McGough C, Baldwin C, Frost G, Andreyev HJ. Role of nutritional intervention in patients treated with radiotherapy for pelvic malignancy. Br J Cancer 2004;90:2278-87.
30. Bauer J, Capra S, Battistutta D, Davidson W, Ash S. Compliance with nutrition prescription improves outcomes in patients with unresectable pancreatic cancer. Clin Nutr 2005;24:998-1004.
31. Evans WK, Nixon DW, Daly JM, Ellenberg SS, Gardner L, Wolfe E, et al. A randomized study of oral nutritional support versus ad lib nutritional intake during chemotherapy for advanced colorectal and non-small-cell lung cancer. J Clin Oncol 1987;5:113-24.
32. Arends J, Bodoky G, Bozzetti F, Fearon K, Muscaritoli M, Selga G, et al. ESPEN Guidelines on Enteral Nutrition: Non-surgical oncology. Clin Nutr 2006;25:245-59.
33. Cynober L, Alix E, Arnaud-Battandier F, Bonnefoy M, Brocker P, Cals MJ, et al. Personnes âgées. In: Martin A, AFSSA, editors. Apports nutritionnels conseillés pour la population française. Lassay les Chateaux: Edition Tec & Doc; 2001. p. 307-336.
34. Baldwin C, McGough C, Norman AR, Frost GS, Cunningham DC, Andreyev HJ. Failure of dietetic referral in patients with gastrointestinal cancer and weight loss. Eur J Cancer 2006.
35. Bourdel-Marchasson I, Dumas F, Pinganaud G, Emeriau JP, Decamps A. Audit of percutaneous endoscopic gastrostomy in long-term enteral feeding in a nursing home. Int J Qual Health Care 1997;9:297-302.
36. Howard L, Malone M. Clinical outcome of geriatric patients in the United States receiving home parenteral and enteral nutrition. Am J Clin Nutr 1997;66:1364-70.
37. Alvarez-Fernandez B, Garcia-Ordonez MA, Martinez-Manzanares C, Gomez-Huelgas R. Survival of a cohort of elderly patients with advanced dementia: nasogastric tube feeding as a risk factor for mortality. Int J Geriatr Psychiatry 2005;20:363-70.
38. Callahan CM, Haag KM, Weinberger M, Tierney WM, Buchanan NN, Stump TE, et al. Outcomes of percutaneous endoscopic gastrostomy among older adults in a community setting. J Am Geriatr Soc 2000;48:1048-54.
39. Bourdel-Marchasson I, Barateau M, Rondeau V, Dequae-Merchadou L, Salles-Montaudon N, Emeriau JP, et al. A multi-center trial of the effects of oral nutritional supplementation in critically ill older inpatients. GAGE Group. Groupe Aquitain Geriatrique d'Evaluation. Nutrition 2000;16:1-5.
40. Volkert D, Hubsch S, Oster P, Schlierf G. Nutritional support and functional status in undernourished geriatric patients during hospitalization and 6-month follow-up. Aging (Milano) 1996;8:386-95.
41. Bruce D, Laurance I, McGuiness M, Ridley M, Goldswain P. Nutritional supplements after hip fracture: poor compliance limits effectiveness. Clin Nutr 2003;22:497-500.
42. Potter JM, Roberts MA, McColl JH, Reilly JJ. Protein energy supplements in unwell elderly patients--a randomized controlled trial. JPEN J Parenter Enteral Nutr 2001;25:323-9.
43. Houwing RH, Rozendaal M, Wouters-Wesseling W, Beulens JW, Buskens E, Haalboom JR. A randomised, double-blind assessment of the effect of nutritional supplementation on the prevention of pressure ulcers in hip-fracture patients. Clin Nutr 2003;22:401-5.
44. Espaulella J, Guyer H, Diaz-Escriu F, Mellado-Navas JA, Castells M, Pladevall M. Nutritional supplementation of elderly hip fracture patients. A randomized, double-blind, placebo-controlled trial. Age Ageing 2000;29:425-31.
45. Lauque S, Arnaud-Battandier F, Gillette S, Plaze JM, Andrieu S, Cantet C, et al. Improvement of weight and fat-free mass with oral nutritional supplementation in patients with Alzheimer's disease at risk of malnutrition: a prospective randomized study. J Am Geriatr Soc 2004;52:1702-7.
46. Guigoz Y, Lauque S, Vellas BJ. Identifying the elderly at risk for malnutrition. The Mini Nutritional Assessment. Clin Geriatr Med 2002;18:737-57.
47. Beck AM, Ovesen L, Schroll M. Home-made oral supplement as nutritional support of old nursing home residents, who are undernourished or at risk of undernutrition based on the MNA. A pilot trial. Mini Nutritional Assessment. Aging Clin Exp Res 2002;14:212-5.

48. Hickson M, Bulpitt C, Nunes M, Peters R, Cooke J, Nicholl C, et al. Does additional feeding support provided by health care assistants improve nutritional status and outcome in acutely ill older in-patients?--a randomised control trial. Clin Nutr 2004;23:69-77.
49. Rainfray M, Fonck M, Mathoulin-Pelissier S, Blanc-Bisson C, Adriamampionona F, Vogt L, et al. Comprehensive Geriatric Assessment in elderly patients treated by chemotherapy for cancers. Prospective Study in Aquitaine Area. In: 18° Congress IAG; 2005 25th June-2nd July; Rio de Janeiro, Brazil; 2005.
50. Rainfray M, Bourdel-Marchasson I, Dehail P, Richard-Harston S. L'évaluation gérontologique : un outil de prévention des situations à risque chez les personnes âgées. Ann Med Interne 2002;153:397-402.
51. Stuck AE, Siu AL, Wieland GD, Adams J, Rubenstein LZ. Comprehensive geriatric assessment: a meta-analysis of controlled trials. Lancet 1993;342:1032-6.
52. Terret C. Management and geriatric assessment of cancer in the elderly. Expert Rev Anticancer Ther 2004;4:469-75.
53. McCorkle R, Strumpf NE, Nuamah IF, Adler DC, Cooley ME, Jepson C, et al. A specialized home care intervention improves survival among older post-surgical cancer patients. J Am Geriatr Soc 2000;48:1707-13.
54. Extermann M, Aapro M, Bernabei R, Cohen HJ, Droz JP, Lichtman S, et al. Use of comprehensive geriatric assessment in older cancer patients: recommendations from the task force on CGA of the International Society of Geriatric Oncology (SIOG). Crit Rev Oncol Hematol 2005;55:241-52.
55. Repetto L, Venturino A, Fratino L, Serraino D, Troisi G, Gianni W, et al. Geriatric oncology: a clinical approach to the older patient with cancer. Eur J Cancer 2003;39:870-80.
56. Extermann M, Chen H, Cantor AB, Corcoran MB, Meyer J, Grendys E, et al. Predictors of tolerance to chemotherapy in older cancer patients: a prospective pilot study. Eur J Cancer 2002;38:1466-73.
57. Fédération Nationale des centres de lutte contre le cancer
Bonnes pratiques pour la prise en charge diététique en cancérologie : la consultation. In: http://www.pharmclin.uhp-nancy.fr/astiercours/consultation_dietetique_integrale_0500.pdf; 2000.
58. Katz S, Downs TD, Cash HR, Grotz RC. Progress in development of the index of ADL. Gerontologist 1970;10:20-30.
59. Lawton MP, Brody EM. Assessment of older people: self-maintaining and instrumental activities of daily living. Gerontologist 1969;9:179-86.
60. Podsiadlo D, Richardson S. The timed "Up & Go": a test of basic functional mobility for frail elderly persons. J Am Geriatr Soc 1991;39:142-8.
61. Folstein MF, Folstein SE, McHugh PR. Mini-mental state : a practical method for grading the cognitive state of patients for the clinician. J Psychiatr Res 1975;12:189-98.
62. Yesavage JA. Geriatric depression scale: consistency of depressive symptoms over time. Percept Mot Skills 1991;73:1032.
63. Miller MD, Paradis CF, Houck PR, Mazumdar S, Stack JA, Rifai AH, et al. Rating chronic medical illness burden in geropsychiatric practice and research: application of the Cumulative Illness Rating Scale. Psychiatry Res 1992;41:237-48.
64. Aaronson NK, Ahmedzai S, Bergman B, Bullinger M, Cull A, Duez NJ, et al. The European Organization for Research and Treatment of Cancer QLQ-C30: a quality-of-life instrument for use in international clinical trials in oncology. J Natl Cancer Inst 1993;85:365-76.

ANNEXES

ANNEXE 1 NOTE D'INFORMATION – FORMULAIRE DE CONSENTEMENT

NOTE D'INFORMATION AU PATIENT

INTERVENTION NUTRITIONNELLE EN ONCO-GERIATRIE
CHEZ DES PATIENTS A RISQUE DE DENUTRITION
ETUDE INOGAD

Version n° 1.10 du 08 février 2007
Promoteur de l'étude : CHU de Bordeaux
Investigateur coordonnateur : Pr Isabelle Bourdel-Marchasson

Madame, Monsieur,

Votre médecin vous propose de participer à une étude clinique dont le Centre Hospitalier Universitaire de Bordeaux est le promoteur. Avant de prendre une décision, il est important que vous lisiez attentivement ces pages qui vous apporteront les informations nécessaires concernant les différents aspects de cette étude. N'hésitez pas à poser toutes les questions que vous jugerez utiles à votre médecin.

Votre participation est entièrement volontaire. Si vous ne désirez pas prendre part à cette étude, vous continuerez à bénéficier de la meilleure prise en charge médicale possible, conformément aux connaissances actuelles.

Pourquoi cette étude?

Un état nutritionnel fragile semble être associé à une moins bonne efficacité des traitements de chimiothérapie. Si la dénutrition est avérée, il est actuellement recommandé de mettre en œuvre les moyens nécessaires à son amélioration. Cependant, pour les personnes considérées comme « à risque de dénutrition », l'efficacité d'une prise en charge diététique dans l'amélioration de l'efficacité de la chimiothérapie n'a pas été étudiée. Il a paru important d'évaluer l'intérêt d'une telle prise en charge dans le traitement des cancers.

Quel est l'objectif de cette étude ?

Votre médecin vous propose de participer à un projet d'évaluation de votre état nutritionnel avant et pendant la mise en place de votre chimiothérapie, assorti d'un soutien nutritionnel si celui-ci s'avère nécessaire pour améliorer votre état de santé.

Si l'évaluation de votre état nutritionnel vous désigne comme étant à risque de dénutrition, une évaluation plus approfondie de votre état de santé sera effectuée. Elle consiste en plusieurs questionnaires et tests destinés à apprécier notamment vos conditions de vie, votre état physique et moral et une évaluation plus détaillée de votre état nutritionnel. Elle ne concerne pas directement le traitement de votre maladie tumorale qui sera déterminé en fonction des résultats du bilan clinique, biologique et radiologique habituel qui pourront cependant conduire à la mise en place de mesures de soutien pour favoriser la bonne conduite du traitement.

Ainsi, l'objectif de cette étude est d'évaluer l'impact d'un soutien nutritionnel spécifique visant à améliorer l'état de santé des sujets âgés à risque de dénutrition pendant leur traitement par chimiothérapie.

Comment va se dérouler cette étude ?

Dans les 4 différents centres interrégionaux, 820 personnes ayant donné leur consentement sont attendues pour participer à cette étude qui durera au total 4 ans. Si vous acceptez d'y prendre part et si vous répondez aux critères définis par le protocole, votre propre participation à cette étude durera 2 ans

Qui peut participer ?

Pour participer à cette étude, vous devez : avoir plus de 70 ans, être suivi pour une maladie cancéreuse relevant d'un traitement par chimiothérapie et avoir été identifié par votre médecin comme à risque de dénutrition.

Que vous demandera-t-on ?

Consultation d'évaluation : Après avoir lu cette note d'information et avoir discuté de l'étude avec votre médecin, si vous décidez d'y participer, vous devrez signer le formulaire de consentement confirmant votre accord de participation.

Un médecin fera le point avec vous des maladies ou interventions chirurgicales que vous avez subies, des médicaments que vous prenez actuellement (n'hésitez pas à les prendre avec vous ainsi que vos dernières

ordonnances). Ce médecin vous interrogera également pour évaluer notamment votre mémoire, vos capacités physiques et appréciera aussi vos habitudes alimentaires.

Par la suite, vous serez reçu(e) par une infirmière. Elle vous interrogera sur vos activités quotidiennes et vous aidera à remplir deux questionnaires concernant votre qualité de vie et votre état moral. Votre médecin programmera également les examens complémentaires nécessaires pour votre prise en charge, en particulier une prise de sang.
L'ensemble de cette évaluation durera entre 1/2 heure et une heure et servira à mieux vous connaître pour mieux guider la prise en charge de votre traitement.

Rythme des évaluations

Cette consultation vous sera proposée avant le traitement, et à la fin de la chimiothérapie. Elle sera organisée en même temps que votre traitement sans nécessiter de déplacement supplémentaire.

Traitement de la dénutrition

Si l’évaluation indique que vous êtes dénutri vous bénéficierez d’une prise en charge nutritionnelle adaptée à votre état par le service de diététique du centre de traitement.
Si vous êtes à risque de dénutrition, votre prise en charge nutritionnelle sera décidée par tirage au sort ; dans tous les cas, vous bénéficierez du meilleur suivi concernant votre maladie cancéreuse et soit vous serez suivi spécifiquement par une diététicienne qui vous donnera les conseils alimentaires pour améliorer si besoin vos apports, soit vous continuerez de bénéficier de la prise en charge habituelle proposée dans le centre où vous êtes soignés.

Quels sont vos droits ?

Votre médecin doit vous fournir toutes les explications nécessaires concernant cette étude. Si vous souhaitez vous en retirer à quelque moment que ce soit, et quel que soit le motif, vous continuerez à bénéficier du suivi médical et cela n'affectera en rien votre surveillance future.

Dans le cadre de la recherche biomédicale à laquelle le Centre Hospitalier Universitaire de Bordeaux vous propose de participer, un traitement informatique de vos données personnelles va être mis en œuvre pour permettre d’analyser les résultats de la recherche au regard de l’objectif de cette dernière qui vous a été présenté. A cette fin, les données médicales vous concernant et les données relatives à vos habitudes de vie seront transmises au Promoteur de la recherche ou aux personnes ou sociétés agissant pour son compte, en France ou à l’étranger. Ces données seront identifiées par un code de 4 lettres et 4 chiffres. Ces données pourront également, dans des conditions assurant leur confidentialité, être transmises aux autorités de santé françaises ou étrangères et à d’autres entités du Centre Hospitalier Universitaire de Bordeaux. Conformément aux dispositions de la loi relative à l’informatique aux fichiers et aux libertés, vous disposez d’un droit d’accès et de rectification. Vous disposez également d’un droit d’opposition à la transmission des données couvertes par le secret professionnel susceptibles d’être utilisées dans le cadre de cette recherche et d’être traitées. Vous pouvez également accéder directement ou par l’intermédiaire du médecin de votre choix à l’ensemble de vos données médicales en application des dispositions de l’article L1111-7 du Code de la Santé Publique. Ces droits s’exercent auprès du médecin qui vous suit dans le cadre de la recherche et qui connaît votre identité.

Conformément au Code de la Santé Publique :
- cette étude a obtenu un avis favorable du Comité de Protection des Personnes Sud Ouest et Outre Mer III le 31 janvier 2007 et l’autorisation du ministre chargé de la Santé
- le promoteur de cette étude, le CHU de Bordeaux (12 rue Dubernat, 33404 Talence), a souscrit une assurance de responsabilité civile auprès de Gerling Bio-Medicinsure (1680/90674)
- les personnes ayant subi un préjudice après participation à une recherche biomédicale peuvent faire valoir leurs droits auprès des commissions régionales de conciliation et d’indemnisation des accidents médicaux (Art L1121-10 et L1142-3 du code de la Santé Publique),
- lorsque cette étude sera terminée, vous serez tenu informé personnellement des résultats globaux par votre médecin dès que ceux-ci seront disponibles, si vous le souhaitez (art L1122-1 du code de la Santé Publique).

Après avoir lu cette note d’information, si vous acceptez de participer à cette étude, vous devez compléter et signer le formulaire de consentement de participation et un exemplaire du document complet vous sera remis.

FORMULAIRE DE CONSENTEMENT DU PATIENT

INTERVENTION NUTRITIONNELLE EN ONCO-GERIATRIE
CHEZ DES PATIENTS A RISQUE DE DENUTRITION
ETUDE INOGAD

Version n° 1.10 du 08 février 2007
Promoteur de l'étude : CHU de Bordeaux
Investigateur coordonnateur : Pr Isabelle Bourdel-Marchasson

Je soussigné(e) M.. *(nom, prénom),* certifie avoir lu et compris la note d'information qui m'a été remise. J'ai eu la possibilité de poser toutes les questions que je souhaitais au Dr .... *(nom, prénom)* qui m'a expliqué la nature, les objectifs, les risques potentiels et les contraintes liées à ma participation à cette étude.

Je comprends ces contraintes et les avantages liés à ma participation qui durera 2 ans.

Je connais la possibilité qui m'est réservée d'interrompre ma participation à cette étude à tout moment sans avoir à justifier ma décision et je ferai mon possible pour en informer le médecin cité ci-dessus ou en cas de changement le médecin qui me suit dans cette étude.

Cela ne remettra pas en cause la qualité des soins ultérieurs.

J'ai eu l'assurance que les décisions qui s'imposent pour ma santé seront prises à tout moment, conformément à l'état actuel des connaissances médicales.

J'ai pris connaissance que cette étude a reçu l'avis favorable du Comité de Protection de Personnes Sur Ouest et Outre Mer III, le 31 janvier 2007 et l'autorisation du Ministre chargé de la Santé.

Le promoteur de l'étude (CHU de Bordeaux, 12 rue Dubernat – 33404 Talence Cedex) a souscrit une assurance de responsabilité civile en cas de préjudice auprès de la société Gerling Bio Médicinsure (1680/90674) conformément à la loi.

J'accepte que seuls les médecins ou scientifiques impliqués dans le déroulement de cette étude, ainsi qu'éventuellement le représentant des Autorités de Santé, aient accès à l'information dans le respect le plus strict de la confidentialité.

J'accepte que les données enregistrées à l'occasion de cette étude puissent faire l'objet d'un traitement informatisé par le promoteur ou pour son compte.

J'ai bien noté que, conformément aux dispositions de la loi relative à l'informatique aux fichiers et aux libertés, je dispose d'un droit d'accès et de rectification. Je dispose également d'un droit d'opposition à la transmission des données couvertes par le secret professionnel susceptibles d'être utilisées dans le cadre de cette recherche et d'être traitées. Ces droits s'exercent auprès du médecin qui me suit dans le cadre de cette étude et qui connaît mon identité

Mon consentement ne décharge en rien l'investigateur et le promoteur de l'étude de leurs responsabilités à mon égard. Je conserve tous les droits garantis par la loi.

Les résultats globaux de l'étude me seront communiqués directement, si je le souhaite, conformément à la loi du 4 mars 2002 relative aux droits des malades et à la qualité du système de santé.

J'accepte librement de participer à cette recherche dans les conditions précisées dans la note d'information

Fait à .... le |__|__| |__|__| |__|__|__|__|

Nom et prénom du patient : ....
Signature du patient :

Le Docteur

Tampon du médecin

certifie avoir communiqué toute information utile concernant cette étude et s'engage à faire respecter les termes de ce consentement, conciliant le respect des droits et des libertés individuelles et les exigences d'un travail scientifique.

Fait à .... le |__|__| |__|__| |__|__|__|__|

Signature du médecin :

NOTE D'INFORMATION
A L'ATTENTION DU OU DES PERSONNES DE CONFIANCE DU PATIENT

**INTERVENTION NUTRITIONNELLE EN ONCO-GERIATRIE
CHEZ DES PATIENTS A RISQUE DE DENUTRITION
ETUDE INOGAD**

Version n° 1.10 du 08 février 2007
Promoteur de l'étude : CHU de Bordeaux
Investigateur coordonnateur : Pr Isabelle Bourdel-Marchasson

Madame, Monsieur,

Votre médecin vous propose la participation de votre proche à une recherche biomédicale dont le Centre Hospitalier Universitaire de Bordeaux est le promoteur. Avant de prendre une décision, il est important que vous lisiez attentivement ces pages qui vous apporteront les informations nécessaires concernant les différents aspects de cette étude. N'hésitez pas à poser toutes les questions que vous jugerez utiles à votre médecin.

La participation de votre proche est entièrement volontaire. Si vous ne désirez pas que votre proche prenne part à cette étude, il continuera à bénéficier de la meilleure prise en charge médicale possible, conformément aux connaissances actuelles.

Pourquoi cette étude?

Un état nutritionnel fragile semble être associé à une moins bonne efficacité des traitements de chimiothérapie. Si la dénutrition est avérée, il est actuellement recommandé de mettre en œuvre les moyens nécessaires à son amélioration. Cependant, pour les personnes considérées comme « à risque de dénutrition », l'efficacité d'une prise en charge diététique dans l'amélioration de l'efficacité de la chimiothérapie n'a pas été étudiée. Il a paru important d'évaluer l'intérêt d'une telle prise en charge dans le traitement des cancers.

Quel est l'objectif de cette étude ?

Votre médecin vous propose que votre proche participe à un projet d'évaluation de son état nutritionnel avant et pendant la mise en place de sa chimiothérapie, assorti d'un soutien nutritionnel si celui-ci s'avère nécessaire pour améliorer son état de santé.

Si l'évaluation de son état nutritionnel le désigne comme étant à risque de dénutrition, une évaluation plus approfondie de son état de santé sera effectuée. Elle consiste en plusieurs questionnaires et tests destinés à apprécier notamment ses conditions de vie, son état physique et moral et une évaluation plus détaillée de son état nutritionnel. Elle ne concerne pas directement le traitement de sa maladie tumorale qui sera déterminé en fonction des résultats du bilan clinique, biologique et radiologique habituel qui pourront cependant conduire à la mise en place de mesures de soutien pour favoriser la bonne conduite du traitement.

Ainsi, l'objectif de cette étude est d'évaluer l'impact d'un soutien nutritionnel spécifique visant à améliorer l'état de santé des sujets âgés à risque de dénutrition pendant leur traitement par chimiothérapie.

Comment va se dérouler cette étude ?

Dans les 4 différents centres interrégionaux, 820 personnes ayant donné leur consentement sont attendues pour participer à cette étude qui durera au total 4 ans. Si vous acceptez que votre proche y prenne part et si il répond aux critères définis par le protocole, sa propre participation à cette étude durera 2 ans.

Qui peut participer ?

Pour participer à cette étude, votre proche doit : avoir plus de 70 ans, être suivi pour une maladie cancéreuse relevant d'un traitement par chimiothérapie et avoir été identifié par votre médecin comme à risque de dénutrition.

Que vous demandera-t-on ?

Consultation d'évaluation : Après avoir lu cette note d'information et avoir discuté de l'étude avec votre médecin, si vous décidez que votre proche y participe, vous devrez signer le formulaire de consentement confirmant votre accord de participation.

Un médecin fera le point avec vous et votre proche des maladies ou interventions chirurgicales que votre proche a subies, des médicaments qu'il prend actuellement (n'hésitez pas à les prendre avec vous ainsi que ses dernières ordonnances). Ce médecin interrogera également votre proche pour évaluer notamment sa mémoire, ses capacités physiques et appréciera aussi ses habitudes alimentaires.

Par la suite, votre proche sera reçu par une infirmière. Elle l'interrogera sur ses activités quotidiennes et l'aidera à remplir deux questionnaires concernant sa qualité de vie et son état moral. Votre médecin

programmera également les examens complémentaires nécessaires pour sa prise en charge, en particulier une prise de sang.
L'ensemble de cette évaluation durera entre 1/2 heure et une heure et servira à mieux connaître votre proche pour mieux guider la prise en charge de son traitement.

Rythme des évaluations

Cette consultation sera proposée à votre proche avant le traitement, et à la fin de la chimiothérapie. Elle sera organisée en même temps que son traitement sans nécessiter de déplacement supplémentaire.

Traitement de la dénutrition

Si l'évaluation indique que votre proche est dénutri, il bénéficiera d'une prise en charge nutritionnelle adaptée à son état par le service de diététique du centre de traitement.
Si votre proche est à risque de dénutrition, sa prise en charge nutritionnelle sera décidez par tirage au sort ; dans tous les cas, il bénéficiera du meilleur suivi concernant sa maladie cancéreuse et soit il sera suivi spécifiquement par une diététicienne qui lui donnera les conseils alimentaires pour améliorer si besoin ses apports, soit il continuera de bénéficier de la prise en charge habituelle proposée dans le centre où il est soigné.

Quels sont vos droits ?

Votre médecin doit vous fournir toutes les explications nécessaires concernant cette étude. Si vous souhaitez en retirer votre proche à quelque moment que ce soit, et quel que soit le motif, il continuera à bénéficier du suivi médical et cela n'affectera en rien sa surveillance future.

Dans le cadre de la recherche biomédicale à laquelle le Centre Hospitalier Universitaire de Bordeaux vous propose que votre proche participe, un traitement informatique de ses données personnelles va être mis en œuvre pour permettre d'analyser les résultats de la recherche au regard de l'objectif de cette dernière qui vous a été présenté. A cette fin, les données médicales le concernant et les données relatives à ses habitudes de vie seront transmises au Promoteur de la recherche ou aux personnes ou sociétés agissant pour son compte, en France ou à l'étranger. Ces données seront identifiées par un code de 4 lettres et X chiffres. Ces données pourront également, dans des conditions assurant leur confidentialité, être transmises aux autorités de santé françaises ou étrangères et à d'autres entités du CHU de Bordeaux. Conformément aux dispositions de la loi relative à l'informatique aux fichiers et aux libertés, vous disposez d'un droit d'accès et de rectification. Vous disposez également d'un droit d'opposition à la transmission des données couvertes par le secret professionnel susceptibles d'être utilisées dans le cadre de cette recherche et d'être traitées. Vous pouvez également accéder directement ou par l'intermédiaire du médecin de votre choix à l'ensemble de vos données médicales en application des dispositions de l'article L1111-7 du Code de la Santé Publique. Ces droits s'exercent auprès du médecin qui vous suit dans le cadre de la recherche et qui connaît votre identité.

Conformément au Code de la Santé Publique :
- cette étude a obtenu un avis favorable du Comité de Protection des Personnes Sud Ouest et Outre Mer III le 31 janvier 2007 et l'autorisation du ministre chargé de la Santé
- le promoteur de cette étude, le CHU de Bordeaux (12 rue Dubernat, 33404 Talence), a souscrit une assurance de responsabilité civile auprès de Gerling Bio-Medicinsure (1680/90674)
- les personnes ayant subi un préjudice après participation à une recherche biomédicale peuvent faire valoir leurs droits auprès des commissions régionales de conciliation et d'indemnisation des accidents médicaux (Art L1121-10 et L1142-3 du code de la Santé Publique),
- lorsque cette étude sera terminée, vous serez tenu informé personnellement des résultats globaux par votre médecin dès que ceux-ci seront disponibles, si vous le souhaitez (art L1122-1 du code de la Santé Publique).

Après avoir lu cette note d'information, si vous acceptez de participer à cette étude, vous devez compléter et signer le formulaire de consentement de participation et un exemplaire du document complet vous sera remis.

FORMULAIRE DE CONSENTEMENT
A L'ATTENTION DU OU DES PERSONNES DE CONFIANCE DU PATIENT

INTERVENTION NUTRITIONNELLE EN ONCO-GERIATRIE CHEZ DES PATIENTS A RISQUE DE DENUTRITION ETUDE INOGAD

Version n° 1.10 du 08 février 2007
Promoteur de l'étude : CHU de Bordeaux
Investigateur coordonnateur : Pr Isabelle Bourdel-Marchasson

Nous soussignés, M...(*nom, prénom, lien de parenté avec le patient),* et M ....(*nom, prénom, lien de parenté avec le patient),* représentant(s) légal (aux), personne de confiance de ....(*nom, prénom)* certifions avoir lu et compris la note d'information qui m'a été remise. Nous avons eu la possibilité de poser toutes les questions que nous souhaitions au Dr ....(*nom, prénom)* qui nous a expliqué la nature, les objectifs, les risques potentiels et les contraintes liées à la participation de notre proche à cette étude.

Nous comprenons ces contraintes et les avantages liés à sa participation qui durera 2 ans.

Nous connaissons la possibilité qui nous est réservée d'interrompre la participation de notre proche à cette étude à tout moment sans avoir à justifier notre décision et nous ferons notre possible pour en informer le médecin qui le suit dans cette étude.

Cela ne remettra naturellement pas en cause la qualité des soins ultérieurs.

Nous avons eu l'assurance que les décisions qui s'imposent pour la santé de notre proche seront prises à tout moment, conformément à l'état actuel des connaissances médicales.

Nous avons pris connaissance que cette étude a reçu l'avis favorable du Comité de Protection de Personnes Sur Ouest et Outre Mer III, le 31 janvier 2007 et l'autorisation du Ministre chargé de la Santé.

Le promoteur de l'étude (CHU de Bordeaux, 12 rue Dubernat – 33404 Talence Cedex) a souscrit une assurance de responsabilité civile en cas de préjudice auprès de la société Gerling Bio Médicinsure (1680/90674) conformément à la loi.

Nous acceptons que seuls les médecins ou scientifiques impliqués dans le déroulement de cette étude, ainsi qu'éventuellement le représentant des Autorités de Santé, aient accès à l'information dans le respect le plus strict de la confidentialité.

Nous acceptons que les données enregistrées à l'occasion de cette étude puissent faire l'objet d'un traitement informatisé par le promoteur ou pour son compte.

Nous avons bien noté que, conformément aux dispositions de la loi relative à l'informatique aux fichiers et aux libertés, notre proche dispose d'un droit d'accès et de rectification. Notre proche dispose également d'un droit d'opposition à la transmission des données couvertes par le secret professionnel susceptibles d'être utilisées dans le cadre de cette recherche et d'être traitées. Ces droits s'exercent auprès du médecin qui suit notre proche dans le cadre de cette étude et qui connaît son identité

Notre consentement ne décharge en rien les organisateurs de l'étude de leurs responsabilités. Nous conservons tous les droits garantis par la loi.

Si nous le souhaitons, les résultats des examens de notre proche et de l'étude nous seront communiqués directement, conformément à la loi du 4 mars 2002 relative aux droits des malades et à la qualité du système de santé.

Nous acceptons librement que notre proche participe à cette recherche dans les conditions précisées dans la note d'information

Fait à .... le |__|__| |__|__| |__|__|__|__|

Nom et prénom du (des) personne(s) de confiance : ....
En Qualité de : ....
Signature du (des) personne(s) de confiance:

Le Docteur

Tampon du médecin

certifie avoir communiqué toute information utile concernant cette étude et s'engage à faire respecter les termes de ce consentement, conciliant le respect des droits et des libertés individuelles et les exigences d'un travail scientifique.

Fait à .... le |__|__| |__|__| |__|__|__|__|

Signature du médecin :

ANNEXE 2 : AVIS DU CPP

ANNEXE 3 : LISTE DES CENTRES INVESTIGATEURS

Centre	Chef de Service ou de Département	Investigateur Principal	Co-investigateurs	Adresse
Bordeaux, CHU	Pr J-P Emeriau	Pr I Bourdel-Marchasson	Dr M Floccia<br>Dr C Mertens	Département de gériatrie<br>Hôpital Xavier Arnozan<br>33604 Pessac cedex
Bordeaux, CHU	Pr JP Maire	Dr D Smith	Dr H Demeaux	Service radiothérapie<br>Hôpital Saint André<br>1 rue Jean Burguet<br>33076 Bordeaux
Bordeaux, CHU	Pr C Balabaud	Dr JF Blanc		Service hépato-gastroentérologie<br>Hôpital Saint André<br>1 rue Jean Burguet<br>33076 Bordeaux
Bordeaux, CHU	Pr P Couzigou	Dr X Adhoute	Dr E Terrebonne	Service hépato-gastroentérologie<br>Hôpital Haut Lévêque<br>33604 Pessac cedex
Bordeaux Institut Bergonié	Pr J Reiffers	Dr M Fonck	Pr P Soubeyran<br>Dr A Floquet<br>Dr Y Bécouarn<br>Dr B Bui<br>Dr H Eghbali<br>Dr N Madranges<br>Dr F Chomy<br>Dr G Etienne<br>Dr N Houédé<br>Dr L Mauriac<br>Dr M Debled<br>Dr R Brunet	Institut Bergonié, CLCC<br>229 cours de l'Argonne<br>33076 Bordeaux cedex
Clinique Bordeaux Nord		Dr C Lécaille		Polyclinique Bordeaux Nord Aquitaine<br>15-33 rue Claude Boucher<br>33300 Bordeaux
CH Agen	Dr L Vogt	Dr L Vogt		Département de gériatrie<br>Hôpital Monbran<br>47923 Agen
CH Dax	Dr JL Perié	Dr JL Perié	Dr F Fonade	Service de gériatrie<br>CH Dax<br>Boulevard Yves du Manoir<br>40107 Dax
CH Dax	Dr P Rémuzon	Dr L Gautier-Felizot	Dr F Lifermann<br>Dr E Poncin	Service radiothérapie<br>CH Dax<br>Boulevard Yves du Manoir<br>40107 Dax
CH Libourne	Dr J Ceccaldi	Dr J Ceccaldi		Service de médecine<br>CH Robert Boulin<br>112 rue de la Marne<br>33505 Libourne Cedex
CH Libourne	Dr D Auby	Dr D Auby		Service de gastroentérologie<br>CH Robert Boulin<br>112 rue de la Marne<br>33505 Libourne Cedex
CH Mont de Marsan	Dr J Dauba	Dr J Dauba	Dr M Becerro-Hallard<br>Dr P Texereau<br>Dr S DeWitte	Service de médecine ambulatoire<br>CH Layné<br>Avenue Pierre de Coubertin<br>40000 Mont de Marsan

Centre	Chef de Service ou de Département	Investigateur Principal	Co-investigateurs	Adresse
CH Mont de Marsan	Dr L Hallard	Dr L Hallard		Service de gériatrie<br>CH Nouvielle<br>Route de Bretagne de Marsan<br>40000 Mont de Marsan
CH François Mitterand	Dr C Dagada	Dr C Dagada		Service oncologie<br>CH François Mitterand<br>Boulevard des hautes rives<br>64000 Pau
Clinique Francheville	Dr L Cany	Dr L Cany	Dr B Levaché	Service radiothérapie<br>Clinique Francheville<br>38 boulevard de Vérone BP 4063<br>24004 Périgueux
CH Périgueux	Dr Y Delhoume	Dr M Payssé		Hôpital de jour<br>CH Périgueux<br>80 avenue Georges Pompidou BP 9052<br>24019 Périgueux
CH Saint-Cyr	Dr E Brudieux	Dr E Brudieux		Service médecine 1<br>CH Saint-Cyr<br>2 bis boulevard Saint Cyr<br>47300 Villeneuve sur Lot
CHU Limoges	Pr N Tubiana-Mathieu	Dr JL LABOUREY	Pr N Tubiana-Mathieu<br>Dr J Martin	Oncologie médicale<br>CHRU de Limoges<br>2 avenue Martin Luther King<br>87042 Limoges cedex<br>Tel 0555056268 - Fax 0555056271
CHU Limoges	Pr T DANTOINE	Pr T Dantoine		Médecine Interne Gériatrique<br>Centre Dupuytren, CHU de Limoges<br>2 avenue Martin Luther King<br>87042 Limoges cedex<br>tel : 05 55 05 65 44 - fax : : 05 55 05 65 45
CLCC Val d'Aurelle	Dr P SENESSE	Dr P SENESSE	Dr S Culine<br>Dr G Romieu<br>Dr M Fabbro<br>Dr L Cristol	CRLC Val d'Aurelle<br>Service d'oncologie digestive et nutrition<br>208 rue des Apothicaires<br>34298 MONTPELLIER Cedex 05<br>tel : 0467618554 - fax : 0467613738
CLCC Claudius Regaud	Pr Roché	Dr L Mourey		INSTITUT CLAUDIUS REGAUD<br>20-24 rue du Pont Saint-Pierre<br>31052 TOULOUSE CEDEX<br>Tel : 05 61 42 41 74 - Fax : 05 61 59 37 47
CHU Toulouse	Pr B Vellas	Dr L Balardy		CHU Purpan Casselardit<br>170 av de Casselardit<br>31300 Toulouse

ANNEXE 4 : QUESTIONAIRES D'EVALUATION GERIATRIQUE STANDARDISEE

MNA: Mini Nutritional Assessment

MMS: Mini-Mental Status

ADL: Activities in Daily Living

IADL: Instrumental Activities in Daily Living

GDS15: Geriatric Depression Scale 15 questions

QLQ-C30: EORTC Quality of Life questionnaire

CIRS-G: Cumulative Illness Rating Scale for Geriatrics

NESTLÉ NUTRITION SERVICES

Nestlé

Evaluation de l'état nutritionnel
Mini Nutritional Assessment MNA™

Nom: Prénom: Sexe: Date:

Age: Poids, kg: Taille en cm: Hauteur du genou, cm:

Répondez à la première partie du questionnaire en indiquant le score approprié pour chaque question. Additionnez les points de la partie Dépistage, si le résultat est égal à 11 ou inférieur, complétez le questionnaire pour obtenir l'appréciation précise de l'état nutritionnel.

Dépistage

A Le patient présente-t-il une perte d'appétit?
A-t-il mangé moins ces 3 derniers mois par manque d'appétit, problèmes digestifs, difficultés de mastication ou de déglutition?
0 - anorexie sévère
1 - anorexie modérée
2 - pas d'anorexie ☐

B Perte récente de poids (<3 mois)
0 - perte de poids > 3 kg
1 - ne sait pas
2 - perte de poids entre 1 et 3 kg
3 - pas de perte de poids ☐

C Motricité
0 - du lit au fauteuil
1 - autonome à l'intérieur
2 - sort du domicile ☐

D Maladie aiguë ou stress psychologique lors des 3 derniers mois?
0 - oui 2 - non ☐

E Problèmes neuropsychologiques
0 - démence ou dépression sévère
1 - démence ou dépression modérée
2 - pas de problème psychologique ☐

F Indice de masse corporelle (IMC = poids / (taille)^2 en kg/m^2)
0 - IMC < 19
1 - 19 ≤ IMC < 21
2 - 21 ≤ IMC < 23
3 - IMC ≥ 23 ☐

Score de dépistage (sous-total max. 14 points) ☐ ☐

12 points ou plus — normal pas besoin de continuer l'évaluation

11 points ou moins — possibilité de malnutrition – continuez l'évaluation

Evaluation globale

G Le patient vit-il de façon indépendante à domicile?
0 - non 1 - oui ☐

H Prend plus de 3 médicaments
0 - oui 1 - non ☐

I Escarres ou plaies cutanées?
0 - oui 1 - non ☐

J Combien de véritables repas le patient prend-il par jour?
0 - 1 repas
1 - 2 repas
2 - 3 repas ☐

K Consomme-t-il?
- Une fois par jour au moins des produits laitiers? oui ☐ non ☐
- Une ou deux fois par semaine des œufs ou des légumineuses? oui ☐ non ☐
- Chaque jour de la viande, du poisson ou de la volaille? oui ☐ non ☐

0,0 - si 0 ou 1 oui
0,5 - si 2 oui
1,0 - si 3 oui ☐,☐

L Consomme-t-il deux fois par jour au moins des fruits ou des légumes?
0 - non 1 - oui ☐

M Combien de verres de boissons consomme-t-il par jour? (eau, jus, café, thé, lait, vin, bière…)
0,0 - moins de 3 verres
0,5 - de 3 à 5 verres
1,0 - plus de 5 verres ☐,☐

N Manière de se nourrir
0 - nécessite une assistance
1 - se nourrit seul avec difficulté
2 - se nourrit seul sans difficulté ☐

O Le patient se considère-t-il bien nourri? (problèmes nutritionnels)
0 - malnutrition sévère
1 - ne sait pas ou malnutrition modérée
2 - pas de problème de nutrition ☐

P Le patient se sent-il en meilleure ou en moins bonne santé que la plupart des personnes de son âge?
0,0 - moins bonne
0,5 - ne sait pas
1,0 - aussi bonne
2,0 - meilleure ☐,☐

Q Circonférence brachiale (CB en cm)
0,0 - CB < 21
0,5 - CB ≤ 21 CB ≤ 22
1,0 - CB > 22 ☐,☐

R Circonférence du mollet (CM en cm)
0 - CM < 31 1 - CM ≥ 31 ☐

Evaluation globale (max. 16 points) ☐ ☐,☐

Score de dépistage ☐ ☐

Score total (max. 30 points) ☐ ☐,☐

Appréciation de l'état nutritionnel

de 17 à 23,5 points — risque de malnutrition ☐

moins de 17 points — mauvais état nutritionnel ☐

Ref.: Guigoz Y, Vellas B and Garry PJ. 1994. Mini Nutritional Assessment: A practical assessment tool for grading the nutritional state of elderly patients. *Facts and Research in Gerontology*. Supplement #2:15-59.
Rubenstein LZ, Harker J, Guigoz Y and Vellas B. Comprehensive Geriatric Assessment (CGA) and the MNA: An Overview of CGA, Nutritional Assessment, and Development of a Shortened Version of the MNA. In: "Mini Nutritional Assessment (MNA): Research and Practice in the Elderly". Vellas B, Garry PJ and Guigoz Y, editors. Nestlé Nutrition Workshop Series. Clinical & Performance Programme, vol. 1. Karger, Bâle, in press.

© 1998 Société des Produits Nestlé S.A., Vevey, Switzerland, Trademark Owners

0898 F

MINI MENTAL STATE EXAMINATION (MMS)

(Version consensuelle du GRECO)

Orientation / 10

Je vais vous poser quelques questions pour apprécier comment fonctionne votre mémoire. Les unes sont très simples, les autres un peu moins. Vous devez répondre du mieux que vous pouvez.

Quelle est la date complète d'aujourd'hui ?

Si la réponse est incorrecte ou incomplète, posez les questions restées sans réponse, dans l'ordre suivant :

1. En quelle année sommes-nous ? ❒
2. En quelle saison ? ❒
3. En quel mois ? ❒
4. Quel jour du mois ? ❒
5. Quel jour de la semaine ? ❒

Je vais vous poser maintenant quelques questions sur l'endroit où nous trouvons.

6. Quel est le nom de l'hôpital où nous sommes ? ❒
7. Dans quelle ville se trouve-t-il ? ❒
8. Quel est le nom du département dans lequel est située cette ville ? ❒
9. Dans quelle province ou région est située ce département ? ❒
10. A quel étage sommes-nous ? ❒

Apprentissage / 3

Je vais vous dire trois mots ; je vous voudrais que vous me les répétiez et que vous essayiez de les retenir car je vous les redemanderai tout à l'heure.

11. Cigare ou Citron ou Fauteuil ❒
12. Fleur ou Clé ou Tulipe ❒
13. Porte ou Ballon ou Canard ❒

Répéter les 3 mots.

Attention et calcul / 5

Voulez-vous compter à partir de 100 en retirant 7 à chaque fois ?

14. 93 ❒
15. 86 ❒
16. 79 ❒
17. 72 ❒
18. 65 ❒

Pour tous les sujets, même pour ceux qui ont obtenu le maximum de points, demander :

Voulez-vous épeler le mot MONDE à l'envers ?

Rappel / 3

Pouvez-vous me dire quels étaient les 3 mots que je vous ai demandés de répéter et de retenir tout à l'heure ?

11. Cigare ou Citron ou Fauteuil ❒
12. Fleur ou Clé ou Tulipe ❒
13. Porte ou Ballon ou Canard ❒

Langage / 8

Montrer un crayon. 22. Quel est le nom de cet objet ? ❒

Montrer votre montre. 23. Quel est le nom de cet objet ? ❒

24. Ecoutez bien et répétez après moi : « PAS DE MAIS, DE SI, NI DE ET » ❒

Poser une feuille de papier sur le bureau, la montrer au sujet en lui disant : « Ecoutez bien et faites ce que je vais vous dire :

25. Prenez cette feuille de papier avec votre main droite, ❒
26. Pliez-la en deux, ❒
27. Et jetez-la par terre. » ❒

Tendre au sujet une feuille de papier sur laquelle est écrit en gros caractère : « FERMEZ LES YEUX » et dire au sujet :

28. « Faites ce qui est écrit ». ❒

Tendre au sujet une feuille de papier et un stylo, en disant :

29. « Voulez-vous m'écrire une phrase, ce que vous voulez, mais une phrase entière. » ❒

Praxies constructives / 1

Tendre au sujet une feuille de papier et lui demander : 30. « Voulez-vous recopier ce dessin ? » ❒

**TOTAL**	**/30**	/__/__/

"FERMEZ LES YEUX"

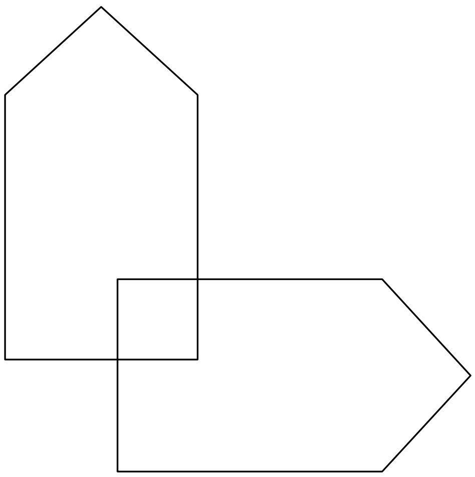

LE MMSE. CONSEILS POUR LA PASSATION DU TEST (GRECO)

Avertissement

La procédure pour chaque test doit être rigoureuse. L'évaluation neuropsychologique d'un patient peut donner des résultats très variables d'un examinateur à l'autre si chacun n'utilise pas la même procédure.
Chaque test a été conçu pour un domaine relativement précis d'exploration, en fonction des connaissances acquises et des modèles cognitifs validés, et doit se passer de manière standardisée pour permettre la comparaison de patients entre eux, au sein d'une population homogène, et d'un même patient à des moments différents afin de mettre en évidence d'éventuelles fluctuations inhérentes à certaines pathologies.

Conseils pour la passation du MMSE

Le Mini Mental Status Examination est un test d'évaluation cognitive globale. Il explore l'orientation dans le temps et dans l'espace, l'apprentissage, l'attention et le calcul, le rappel libre, le langage et les praxies constructives.
Pour être un outil valide d'évaluation cognitive, **sa passation a été standardisée par le GRECO** de la manière suivante :

Orientation

Pour tous les items, n'accepter que la réponse exacte. Cependant, lors des changements de saison ou de mois, ou pour l'étage, permettre au patient de corriger une réponse erronée, en lui demandant : " Etes-vous sûr ? ". Les seules tolérances admises concernent :

- Item 6 : lorsque le patient vient d'une autre ville, on peut se contenter de l'hôpital de la ville (car le nom de l'hôpital peut ne pas être connu du patient) ; si l'examen est réalisé en cabinet, demander : "Dans quel pays est situé ce département ? ".
- Item 8 : lorsque le nom du département et de la région sont identiques (par exemple : Nord), il faut alors demander : "Dans quel pays est situé ce département ? ". Chaque réponse juste vaut 1 point.

Si la réponse est fausse ou s'il n'y a pas de réponse, comptez 0. Accordez 10 secondes pour chaque réponse.

Apprentissage

Dire les 3 mots groupés, un par seconde, face au malade en articulant bien. Accorder 20 secondes pour la réponse. Compter 1 point pour chaque mot répété correctement au premier essai. Si le sujet ne répète pas les 3 mots au premier essai, les redonner jusqu'à ce qu'ils soient répétés correctement. En effet, l'épreuve de rappel ne peut être analysée que si les 3 mots ont été bien enregistrés. Maximum : 6 essais.

Attention et calcul

Item 14 à 18: il faut donner au sujet le maximum de chances, car il s'agit d'une épreuve difficile même pour des sujets témoins. Pour cela, on donnera la consigne suivante :
"Maintenant, je vais vous demander de compter en arrière de 7 en 7 à partir de 100 : combien font 100 - 7 ?"
Si la réponse est incorrecte, on permettra au patient une seconde réponse en lui demandant : « En êtes-vous sûr ? ». Si la réponse est inexacte, le point n'est pas accordé et on poursuit l'épreuve retranchant 7 de ce résultat sans le corriger et ainsi de suite pour les 5 soustractions.
Pour le mot à épeler à l'envers, noter le nombre de lettres dans l'ordre correct : ce chiffre ne doit pas figurer dans le score global. Accorder 10 secondes par réponse et arrêter l'épreuve à 3 erreurs.

Rappel

Item 11 à 13 : compter 1 point par réponse correcte.
Aucune tolérance n'est admise, puisque l'encodage a été contrôlé lors de l'enregistrement.

Langage

Item 22 : il faut montrer un crayon (et non un stylo ou un stylo à bille). Aucune réponse autre que crayon n'est admise.
Item 23 : aucune autre réponse que montre ou montre-bracelet n'est admise.
Item 24 : la phrase doit être prononcée à voix haute, bien distinctement face au malade. Ne compter 1 point que si la répétition est entièrement correcte.
Item 25 à 27 : compter 1 point par item correctement exécuté. Si le sujet s'arrête et demande ce qu'il doit faire, il ne faut pas répéter la consigne, mais dire : " Faîtes ce que je vous ai dit ".
Item 29 : compter 1 point si la phrase comprend un sujet et un verbe, sans tenir compte des fautes d'orthographe ou de syntaxe. Accorder 30 secondes. Compter 1 point par réponse correcte et accorder 10 secondes pour chaque réponse.

Praxies constructives

Compter 1 point si tous les angles sont présents et si les figures se coupent sur 2 côtés différents. On peut autoriser plusieurs essais et accorder un temps d'une minute.

QUESTIONNAIRE ADL (Activities of Daily Living)

Toilette : ❒

(lavabo, bain ou douche)

0 besoin d'aucune aide.

1 besoin d'aide pour une partie du corps (dos ou jambes).

2 besoin d'aide pour la toilette de plusieurs parties du corps, ou toilette impossible.

Habillage : ❒

(prend ses vêtements dans l'armoire ou les tiroirs, sous-vêtements et vêtements d'extérieur compris ; utilise boutons et fermeture éclair.)

0 besoin d'aucune aide.

1 besoin d'une aide uniquement pour lacer ses chaussures .

2 besoin d'aide pour prendre ses vêtements ou s'habiller, ou rester partiellement ou complètement déshabillé(e).

Aller aux W.C. :

(pour uriner ou déféquer, s'essuyer et se rhabiller)

0 besoin d'aucune aide (aide possible pour se rendre aux W.C. : canne, fauteuil roulant …, utilise lui-même le bassin).

1 besoin d'une aide.

2 Ne va pas aux W.C. ou n'utilise pas le bassin.

Locomotion : ❒

0 besoin d'aucune aide pour entrer et sortir du lit, s'asseoir ou se lever d'une chaise (peut utiliser un support comme une canne ou un déambulateur).

1 besoin d'une aide.

2 Ne quitte pas le lit.

Continence : ❒

0 contrôle complet des urines et des selles.

1 Accidents occasionnels.

2 Incontinence totale, nécessité de sondage ou de surveillance permanente.

Alimentation : ❒

0 besoin d'aucune aide.

1 besoin d'aide pour couper la viande ou beurrer le pain.

2 besoin d'aide complète ou alimentation artificielle.

TOTAL /12 ❒

QUESTIONNAIRE IADL (Instrumental Activities of Daily Living)

**A. Capacité à utiliser le téléphone :**		❐
	Je me sers du téléphone de ma propre initiative, cherche et compose les numéros etc….	1
	Je compose un petit nombre de numéros bien connus.	1
	Je réponds au téléphone mais n'appelle pas.	1
	Je suis incapable d'utiliser le téléphone.	0
**B. Faire les courses :**		❐
	Je fais toutes mes courses de façon indépendante.	1
	Je fais seulement les petits achats tout(e) seul(e).	0
	J'ai besoin d'être accompagné(e), quelle que soit la course.	0
	Je suis totalement incapable de faire les courses.	0
**C. Préparation des repas :**		❐
	Je prévois, prépare et sers des repas de façon indépendante.	1
	Je les prépare si on me fournit les ingrédients.	0
	Je suis capable de réchauffer des plats déjà préparés.	0
	J'ai besoin qu'on me prépare et serve les repas.	0
**D. Entretien de la maison :**		❐
	J'entretiens la maison seul(e) ou avec une aide occasionnelle, par exemple pour les gros travaux.	1
	Je ne fais que les petits travaux d'entretien quotidiens (vaisselle, lit, petit bricolage…)	1
	Je fais les petits travaux, mais sans parvenir à garder un niveau de propreté suffisant.	1
	J'ai besoin d'aide pour toutes les tâches d'entretien de la maison.	1
	Je ne peux pas participer du tout à l'entretien de la maison.	0
**E. Lessive :**		❐
	Je fais toute ma lessive personnelle ou la porte moi-même au pressing.	1
	Je lave les petites affaires.	1
	Toute la lessive doit être faite par d'autres.	0
**F. Moyen de transport :**		❐
	Je peux voyager seul(e) et de façon indépendante (par les transports en commun, ou avec ma propre voiture)	1
	Je peux me déplacer seul(e) en taxi, pas en autobus.	1
	Je peux prendre les transports en commun si je suis accompagné(e).	1
	Transport limité au taxi ou à la voiture, en étant accompagné(e).	0
	Je ne me déplace pas du tout.	0
**G. Responsabilité pour la prise des médicaments :**		❐
	Je m'occupe moi-même de la prise : dosage et horaire .	1
	Je peux les prendre de moi-même, s'ils sont préparés et dosés à l'avance.	0
	Je suis incapable de les prendre de moi-même.	0
**H. Capacité à gérer son budget :**		❐
	Je suis totalement autonome (gérer le budget, faire des chèques, payer des factures …).	1
	Je me débrouille pour les dépenses au jour le jour, mais j'ai besoin d'aide pour gérer mon budget à long terme (pour planifier les grosses dépenses).	1
	Je suis incapable de gérer l'argent nécessaire à payer mes dépenses au jour le jour.	0
**TOTAL /8**		❐

QUESTIONNAIRE GDS15 (Geriatric Depression Scale)

Nous allons maintenant vous poser quelques questions sur votre vision de la vie et sur votre moral. Merci de répondre par OUI ou par NON à chaque question. Si vous avez un doute, merci de précisez s'il tend plutôt vers un oui ou vers un non (ou : y a-t-il plus de bons jours ou de mauvais jours ?).

1.	Etes-vous fondamentalement satisfait(e) de la vie que vous menez?	Oui = 0<br>Non = 1	❒ 01
2.	Avez-vous abandonné un grand nombre d´activités et d'intérêts?	Oui = 1<br>Non = 0	❒ 02
3.	Est-ce que vous sentez un vide dans votre vie?	Oui = 1<br>Non = 0	❒ 03
4.	Vous ennuyez-vous souvent?	Oui = 1<br>Non = 0	❒ 04
5.	Avez-vous la plupart du temps un bon moral?	Oui = 0<br>Non = 1	❒ 05
6.	Craignez-vous qu'il vous arrive quelque chose de grave?	Oui = 1<br>Non = 0	❒ 06
7.	Etes-vous heureux(se) la plupart du temps?	Oui = 0<br>Non = 1	❒ 07
8.	Eprouvez-vous souvent un sentiment d'impuissance?	Oui = 1<br>Non = 0	❒ 08
9.	Préférez-vous rester chez vous au lieu de sortir pour faire de nouvelles activités?	Oui = 1<br>Non = 0	❒ 09
10.	Avez-vous l'impression d'avoir plus de problèmes de mémoire que la majorité des gens?	Oui = 1<br>Non = 0	❒ 10
11.	Pensez-vous qu'il est merveilleux de vivre à l'époque actuelle?	Oui = 0<br>Non = 1	❒ 11
12.	Vous sentez-vous plutôt inutile dans votre état actuel?	Oui = 1<br>Non = 0	❒ 12
13.	Vous sentez-vous plein(e) d'énergie?	Oui = 0<br>Non = 1	❒ 13
14.	Avez-vous l'impression que votre situation est désespérée?	Oui = 1<br>Non = 0	❒ 14
15.	Pensez-vous que la plupart des gens vivent mieux que vous?	Oui = 1<br>Non = 0	❒ 15

TOTAL ❒ 16

EORTC QLQ-C30 (version 3)

Nous nous intéressons à vous et à votre santé. Répondez vous-même à toutes les questions en entourant le chiffre qui correspond le mieux à votre situation. Il n'y a pas de "bonne" ou de "mauvaise" réponse. Ces informations sont strictement confidentielles.

Merci de préciser:
Vos initiales: /___/___/___/
Date de naissance (jour/mois/année): /___/___/___/
La date d`aujourd`hui (jour/mois/année): /___/___/___/

		Pas du tout	Un peu	Assez	Beaucoup
1.	Avez-vous des difficultés à faire certains efforts physiques pénibles comme porter un sac à provision chargé ou une valise?	1	2	3	4
2.	Avez-vous des difficultés à faire une longue promenade?	1	2	3	4
3.	Avez-vous des difficultés à faire un petit tour dehors?	1	2	3	4
4.	Etes-vous obligé(e) de rester au lit ou dans un fauteuil pendant la journée?	1	2	3	4
5.	Avez-vous besoin d'aide pour manger, vous habiller, faire votre toilette ou aller aux W.C.?	1	2	3	4

**Au cours de la semaine passée:**		Pas du tout	Un peu	Assez	Beaucoup
6.	Avez-vous été gêné(e) pour faire votre travail ou vos activités de tous les jours?	1	2	3	4
7.	Avez-vous été gêné(e) dans vos activités de loisirs?	1	2	3	4
8.	Avez-vous eu le souffle court?	1	2	3	4
9.	Avez-vous eu mal?	1	2	3	4
10.	Avez-vous eu besoin de repos?	1	2	3	4
11.	Avez-vous eu des difficultés pour dormir?	1	2	3	4
12.	Vous êtes-vous senti(e) faible?	1	2	3	4
13.	Avez-vous manqué d'appétit?	1	2	3	4
14.	Avez-vous eu des nausées (mal au coeur)?	1	2	3	4
15.	Avez-vous vomi?	1	2	3	4

Passez à la page suivante S.V.P.

**Au cours de la semaine passée:**		Pas du tout	Un peu	Assez	Beaucoup
16.	Avez-vous été constipé(e)?	1	2	3	4
17.	Avez-vous eu de la diarrhée?	1	2	3	4
18.	Etiez-vous fatigué(e)?	1	2	3	4
19.	Des douleurs ont-elles perturbé vos activités quotidiennes?	1	2	3	4
20.	Avez-vous eu des difficultés à vous concentrer sur certaines choses par exemple pour lire le journal ou regarder la télévision?	1	2	3	4
21.	Vous êtes-vous senti(e) tendu(e)?	1	2	3	4
22.	Vous êtes-vous fait du souci?	1	2	3	4
23.	Vous êtes-vous senti(e) irritable?	1	2	3	4

24.	Vous êtes-vous senti(e) déprimé(e)?	1	2	3	4
25.	Avez-vous eu des difficultés pour vous souvenir de certaines choses?	1	2	3	4
26.	Votre état physique ou votre traitement médical vous ont-ils gêné(e) dans votre vie familiale?	1	2	3	4
27.	Votre état physique ou votre traitement médical vous ont-ils gêné(e) dans vos activités sociales (par exemple sortir avec des amis, aller au cinéma...)?	1	2	3	4
28.	Votre état physique ou votre traitement médical vous ont-ils causé des problèmes financiers?	1	2	3	4

Pour les questions suivantes, veuillez répondre en entourant le chiffre entre 1 et 7 qui s'applique le mieux à votre situation

29. Comment évalueriez-vous votre état de santé au cours de la semaine passée?

1	2	3	4	5	6	7
Très mauvais						Excellent

30. Comment évalueriez-vous l'ensemble de votre qualité de vie au cours de la semaine passée?

1	2	3	4	5	6	7
Très mauvais						Excellent

© Copyright 1995 EORTC Study Group on Quality of Life. Tous droits réservés.
Version 3.0.

CUMULATIVE ILLNESS RATING SCALE FOR GERIATRICS CIRS(G)

Fiche d'enregistrement des comorbidités

(Miller, Paradis, and Reynolds 1991)

Instructions : Inscrivez toutes les comorbidités avec leur niveau de sévérité et leur caractère équilibré ou non. N'hésitez pas à inscrire tous les antécédents quelle que soit leur importance. ; les éléments significatifs seront mis en valeur par la cotation ultérieure. Incluez les antécédents chirurgicaux et médicaux, les problèmes fonctionnels tels que l'incontinence ou la constipation. Si une comorbidité est liée à la tumeur ou à son traitement, indiquez le en marquant "TR" après le diagnostic. N'oubliez pas le tabagisme et les médicaments (page 2). Mettez autant de détails que possible sur les médicaments, la posologie car cela sera utilisé pour coter les comorbidités. Prenez en compte les résultats de laboratoire

A compléter avant la mise en place du traitement

Coeur	Foie
Vasculaire	Rénal
Hématopoietique	Génitourinaire
Respiratoire	Musculo-squelettique/Téguments
Yeux, oreilles, nez, ORL	Neurologique
Partie supérieure du tube digestif	Endocrinologique/Metabolique et sein
Partie inférieure du tube digestif	Troubles psychiatriques
Tabagisme	

Médicaments en rapport avec les comorbidités et posologie
Commentairess :
**Note** : La cotation de la sévérité des comorbidités sera effectuée selon les critères suivants 0-Aucun problème ; 1-Problème actuel mais mineur ou problème significatif mais ancien ; 2-Handicap ou comorbidité modéré nécessitant un traitement de première ligne ; 3- Handicap ou comorbidité sévère/constant incontrolable / problèmes chroniques; 4-Extrèmement sévère/traitement immédiat nécessaire/insuffisance organique terminale/dégradation significative de la fonction.

ANNEXE 5 : DECLARATION D'HELSINKI

DECLARATION D'HELSINKI DE L'ASSOCIATION MEDICALE MONDIALE

Principes éthiques applicables aux recherches médicales sur des sujets humains

Adoptée par la 18e Assemblée générale, Helsinki, Juin 1964 et amendée par les
29e Assemblée générale, Tokyo, Octobre 1975
35e Assemblée générale, Venise, Octobre 1983
41e Assemblée générale, Hong Kong, Septembre 1989
48e Assemblée générale, Somerset West (Afrique du Sud), Octobre 1996
52e Assemblée générale, Edimbourg, Octobre 2000
l'Assemblée générale de l'AMM, Washington 2002 (addition d'une note explicative concernant le paragraphe 29) et
l'Assemblée générale de l'AMM, Tokyo 2004 (addition d'une note explicative concernant le paragraphe 30)

A. INTRODUCTION

1. La Déclaration d'Helsinki, élaborée par l'Association médicale mondiale, constitue une déclaration de principes éthiques dont l'objectif est de fournir des recommandations aux médecins et autres participants à la recherche médicale sur des êtres humains. Celle-ci comprend également les études réalisées sur des données à caractère personnel ou des échantillons biologiques non anonymes.

2. La mission du médecin est de promouvoir et de préserver la santé de l'être humain. Il exerce ce devoir dans la plénitude de son savoir et de sa conscience.

3. Le Serment de Genève de l'Association médicale mondiale lie le médecin dans les termes suivants : "La santé de mon patient sera mon premier souci" et le Code international d'éthique médicale énonce que "le médecin devra agir uniquement dans l'intérêt de son patient lorsqu'il lui procure des soins qui peuvent avoir pour conséquence un affaiblissement de sa condition physique ou mentale".

4. Les progrès de la médecine sont fondés sur des recherches qui, in fine, peuvent imposer de recourir à l'expérimentation humaine.

5. Dans la recherche médicale sur les sujets humains, les intérêts de la science et de la société ne doivent jamais prévaloir sur le bien-être du sujet.

6. L'objectif essentiel de la recherche médicale sur des sujets humains doit être l'amélioration des méthodes diagnostiques, thérapeutiques et de prévention, ainsi que la compréhension des causes et des mécanismes des maladies. Les méthodes diagnostiques, thérapeutiques et de prévention, même les plus éprouvées, doivent constamment être remises en question par des recherches portant sur leur efficacité, leur efficience et leur accessibilité.

7. Dans la recherche médicale comme dans la pratique médicale courante, la mise en œuvre de la plupart des méthodes diagnostiques, thérapeutiques et de prévention expose à des risques et à des contraintes.

8. La recherche médicale est soumise à des normes éthiques qui visent à garantir le respect de tous les êtres humains et la protection de leur santé et de leurs droits. Certaines catégories de sujets sont plus vulnérables que d'autres et appellent une protection adaptée. Les besoins spécifiques des sujets défavorisés au plan économique comme au plan médical doivent être identifiés. Une attention particulière doit être portée aux personnes qui ne sont pas en mesure de donner ou de refuser elles-mêmes leur consentement, à celles qui sont susceptibles de donner leur consentement sous la contrainte, à celles qui ne bénéficieront pas personnellement de la recherche et à celles pour lesquelles la recherche est conduite au cours d'un traitement.

9. L'investigateur doit être attentif aux dispositions éthiques, légales et réglementaires applicables à la recherche sur les sujets humains dans son propre pays ainsi qu'aux règles internationales applicables. Aucune disposition nationale d'ordre éthique, légal et réglementaire ne doit conduire à affaiblir ou supprimer les mesures protectrices énoncées dans la présente déclaration.

B. PRINCIPES FONDAMENTAUX APPLICABLES A TOUTE FORME DE RECHERCHE MEDICALE

10. Dans la recherche médicale, le devoir du médecin est de protéger la vie, la santé, la dignité et l'intimité de la personne.

11. La recherche médicale sur des êtres humains doit se conformer aux principes scientifiques généralement reconnus. Elle doit se fonder sur une connaissance approfondie de la littérature scientifique et des autres sources pertinentes d'information ainsi que sur une expérimentation appropriée réalisée en laboratoire et, le cas échéant, sur l'animal.

12. Des précautions particulières doivent entourer les recherches pouvant porter atteinte à l'environnement et le bien-être des animaux utilisés au cours des recherches doit être préservé.

13. La conception et l'exécution de chaque phase de l'expérimentation sur des sujets humains doivent être clairement définies dans un protocole expérimental. Ce protocole doit être soumis pour examen, commentaires, avis et, le cas échéant, pour approbation, à un comité d'éthique mis en place à cet effet. Ce comité doit être indépendant du promoteur, de l'investigateur ou de toute autre forme d'influence indue. Il doit respecter les lois et règlements en vigueur dans le pays où s'effectuent les recherches. Il a le droit de suivre le déroulement des études en cours. L'investigateur a l'obligation de fournir au comité des informations sur le déroulement de l'étude portant en particulier sur la survenue d'événements indésirables d'une certaine gravité. L'investigateur doit également communiquer au comité, pour examen, les informations relatives au financement, aux promoteurs, à toute appartenance à une ou des institutions, aux éventuels conflits d'intérêt ainsi qu'aux moyens d'inciter des personnes à participer à une recherche.

14. Le protocole de la recherche doit contenir une déclaration sur les implications éthiques de cette recherche. Il doit préciser que les principes énoncés dans la présente déclaration sont respectés.

15. Les études sur l'être humain doivent être conduites par des personnes scientifiquement qualifiées et sous le contrôle d'un médecin compétent. La responsabilité à l'égard d'un sujet inclus dans une recherche doit toujours incomber à une personne médicalement qualifiée et non au sujet, même consentant.

16. Toute étude doit être précédée d'une évaluation soigneuse du rapport entre d'une part, les risques et les contraintes et d'autre part, les avantages prévisibles pour le sujet ou d'autres personnes. Cela n'empêche pas la participation à des recherches médicales de volontaires sains. Le plan de toutes les études doit être accessible.

17. Un médecin ne doit entreprendre une étude que s'il estime que les risques sont correctement évalués et qu'ils peuvent être contrôlés de manière satisfaisante. Il doit être mis un terme à la recherche si les risques se révèlent l'emporter sur les bénéfices escomptés ou si des preuves consistantes de résultats positifs et bénéfiques sont apportées.

18. Une étude ne peut être réalisée que si l'importance de l'objectif recherché prévaut sur les contraintes et les risques encourus par le sujet. C'est particulièrement le cas lorsqu'il s'agit d'un volontaire sain.

19. Une recherche médicale sur des êtres humains n'est légitime que si les populations au sein desquelles elle est menée ont des chances réelles de bénéficier des résultats obtenus.

20. Les sujets se prêtant à des recherches médicales doivent être des volontaires informés des modalités de leur participation au projet de recherche.

21. Le droit du sujet à la protection de son intégrité doit toujours être respecté. Toutes précautions doivent être prises pour respecter la vie privée du sujet, la confidentialité des données le concernant et limiter les répercussions de l'étude sur son équilibre physique et psychologique.

22. Lors de toute étude, la personne se prêtant à la recherche doit être informée de manière appropriée des objectifs, méthodes, financement, conflits d'intérêts éventuels, appartenance de l'investigateur à une ou des institutions, bénéfices attendus ainsi que des risques potentiels de l'étude et des contraintes qui pourraient en résulter pour elle. Le sujet doit être informé qu'il a la faculté de ne pas participer à l'étude et qu'il est libre de revenir à tout moment sur son consentement sans crainte de préjudice. Après s'être assuré de la bonne compréhension par le sujet de l'information donnée, le médecin doit obtenir son consentement libre et éclairé, de préférence par écrit. Lorsque le consentement ne peut être obtenu sous forme écrite, la procédure de recueil doit être formellement explicitée et reposer sur l'intervention de témoins.

23. Lorsqu'il sollicite le consentement éclairé d'une personne à un projet de recherche, l'investigateur doit être particulièrement prudent si le sujet se trouve vis-à-vis de lui dans une situation de dépendance ou est exposé à donner son consentement sous une forme de contrainte. Il est alors souhaitable que le consentement soit sollicité par un médecin bien informé de l'étude mais n'y prenant pas part et non concerné par la relation sujet-investigateur.

24. Lorsque le sujet pressenti est juridiquement incapable, physiquement ou mentalement hors d'état de donner son consentement ou lorsqu'il s'agit d'un sujet mineur, l'investigateur doit obtenir le consentement éclairé du représentant légal en conformité avec le droit en vigueur. Ces personnes ne peuvent être inclues dans une étude que si celle-ci est indispensable à l'amélioration de la santé de la population à laquelle elles appartiennent et ne peut être réalisée sur des personnes aptes à donner un consentement.

25. Lorsque le sujet, bien que juridiquement incapable (un mineur par exemple), est cependant en mesure d'exprimer son accord à la participation à l'étude, l'investigateur doit obtenir que cet accord accompagne celui du représentant légal.

26. La recherche sur des personnes dont il est impossible d'obtenir le consentement éclairé, même sous forme de procuration ou d'expression préalable d'un accord, ne doit être conduite que si l'état physique ou mental qui fait obstacle à l'obtention de ce consentement est une des caractéristiques requises des sujets à inclure dans l'étude. Les raisons spécifiques d'inclure des sujets dans une étude en dépit de leur incapacité à donner un consentement éclairé doivent être exposées dans le protocole qui sera soumis au comité pour examen et approbation. Le protocole doit également préciser que le consentement du sujet ou de son représentant légal à maintenir sa participation à l'étude doit être obtenu le plus rapidement possible.

27. Les auteurs et les éditeurs de publications scientifiques ont des obligations d'ordre éthique. Lors de la publication des résultats d'une étude, les investigateurs doivent veiller à l'exactitude des résultats. Les résultats négatifs aussi bien que les résultats positifs doivent être publiés ou rendus accessibles. Le financement, l'appartenance à une ou des institutions et les éventuels conflits d'intérêt doivent être exposés dans les publications. Le compte-rendu d'une étude non conforme aux principes énoncés dans cette déclaration ne doit pas être accepté pour publication.

C. PRINCIPES APPLICABLES A LA RECHERCHE MEDICALE CONDUITE AU COURS D'UN TRAITEMENT

28. Le médecin ne peut mener une recherche médicale au cours d'un traitement que dans la mesure où cette recherche est justifiée par un possible intérêt diagnostique, thérapeutique ou de prévention. Quand la recherche est associée à des soins médicaux, les patients se prêtant à la recherche doivent bénéficier de règles supplémentaires de protection.

29. Les avantages, les risques, les contraintes et l'efficacité d'une nouvelle méthode doivent être évalués par comparaison avec les meilleures méthodes diagnostiques, thérapeutiques ou de prévention en usage. Cela n'exclut ni le recours au placebo ni l'absence d'intervention dans les études pour lesquelles il n'existe pas de méthode diagnostique, thérapeutique ou de prévention éprouvée.1

30. Tous les patients ayant participé à une étude doivent être assurés de bénéficier à son terme des moyens diagnostiques, thérapeutiques et de prévention dont l'étude aura montré la supériorité.2

31. Le médecin doit donner au patient une information complète sur les aspects des soins qui sont liés à des dispositions particulières du protocole de recherche. Le refus d'un patient de participer à une étude ne devra en aucun cas porter atteinte aux relations que le médecin entretient avec ce patient.

32. Lorsqu'au cours d'un traitement, les méthodes établies de prévention, de diagnostic ou de thérapeutique s'avèrent inexistantes ou insuffisamment efficaces, le médecin, avec le consentement éclairé du patient, doit pouvoir recourir à des méthodes non éprouvées ou nouvelles s'il juge que celles-ci offrent un espoir de sauver la vie, de rétablir la santé ou de soulager les souffrances du malade. Ces mesures doivent, dans toute la mesure du possible, faire l'objet d'une recherche destinée à évaluer leur sécurité et leur efficacité. Toute nouvelle information sera consignée et, le cas échéant, publiée. Les autres recommandations appropriées énoncées dans la présente déclaration s'appliquent.

1 **Note explicative concernant le paragraphe 29**

L'AMM note avec préoccupation que le paragraphe 29 de la Déclaration d'Helsinki (Octobre 2000) est l'objet d'interprétations diverses et de possibles malentendus. Elle réaffirme par ailleurs que les essais avec témoins sous placebo ne doivent être utilisés qu'avec de grandes précautions et, d'une façon générale, lorsqu'il n'existe pas de traitement éprouvé. Toutefois, même s'il existe un traitement éprouvé, les essais avec témoins sous placebo peuvent être éthiquement acceptables dans les conditions suivantes:

• lorsque, pour des raisons méthodologiques impérieuses et scientifiquement solides, il n'existe pas d'autres moyens qui permettent de déterminer l'efficacité ou l'innocuité d'une méthode prophylactique, diagnostique ou thérapeutique ; ou

• lorsqu'une méthode prophylactique, diagnostique ou thérapeutique est mise à l'essai pour une affection bénigne et que la participation à l'essai n'expose pas à des risques supplémentaires de dommages significatifs ou durables. Toutes les dispositions énoncées dans la DoH doivent être respectées, en particulier, la nécessité d'un examen éthique et scientifique approfondi.

2 **Note explicative concernant le paragraphe 30**

Par la présente, l'AMM réaffirme la nécessité d'identifier, lors de la planification d'une étude, l'accès post-étude pour les participants aux procédures prophylactiques, diagnostiques et thérapeutiques considérées comme bénéfiques par cette même étude ou un accès à d'autres soins appropriés. Les dispositions prises pour un tel accès ou pour d'autres soins doivent être décrites dans le protocole d'étude afin que le comité de révision éthique puisse étudier ces dispositions.

La déclaration d'Helsinki (Document 17.C) est un document officiel de l'Association médicale mondiale, représentante des médecins dans le monde. Adoptée en 1964 à Helsinki (Finlande), elle fut révisée en 1975 à Tokyo (Japon), en 1983 à Venise (Italie), en 1989 à Hong Kong, en 1996 à Somerset West (Afrique du Sud), en 2000 à Edimbourg (Ecosse), par l'Assemblée générale de l'AMM, Washington 2002 (addition d'une note explicative concernant le paragraphe 29), et par l'Assemblée générale de l'AMM, Tokyo 2004 (addition d'une note explicative concernant le paragraphe 30). 9.10.2004

ANNEXE 6 : CRITÈRES DE TOXICITÉ (NCI-CTC V3.0)

Common Terminology Criteria for Adverse Events v3.0 (CTCAE)
Publish Date: December 12, 2003

QUICK REFERENCE
The NCI Common Terminology Criteria for Adverse Events v3.0 is a descriptive terminology which can be utilized for Adverse Event (AE) reporting. A grading (severity) scale is provided for each AE term.

Grades
Grade refers to the severity of the AE. The CTCAE v3.0 displays Grades 1 through 5 with unique clinical descriptions of severity for each AE based on this general guideline:

Grade 1 Mild AE
Grade 2 Moderate AE
Grade 3 Severe AE
Grade 4 Life-threatening or disabling AE
Grade 5 Death related to AE

REMARK
A 'REMARK' is a clarification of an AE.

NAVIGATION NOTE
A 'NAVIGATION NOTE' indicates the location of an AE term within the CTCAE document. It lists signs/symptoms alphabetically and the CTCAE term will appear in the same CATEGORY unless the 'NAVIGATION NOTE' states differently

ADL:	Activities of Daily Living
ANC:	Absolute Neutrophil Count
AGC:	Absolute Granulocyte Count
BSA:	Body Surface Area
CHF:	Congestive Heart Failure
GVHD:	Graft-versus-Host-Disease
ICP:	Intracranial Pressure
TPN:	Total Parenteral Nutrition

		GRADE				
**Adverse Event**	**Short Name**	**1**	**2**	**3**	**4**	**5**
**AUDITORY / EAR**						
Tinnitus	Tinnitus	—	Tinnitus not interfering with ADL	Tinnitus interfering with ADL	Disabling	
Auditory/Ear – Other (Specify)	Auditory/Ear – Other (Specify)	Mild	Moderate	Severe	Life-threatening; disabling	Death
**BLOOD/ BONE MARROW**						
Bone marrow cellularity	Bone marrow cellularity	Mildly hypocellular or ≤25% reduction from normal cellularity for age	Moderately hypocellular or >25 – ≤50% reduction from normal cellularity for age	Severely hypocellular or >50 – ≤75% reduction cellularity from normal for age	—	Death
Hemoglobin	Hemoglobin	<LLN – 10.0 g/dL<br><LLN – 6.2 mmol/L<br><LLN – 100 g/L	<10.0 – 8.0 g/dL<br><6.2 – 4.9 mmol/L<br><100 – 80g/L	<8.0 – 6.5 gIdL<br><4.9 – 4.0 mmol/L<br><80 – 65 g/L	<6.5 g/dL<br><4.0 mmol/L<br><65 g/L	Death
Leukocytes (total WBC)	Leukocytes	<LLN – 3000/mm^3<br><LLN – 3.0x10^9/L	<3000–2000/mm^3<br><3.0 – 2.0x10^9/L	<2000–1000/mm^3<br><2.0 – 1.0x10^9/L	<1000/mm^3<br><1.0x10^9/L	Death
Lymphopenia	Lymphopenia	<LLN – 800/mm^3<br><LLN x0.8–10^9/L	<800– 500/mm^3<br><0.8–0.5x10^9/L	<500 – 200 mm^3<br><0.5 – 0.2x10^9/L	<200/mm^3<br><0.2x10^9/L	Death
Neutrophils/Granulocytes (ANC/AGC)	Neutrophils	<LLN – 1500/mm^3<br><LLN – 1.5 x 10^9 /L	<1500 – 1000/mm^3<br><1.5 – 1.0 x 10^9 /L	<1000 – 500/mm^3<br><1.0 – 0.5 x 10^9 /L	<500/mm^3<br><0.5 x 10^9 /L	Death
Platelets	Platelets	<LLN – 75,000/mm^3<br><LLN – 75 0 x 10^9 /L	<75,000 – 50,000/mm^3<br><75.0–50.0x10^9 /L	<50,000 – 25,000/mm^3<br><50.0 – 25.0x10^9/L	<25,000/mm^3<br><25.0 x 10^9 /L	Death
Blood/Bone Marrow – Other (Specify)	Blood – Other (Specify)	Mild	Moderate	Severe	Life-threatening; disabling	Death
**CARDIAC ARRHYTHMIA**						
Palpitations	Palpitations	Present	Present with associated symptoms (e.g., lightheadedness, shortness of breath)	—	—	—
REMARK: Grade palpitations only in the absence of a documented arrhythmia.						
Supraventricular and nodal arrhythmia	Supraventricular arrhythmia –	Asymptomatic, intervention not indicated	Non-urgent medical intervention indicated	Symptomatic and incompletely controlled medically or controlled with device (e.g., pacemaker)	Life-threatening (e.g., arrhythmia associated with CHF, hypotension, syncope, shock)	Death

		GRADE				
**Adverse Event**	**Short Name**	**1**	**2**	**3**	**4**	**5**
Select<br>– Atrial fibrillation<br>– Atrial flutter<br>– Atrial tachycardia/Paroxysmal Atrial Tachycardia<br>– Nodal/Junctional<br>– Sinus arrhythmia<br>– Sinus bradycardia<br>– Sinus tachycardia<br>– Supraventricular arrhythmia NOS<br>– Supraventricular extrasystoles (Premature Atrial Contractions; Premature Nodal/Junctional Contractions)<br>– Supraventricular tachycardia						
Vasovagal episode	Vasovagal episode	—	Present without loss of consciousness	Present with loss of consciousness	Life-threatening consequences	Death
Cardiac Arrhythmia – Other (Specify,_)	Cardiac Arrhythmia – Other (Specify,_)	Mild	Moderate	Severe	Life-threatening; disabling	Death
**CARDIAC GENERAL**						
Cardiac ischemia/infarction	Cardiac ischemia/infarction	Asymptomatic arterial narrowing without ischemia	Asymptomatic and testing suggesting ischemia; stable angina	Symptomatic and testing consistent with ischemia; unstable angina; intervention indicated	Acute myocardial infarction	Death
Cardiopulmonary arrest, cause unknown (non-fatal)	Cardiopulmonary arrest	—	—	—	Life-threatening	—
REMARK: Grade 4 (non-fatal) is the only appropriate grade. CTCAE provides three alternatives for reporting Death:<br>1. A CTCAE term associated with Grade 5.<br>2. A CTCAE 'Other (Specify)', within any CATEGORY.<br>3. Death not associated with CTCAE term – *Select* in the DEATH CATEGORY.						
Hypertension	Hypertension	Asymptomatic, transient (<24 hrs) increase by >20 mmHg (diastolic) or to >150/100 if previously WNL; intervention not indicated	Recurrent or persistent (>24 hrs) or symptomatic increase by >20 mmHg (diastolic) or to >150/100 if previously WNL; monotherapy may be indicated	Requiring more than one drug or more intensive therapy than previously	Life-threatening consequences (e.g., hypertensive crisis)	Death
Hypotension	Hypotension	Changes, intervention not indicated	Brief (<24 hrs) fluid replacement or other therapy; no physiologic consequences	Sustained (>24hrs) therapy, resolves without persisting physiologic consequences	Shock (e.g., acidemia; impairment of vital organ function)	Death
Left ventricular systolic dysfunction	Left ventricular systolic dysfunction	Asymptomatic, resting ejection fraction (EF) <60 – 50%; shortening fraction (SF) <30 – 24%	Asymptomatic, resting EF <50 – 40%; SF <24 – 15%	Symptomatic CHF responsive to intervention; EF <40 – 20% SF <15%	Refractory CHF or poorly controlled; EF <20%; intervention such as ventricular assist device, ventricular reduction surgery, or heart transplant indicated	Death
Pericardial effusion (non-malignant)	Pericardial effusion	Asymptomatic effusion	—	Effusion with physiologic consequences	Life-threatening consequences (e.g., tamponade); emergency intervention indicated	Death
Pericarditis	Pericarditis	Asymptomatic, ECG or physical exam (rub) changes consistent with pericarditis	Symptomatic pericarditis (e.g., chest pain)	Pericarditis with physiologic consequences (e.g., pericardial constriction)	Life-threatening consequences; emergency intervention indicated	Death
Cardiac General – Other (Specify, _)	Cardiac General – Other (Specify, _)	Mild	Moderate	Severe	Life-threatening; disabling	Death
**CONSTITUTIONAL SYMPTOMS**						
Fatigue (asthenia, lethargy, malaise)	Fatigue	Mild fatigue over baseline	Moderate or causing difficulty performing some ADL	Severe fatigue interfering with ADL	Disabling	—

		GRADE				
**Adverse Event**	**Short Name**	**1**	**2**	**3**	**4**	**5**
Fever (in the absence of neutropaenia, where neutropaenia is defined as ANC <1.0 x 10^9/L)	Fever	38.0 — 39.0°C	>39.0 — 40.0°C	>40.0°C for <24hrs	>40.0°C for >24hrs	Death
REMARK: The temperature measurements listed are oral or tympanic.						
Insomnia	Insomnia	Occasional difficulty sleeping, not interfering with function	Difficulty sleeping, interfering with function but not interfering with ADL	Frequent difficulty sleeping, interfering with ADL	Disabling	—
REMARK: If pain or other symptoms interfere with sleep, do NOT grade as insomnia. Grade primary event(s) causing insomnia.						
Sweating (diaphoresis)	Sweating	Mild and occasional	Frequent or drenching	—	—	—
Weight gain	Weight gain	5 – <10% of baseline	10 – <20% of baseline	≥ 20% of baseline	—	—
REMARK: Edema, depending on etiology, is graded in the CARDIAC GENERAL or LYMPHATICS CATEGORIES.						
Weight loss	Weight loss	5 to <10% from baseline; intervention not indicated	10 – <20% from baseline; nutritional support indicated	≥20% from baseline; tube feeding or TPN indicated	—	—
Constitutional Symptoms – Other (Specify)	Constitutional Symptoms – Other (Specify)	Mild	Moderate	Severe	Life-threatening; disabling	Death
**DERMATOLOGY/SKIN**						
Cheilitis	Cheilitis	Asymptomatic	Symptomatic, not interfering with ADL	Symptomatic, interfering with ADL	—	—
Dry skin	Dry skin	Asymptomatic	Symptomatic, not interfering with ADL	Interfering with ADL	—	—
Flushing	Flushing	Asymptomatic	Symptomatic	—	—	—
Hair Ioss/alopecia (scalp or body)	Alopecia	Thinning or patchy	Complete	—	—	—
Hyperpigmentation	Hyperpigmentation	Slight or Localized	Marked or generalized	—	—	—
Hypopigmentation	Hypopigmentation	Slight or localized	Marked or generalized	—	—	—
Induration/fibrosis (skin and subcutaneous tissue)	Induration	Increased density on palpation	Moderate impairment of function not interfering with ADL; marked increase in density and firmness on palpation **with *or* without minimal** retraction	Dysfunction interfering with ADL; very marked density, retraction or fixation	—	—
Nail changes	Nail changes	Discoloration; ridging (koilonychias); pitting	Partial or complete loss of nail(s); pain in nailbed(s)	Interfering with ADL	—	—
NAVIGATION NOTE: Petechiae is graded as Petechiae/purpura (hemorrhage/bleeding into skin or mucosa) in the HEMORRHAGE/BLEEDING CATEGORY						
Photosensitivity	Photosensitivity	Painless erythema	Painful erythema	Erythema with desquamation	Life-threatening; disabling	Death
Pruritus/itching	Pruritus	Mild or localized	Intense or widespread	Intense or widespread and interfering with ADL	—	—
Rash/ desquamation	Rash	Macular or papular eruption or erythema without associated symptoms	Macular or papular eruption or erythema with pruritus or other associated symptoms; localized desquamation or other lesions covering <50% of body surface area (BSA)	Severe, generalized erythroderma or macular, papular or vesicular eruption; desquamation covering ≥50% BSA	Generalized exfoliative, ulcerative, or bullous dermatitis	Death
REMARK: Rash/desquamation may be used for GVHD.						

		GRADE				
**Adverse Event**	**Short Name**	**1**	**2**	**3**	**4**	**5**
Rash: acne/acneiform	Acne	Intervention not indicated	Intervention indicated	Associated with pain, disfigurement, ulceration, or desquamation	—	Death
Rash: erythema multiforme (e.g., Stevens-Johnson syndrome, toxic epidermal necrolysis)	Erythema multiforme	—	Scattered, but not generalized eruption	Severe (e.g., generalized rash or painful stomatitis); IV fluids, tube feedings, or TPN indicated	Life-threatening; disabling	Death
Urticaria (hives, welts, wheals)	Urticaria	Intervention not indicated	Intervention indicated for <24 hrs	Intervention indicated for ≥24 hrs	—	—
Dermatology/Skin – Other	Dermatology – Other Mild	Moderate	Severe	Life-threatening; disabling	Death	
**ENDOCRINE**						
Hot flashes/flushes	Hot flashes	Mild	Moderate	Interfering with ADL	—	—
Endocrine - Other (Specify', _)	Endocrine – Other (Specify)	Mild	Moderate	Severe	Life-threatening; disabling	Death
**GASTROINTESTINAL**						
NAVIGATION NOTE: Abdominal pain or cramping is graded as Pain – *Select* in the PAIN CATEGORY.						
Abdominal Pain	Abdominal Pain	Mild	Moderate	Severe	—	—
Anorexia	Anorexia	Loss of appetite without alteration in eating habits	Oral intake altered without significant weight loss or malnutrition; oral nutritional supplements indicated	Associated with significant weight loss or malnutrition (e.g., inadequate oral caloric and/or fluid intake); IV fluids, tube feedings or TPN indicated	Life-threatening consequences	Death
Ascites (non-malignant)	Ascites	Asymptomatic	Symptomatic, medical intervention indicated	Symptomatic, invasive procedure indicated	Life-threatening consequences	Death
REMARK: Ascites (non-malignant) refers to documented non-malignant ascites or unknown etiology, but unlikely malignant, and includes chylous ascites.						
Colitis	Colitis	Asymptomatic, pathologic or radiographic findings only	Abdominal pain; mucus or blood in stool	Abdominal pain, fever, change in bowel habits with ileus; peritoneal signs	Life-threatening consequences (e.g., perforation, bleeding, ischemia, necrosis, toxic megacolon)	Death
Constipation	Constipation	Occasional or intermittent symptoms; occasional use of stool softeners, laxatives, dietary modification, or enema	Persistent symptoms with regular use of laxatives or enemas indicated	Symptoms interfering with ADL; constipation with manual evacuation indicated	Life-threatening consequences (e.g., obstruction, toxic megacolon)	Death
Dehydration	Dehydration	Increased oral fluids indicated; dry mucous membranes; diminished skin turgor	IV fluids indicated <24 hrs	IV fluids indicated ≥24 hrs	Life-threatening consequences (e.g., hemodynamic collapse)	Death
Diarrhoea	Diarrhoea	Increase of <4 stools per day over baseline; mild increase in ostomy output compared to baseline	Increase of 4 - 6 stools per day over baseline; IV fluids indicated <24hrs; moderate increase in ostomy output compared to baseline; not interfering with ADL	Increase of ≥7 stools per day over baseline; incontinence; IV fluids ≥24 hrs; hospitalization; severe increase in ostomy output compared to baseline; interfering with ADL	Life-threatening consequences (e.g., hemodynamic collapse)	Death
REMARK: Diarrhoea includes diarrhoea of small bowel or colonic origin, and/or ostomy diarrhoea.						
Distension/bloating abdominal	Distension	Asymptomatic	Symptomatic, but not interfering with GI function	Symptomatic, interfering with GI function	—	—

		GRADE				
**Adverse Event**	**Short Name**	**1**	**2**	**3**	**4**	**5**
Dry mouth/salivary gland (xerostomia)	Dry mouth	Symptomatic (dry or thick saliva) without significant dietary alteration; unstimulated saliva flow >0.2 ml/min	Symptomatic and significant oral intake alteration (e.g., copious water, other lubricants, diet limited to purees and/or soft, moist foods); unstimulated saliva 0.1 to 0.2 ml/min	Symptoms leading to inability to adequately aliment orally; IV fluids, tube feedings, or TPN indicated; unstimulated saliva <0.1 ml/min	—	—
REMARK: Dry mouth/salivary gland (xerostomia) includes descriptions of grade using both subjective and objective assessment parameters. Record this event consistently throughout a patient's participation on study. If salivary flow measurements are used for initial assessment, subsequent assessments must use salivary flow.						
Flatulence	Flatulence	Mild	Moderate	—	—	—
Gastritis (including bile reflux gastritis)	Gastritis	Asymptomatic radiographic or endoscopic findings only	Symptomatic; altered gastric function (e.g., inadequate oral caloric or fluid intake); IV fluids indicated <24 hrs	Symptomatic and severely altered gastric function (e.g., inadequate oral caloric or fluid intake); IV fluids, tube feedings, or TPN indicated ≥24 hrs	Life-threatening consequences; operative intervention requiring complete organ resection (e.g., gastrectomy)	Death
Heartburn/ dyspepsia	Heartburn	Mild	Moderate	Severe	—	—
Ileus, GI (functional obstruction of bowel, i.e., neuroconstipation)	Ileus	Asymptomatic, radiographic findings only	Symptomatic; altered GI function (e.g., altered dietary habits); IV fluids indicated <24 hrs	Symptomatic and severely altered GI function; IV fluids, tube feeding, or TPN indicated ≥24 hrs	Life-threatening consequences	Death
REMARK: Ilieus, GI is to be used for altered upper or lower GI function (e.g., delayed gastric or colonic emptying).						
Mucositis/stomatitis (clinical exam)<br>— Select<br>— Anus<br>— Esophagus<br>— Large bowel<br>— Larynx<br>— Oral cavity<br>— Pharynx<br>— Rectum<br>— Small bowel<br>— Stomach<br>— Trachea	Mucositis (clinical exam)<br>— *Select*	Erythema of the mucosa	Patchy ulcerations or pseudomembranes	Confluent ulcerations or pseudomembranes; bleeding with minor trauma	Tissue necrosis; significant spontaneous bleeding; life-threatening consequences	Death
REMARK: Mucositis/stomatitis (functional/symptomatic) may be used for mucositis of the upper aero-digestive tract caused by radiation, agents, or GVHD.						
Mucositis/stomatitis (functional/symptomtic)<br>Select:<br>— Anus<br>— Esophagus<br>— Large bowel<br>— Larynx<br>— Oral cavity<br>— Pharynx<br>— Rectum<br>— Small bowel<br>— Stomach<br>— Trachea	Mucositis (functional/ symptomatic) — *Select*	Upper aerodiqestive tract sites: Minimal symptoms,normal diet; minimal respiratory symptoms but not interfering with function<br><br>Lower GI sites: Minimal discomfort, intervention not indicated	Upper aerodiqestive tract sites: Symptomatic but can eat and swallow modified diet; respiratory symptoms interfering with function but not interfering with ADL<br><br>Lower GI sites: Symptomatic, medical intervention indicated but not interfering with ADL	Upper aerodiqestive tract ites: Symptomatic and unable to adequately aliment or hydrate orally; respiratory symptoms interfering with ADL<br><br>Lower GI sites: Stool incontinence or other symptoms interfering with ADL	Symptoms associated with life-threatening consequences	Death
Nausea	Nausea Vomiting.	Loss of appetite without alteration in eating habits	Oral intake decreased without significant weight loss, dehydration or malnutrition; IV fluids indicated <24 hrs	Inadequate oral caloric or fluid intake; IV fluids, tube feedings, or TPN indicated ≥24 hrs	Life-threatening consequences	Death

		GRADE				
**Adverse Event**	**Short Name**	**1**	**2**	**3**	**4**	**5**
Obstruction, GI<br>– *Select*<br>– Cecum<br>– Colon<br>– Duodenum<br>– Esophagus<br>– Gallbladder<br>– Ileum<br>– Jejunum<br>– Rectum<br>– Small bowel NOS<br>– Stoma<br>– Stomach	Obstruction, GI – Select	Asymptomatic radiographic findings only	Symptomatic; altered GI function (e.g., altered dietary habits, vomiting, diarrhoea, or GI fluid loss);<br>IV fluids indicated <24 hrs	Symptomatic and severely altered GI function (e.g., altered dietary habits, vomiting, diarrhoea, or GI fluid loss); IV fluids, tube feedings, or TPN indicated ≥24 hrs; operative intervention indicated	Life-threatening consequences; operative intervention requiring complete organ resection (e.g., total colectomy)	Death
Taste alteration (dysgeusia)	Taste alteration	Altered taste but no change in diet	Altered taste with change in diet (e.g., oral supplements); noxious or unpleasant taste; loss of taste	—	—	—
Ulcer, GI<br>– Select.<br>– Anus<br>– Cecum<br>– Colon<br>– Duodenum<br>– Esophagus<br>– Ileum<br>– Jejunum<br>– Rectum<br>– Small bowel NOS<br>– Stoma<br>– Stomach	Ulcer, GI – *Select*	Asymptomatic, radiographic or endoscopic findings only	Symptomatic; altered GI function (e.g., altered<br>dietary habits, oral supplements); IV fluids<br>indicated <24 hrs	Symptomatic and severely altered GI function (e.g., inadequate<br>oral caloric or fluid intake); IV fluids, tube<br>feedings, or TPN indicated ≥24 hrs	Life-threatening consequences	Death
Vomiting	Vomiting	1 episode in 24 hrs	2 – 5 episodes in 24 hrs; IV fluids indicated <24 hrs	≥6 episodes in 24 hrs; IV fluids, or TPN indicated ≥24 hrs	Life-threatening consequences	Death
Gastrointestinal – Other (Specify,)	GI – Other (Specify)	Mild	Moderate	Severe	Life-threatening; disabling	Death
**HEMORRHAGE/BLEEDING**						
Hematoma	Hematoma	Minimal symptoms, invasive intervention not indicated	Minimally invasive evacuation or aspiration indicated	Transfusion, interventional radiology, or operative intervention indicated	Life-threatening consequences; major urgent intervention indicated	Death
REMARK: Hematoma refers to extravasation at wound or operative site or secondary to other intervention. Transfusion implies pRBC.						
Hemorrhage, CNS	CNS hemorrhage	Asymptomatic, radiographic findings only	Medical intervention indicated	Ventriculostomy, ICP monitoring, intraventricular thrombolysis, or operative intervention indicated	Life-threatening consequences; neurologic deficit or disability	Death

		GRADE				
**Adverse Event**	**Short Name**	**1**	**2**	**3**	**4**	**5**
Hemorrhage, GI<br>– *Select.*<br>– Abdomen NOS<br>– Anus<br>– Biliary tree<br>– Cecum/appendix<br>– Colon<br>– Duodenum<br>– Esophagus<br>– Ileum<br>– Jejunum<br>– Liver<br>– Lower GI NOS<br>– Oral cavity<br>– Pancreas<br>– Peritoneal cavity<br>– Rectum<br>– Stoma<br>– Stomach<br>– Upper GI NOS<br>– Varices (esophageal)<br>– Varices (rectal)	Hemorrhage, GI – Select	Mild, intervention (other than iron supplements) not indicated	Symptomatic and medical intervention or minor cauterization indicated	Transfusion, interventional radiology, endoscopic, or operative intervention indicated; radiation therapy (i.e., hemostasis of bleeding site)	Life-threatening consequences; major urgent intervention indicated	Death
REMARK: Transfusion implies pRBC.						
Hemorrhage, pulmonary/ upper respiratory<br><br>– Nose	Hemorrhage pulmonary<br>– *Select*	Mild, intervention not indicated	Symptomatic and medical intervention indicated	Transfusion, interventional radiology, endoscopic, or operative intervention indicated; radiation therapy (i.e., hemostasis of bleeding site)	Life-threatening consequences; major urgent intervention indicated	Death
REMARK: Transfusion implies pRBC.						
Petechiae/purpura (hemorrhage/bleeding into skin or mucosa)	Petechiae	Few petechiae	Moderate petechiae; purpura	Generalized petechiae or purpura	—	—
NAVIGATION NOTE: Vitreous hemorrhage is graded in the OCULAR/VISUAL CATEGORY						
Hemorrhage/ Bleeding – Other (Specify,_)	Hemorrhage – Other (Specify,_)	Mild without transfusion	—	Transfusion indicated	Catastrophic bleeding, requiring major non-elective intervention	Death
**HEPATOBILIARY/PANCREAS**						
Liver dysfunction/failure (clinical)	Liver dysfunction	—	Jaundice	Asterixis	Encephalopathy or coma	Death
REMARK: Jaundice is not an AE, but occurs when the liver is not working properly or when a bile duct is blocked. It is graded as a result of liver dysfunction/failure or elevated bilirubin						
Pancreatitis	Pancreatitis	Asymptomatic, enzyme elevation and/or radiographic findings	Symptomatic, medical intervention indicated	Interventional radiology or operative intervention indicated	Life-threatening consequences (e.g., circulatory failure, hemorrhage, sepsis)	Death
Cholecystitis	Cholecystitis	Asymptomatic, radiographic findings only	Symptomatic, medical intervention indicated	Interventional radiology, endoscopic, or operative intervention indicated	Life-threatening consequences (e.g., sepsis or perforation)	Death
Hepatobiliary /Pancreas –Other (Specify,)	Hepatobiliary – Other (Specify)	Mild	Moderate	Severe	Life-threatening; disabling	Death
**INFECTION**						

		GRADE				
**Adverse Event**	**Short Name**	**1**	**2**	**3**	**4**	**5**
Colitis, infectious (e.g., Clostridium difficile)	Colitis, infectious	Asymptomatic, pathologic or radiographic findings only	Abdominal pain with mucus and/or blood in stool	IV antibiotics or TPN indicated	Life-threatening consequences (e.g., perforation, bleeding, ischemia, necrosis or toxic megacolon); operative resection or diversion indicated	Death
Febrile neutropaenia (fever of unknown origin without clinically or microbiologically documented infection) (ANC <1.0 x 10^9/L, fever ≥38.5°C)	Febrile neutropaenia	—	—	Present	Life-threatening consequences (e.g., septic shock, hypotension, acidosis, necrosis)	Death
Infection (documented clinically or microbiologically) with Grade 3 or 4 neutrophils (ANC <1.0 x 10^9/L) – *Select*	Infection (documented clinically) – *Select*	—	Localized, local intervention indicated	IV antibiotic, antifungal, or antiviral intervention indicated; interventional radiology or operative intervention indicated	Life-threatening consequences (e.g., septic shock, hypotension, acidosis, necrosis)	Death
REMARK: Fever with Grade 3 or 4 neutrophils in the absence of documented infection is graded as Febrile neutropaenia (fever of unknown origin without clinically or microbiologically documented infection).						
Infection with normal ANC or Grade 1 or 2 neutrophils – Select	Infection with normal ANC – *Select*	—	Localized, local intervention indicated	IV antibiotic, antifungal, or antiviral intervention indicated; interventional radiology or operative intervention indicated	Life-threatening consequences (e.g., septic shock, hypotension, acidosis, necrosis)	Death
Infection with unknown ANC – *Select* Se/ect'AEs appear at the end of the CATEGORY.	Infection with unknown ANC – *Select*	—	Localized, local intervention indicated	IV antibiotic, antifungal, or antiviral intervention indicated; interventional radiology or operative intervention indicated	Life-threatening consequences (e.g., septic shock, hypotension, acidosis, necrosis)	Death
REMARK: Infection with unknown ANC – *Select* is to be used in the rare case when ANC is unknown.						
Opportunistic infection associated with ≥Grade 2 Lymphopenia	Opportunistic infection	—	Localized, local intervention indicated	IV antibiotic, antifungal, or antiviral intervention indicated; interventional radiology or operative intervention indicated	Life-threatening consequences (e.g., septic shock, hypotension, acidosis, necrosis)	Death
Viral hepatitis	Viral hepatitis	Present; transaminases and liver function normal	Transaminases abnormal, liver function normal	Symptomatic Iiver dysfunction; fibrosis by biopsy; compensated cirrhosis	Decompensated liver function (e.g., ascites, coagulopathy, encephalopathy, coma)	Death
REMARK: Non-viral hepatitis is graded as Infection – Select.						
Infection – Other (Specify)	Infection – Other (Specify)	Mild	Moderate	Severe	Life-threatening; disabling	Death
**LYMPHATICS**						

		GRADE				
**Adverse Event**	**Short Name**	**1**	**2**	**3**	**4**	**5**
Dermal change lymphedema, phlebolymphedema	Dermal change	Trace thickening or faint discoloration	Marked discoloration; leathery skin texture; papillary formation	—	—	—
REMARK: Dermal change lymphedema, phlebolymphedema refers to changes due to venous stasis.						
Edema: head and neck	Edema: head and neck	Localized to dependent areas, no disability or functional impairment	Localized facial or neck edema with functional impairment	Generalized facial or neck edema with functional impairment (e.g., difficulty in turning neck or opening mouth compared to baseline)	Severe with ulceration or cerebral edema; tracheotomy or feeding tube indicated	Death
Edema: limb	Edema: limb	5 – 10% inter-limb discrepancy in volume or circumference at point of greatest visible difference; swelling or obscuration of anatomic architecture on close inspection; pitting edema	>10 – 30% inter-limb discrepancy in volume or circumference at point of greatest visible difference; readily apparent obscuration of anatomic architecture; obliteration of skin folds; readily apparent deviation from normal anatomic contour	>30% inter-limb discrepancy in volume; lymphorrhea; gross deviation from normal anatomic contour, interfering with ADL	Progression to malignancy (i.e., lymphangiosarcoma); amputation indicated; disabling	Death
Edema: trunk/genital	Edema: trunk/genital	Swelling or obscuration of anatomic architecture on close inspection; pitting edema	Readily apparent obscuration of anatomic architecture; obliteration of skin folds; readily apparent deviation from normal anatomic contour	Lymphorrhea; interfering with ADL; gross deviation from normal anatomic contour	Progression to malignancy (i.e., lymphangiosarcoma); disabling	Death
Edema: viscera	Edema: viscera	Asymptomatic; clinical or radiographic findings only	Symptomatic; intervention indicated	Symptomatic and unable to aliment adequately orally; interventional radiology or operative intervention indicated	Life threatening consequences	Death
Lymphatics – Other (Specify, _)	Lymphatics – Other (Specify, _)	Mild	Moderate	Severe	Life-threatening; disabling	Death
**METABOLIC/ LABORATORY**						
Alkaline phosphatase	Alkaline phosphatase	>ULN - 2.5 x ULN	>2.5 - 5.0 x ULN	>5.0 - 20.0 x ULN	>20.0 x ULN	-
ALT, SGPT (serum glutamic pyruvic transaminase)	ALT	>ULN - 2.5 x ULN	>2.5 - 5.0 x ULN	>5.0 - 20.0 x ULN	>20.0 x ULN	-
AST, SGOT (serum glutamic oxaloacetic transaminase)	AST	>ULN - 2.5 x ULN	>2.5 - 5.0 x ULN	>5.0 - 20.0 x ULN	>20.0 x ULN	-
Bilirubin (hyperbilirubinemia)	Bilirubin	>ULN - 1.5 x ULN	>1.5 - 3.0 x ULN	>3.0 - 10.0 x ULN	>10.0 x ULN	-
REMARK: Jaundice is not an AE, but may be a manifestation of liver dysfunction/failure or elevated bilirubin. If jaundice is associated with elevated bilirubin, grade bilirubin.						
Calcium, serum-low (hypocalcemia)<br><br>Ionized calcium:	Hypocalcemia	<LLN - 8.0 mg/dL<br><LLN - 2.0 mmol/L<br><br><LLN - 1.0 mmol/L	<8.0 - 7.0 mg/dL<br><2.0 - 1.75 mmol/L<br><br><1.0 - 0.9 mmol/L	<7.0 - 6.0 mg/dL<br><1.75 - 1.5 mmo/L<br><br><0.9 - 0.8 mmol/L	<6.0 mg/dL<br><1.5 mmol/L<br><br><0.8 mmol/L	Death
REMARK: Calcium tan be falsely low if hypoalbuminemia is present. Serum albumin is <4.0 g/dL, hypocalcemia is reported after the following corrective calculation has been performed: Corrected Calcium (mg/dL) = Total Calcium (mg/dL) - 0.8 [Albumin (g/dL) - 4]. Alternatively, direct measurement of ionized calcium is the definitive method to diagnose metabolically relevant alterations in serum calcium.						

		GRADE				
**Adverse Event**	**Short Name**	**1**	**2**	**3**	**4**	**5**
Calcium, serum-high (hypercalcemia)<br><br>Ionized calcium:	Hypercalcemia	>ULN - 1t5 mg/dL<br>>ULN - 2.9 mmol/L<br><br>>ULN - 1.5 mmol/L	>11.5 - 12.5 mg/dL<br>>2.9 - 3.1 mmo/L<br><br>>1.5 - 1.6 mmol/L	>12.5 - 13.5 mg/dL<br>>3.1 - 3.4 mmol/L<br><br>>1.6 - 1.8 mmol/L	>13.5 mg/dL<br>>3.4 mmol/L<br><br>>1.8 mmol/L	Death
Cholesterol, serum-high (hypercholesteremia	Cholesterol	>ULN - 300 mg/dL<br>>ULN - 7.75mmo/L	>300 - 400 mg/dL<br>>7.75 - 10.34 mmol/L	>400 - 500 mg/dL<br>>10.34 - 12.92mmol/L	>500 mg/dL<br>>12.92 mmol/L	Death
CPK (creatine phosphokinase)	CPK	>ULN - 2.5 x ULN	>2.5 x ULN - 5 x ULN	>5 x ULN - 10 x ULN	>10 x ULN	Death
Creatinine	Creatinine	>ULN - 1.5 x ULN	>1.5 - 3.0 x ULN	>3.0 - 6.0 x ULN	>6.0 x ULN	Death
Phosphate, serum-low (hypophosphatemia)	Hypophosphatemia	<LLN - 2.5 mg/dL<br><LLN - 0.8 mmol/L	<2.5 - 2.0 mg/dL<br><0.8 - 0.6 mmol/L	<2.0 - 1.0 mg/dL<br><0.6 - 0.3 mmol/L	<1.0 mg/dL<br><0.3 mmol/L	Death
Glomerular filtration rate ALSO CONSIDER: Creatinine.	GFR	<75 - 50% LLN	<50 - 25% LLN	<25% LLN, chronic dialysis not indicated	Chronic dialysis or renaltransplant indicated	Death
Potassium, serum-high (hyperkalemia)	Hyperkalemia	>ULN - 5.5 mmol/L	>5.5 - 6.0 mmol/L	>6.0 - 7.0 mmol/L	>7.0 mmol/L	Death
Potassium, serum-Iow (hypokalemia)	Hypokalemia	<LLN – 3.0 mmol/L	—	<3.0 – 2.5 mmol/L	<2.5 mmol/L	Death
Sodium, serum-Iow (hyponatremia)	Hyponatremia	<LLN – 130 mmol/L	—	<130 – 120 mmol/L	<120 mmol/L	Death
Triglyceride, serum-high (hypertriglyceri-demia)	Hypertriglyceridemia	>ULN – 2.5 x ULN	>2.5 – 5.0 x ULN	>5.0 – 10 x ULN	>10 x ULN	Death
Uric acid, serum-high (hyperuricemia)	Hyperuricemia	>ULN – 10 mg/dL ≤0.59 mmol/L without physiologic consequences	—	>ULN – 10 mg/dL ≤0.59 mmol/L with physiologic consequences	>10 mg/dL<br>>0.59 mmol/L	Death
Metabolic/Laboratory –Other (Specify)	Metabolic/Lab – Other (Specify)	Mild	Moderate	Severe	Life-threatening; disabling	Death
MUSCULOSKELETAL/SOFT TISSUE						
Joint-effusion	Joint-effusion	Asymptomatic, clinical or radiographic findings only	Symptomatic; interfering with function but not interfering with ADL	Symptomatic and interfering with ADL	Disabling	Death
Joint-function	Joint-function	Stiffness interfering with athletic activity; ≤25% Ioss of range of motion (ROM)	Stiffness interfering with function but not interfering with ADL; >25 – 50% decrease in ROM	Stiffness interfering with ADL; >50 – 75% decrease in ROM	Fixed or non-functional joint (arthrodesis); >75% decrease in ROM	—
Musculoskeletal/ Soft Tissue — Other (Specify)	Musculoskeletal — Other (Specify)	Mild	Moderate	Severe	Life-threatening; disabling	Death
Myalgia	Myalgia	Mild	Moderate	Severe	Disabling	—
**NEUROLOGY**						
Cognitive disturbance	Cognitive disturbance	Mild cognitive disability; not interfering with work/school/life performance; specialized educational services/devices not indicated	Moderate cognitive disability; interfering with work/school/life performance but capable of independent living; specialized resources on part-time basis indicated	Severe cognitive disability; significant impairment of work/school/Iife performance	Unable to perform ADL; full-time specialized resources or institutionalization indicated	Death
REMARK: Cognitive disturbance may be used for Attention Deficit Disorder (ADD).						
Confusion	Confusion	Transient confusion, disorientation, or attention deficit	Confusion, disorientation, or attention deficit interfering with function, but not interfering with ADL	Confusion or delirium interfering with ADL	Harmful to others or self; hospitalization indicated	Death
REMARK: Attention Deficit Disorder (ADD) is graded as Cognitive disturbance.						

		GRADE				
**Adverse Event**	**Short Name**	**1**	**2**	**3**	**4**	**5**
Dizziness	Dizziness	With head movements or nystagmus only; not interfering with function	Interfering with function, but not interfering with ADL	Interfering with ADL	Disabling	Death
REMARK: Dizziness includes disequilibrium, lightheadedness, and vertigo.						
Memory impairment	Memory impairment	Memory impairment not interfering with function	Memory impairment interfering with function, but not interfering with ADL	Memory impairment interfering with ADL	Amnesia	—
Mood alteration – *Select* – Agitation – Anxiety – Depression – Euphoria	Mood alteration – *Select*	Mild mood alteration not interfering with function	Moderate mood alteration interfering with function, but not interfering with ADL; medication indicated	Severe mood alteration interfering with ADL	Suicidal ideation; danger to self or others	Death
Neuropathy: cranial – Select – CN I Smell – CN II Vision – CN III Pupil, upper eyelid, extra ocular movements – CN IV Downward, inward movement of eye – CN V Motor- jaw muscles; Sensory-facial – CN VI Lateral deviation of eye – CN VII Motor-face; Sensory-taste –CN VIII Hearing and balance – CN IX Motor-pharynx; Sensory-ear, pharynx, tongue – CN X Motor-palate; pharynx, larynx – CN XI Motor-stemomastoid and trapezius – CN XII Motor-tongue	Neuropathy: cranial	Asymptomatic, detected on exam/testing only	Symptomatic, not interfering with ADL	Symptomatic, interfering with ADL	Life-threatening; disabling	Death
Neuropathy: motor	Neuropathy-motor	Asymptomatic, weakness on exam/testing only	Symptomatic weakness interfering with function, but not interfering with ADL	Weakness interfering with ADL; bracing or assistance to walk (e.g., cane or walker) indicated	Life-threatening; disabling (e.g., paralysis)	Death
Neuropathy: sensory	Neuropathy-sensory	Asymptomatic; loss of deep tendon reflexes or paresthesia (including tingling) but not interfering with function	Sensory alteration or paresthesia (including tingling), interfering with function, but not interfering with ADL	Sensory alteration or paresthesia interfering with ADL	Disabling	Death
REMARK: Cranial nerve sensory neuropathy is graded as Neuropathy: cranial – *Select.*						
Seizure	Seizure	—	One brief generalized seizure; seizure(s) wellcontrolled by anticonvulsants or infrequent focal motor seizures not interfering with ADL	Seizures in which consciousness is altered; poorly controlled seizure disorder, with breakthrough generalized seizures despite medical intervention	Seizures of any kind which are prolonged, repetitive, or difficult to control (e.g., status epilepticus, intractable epilepsy)	Death
Somnolence/ depressed level of consciousness	Somnolence	—	Somnolence or sedation interfering with function, but- not interfering-with ADL	Obtundation or stupor; difficult to arouse; interfering with- ADL	Coma	Death
Syncope (fainting)	Syncope (fainting)	—	—	Present	Life-threatening consequences	Death
NAVIGATION NOTE: Taste alteration (CN VII, IX) is graded as Taste alteration (dysgeusia) in the GASTROINTESTINAL CATEGORY.						
Neurology - Other (Specify,)	Neurology - Other (Specify)	Mild	Moderate	Severe	Life-threatening; disabling	Death
**OCULAR/VISUAL**						

		GRADE				
**Adverse Event**	**Short Name**	**1**	**2**	**3**	**4**	**5**
Dry eye syndrome	Dry eye	Mild, intervention not indicated	Symptomatic, interfering with function but not interfering with ADL; medical intervention indicated	Symptomatic or decrease in visual acuity interfering with ADL; operative intervention indicated	—	—
Ocular surface disease	Ocular surface disease	Asymptomatic or minimally symptomatic but not interfering with function	Symptomatic, interfering with function but not interfering with ADL, topical antibiotics or other topical intervention indicated		—	—
REMARK: Ocular surface disease indudes conjunctivitis, keratoconjunctivitis sicca, chemosis, keratinization, and palpebral conjunctival epithelial metaplasia.						
Optic disc edema	Optic disc edema	Asymptomatic	Decreased visual acuity (20/40 or better); visual field defect present	Decreased visual acuity (worse than 20/40); marked visual field defect but sparing the central 20 degrees	Blindness (20/200 or worse)	—
Vision-blurred vision	Blurred vision	Symptomatic not interfering with function	Symptomatic and interfering with function, but not interfering with ADL	Symptomatic and interfering with ADL	Disabling	—
Vitreous hemorrhage	Vitreous hemorrhage	Asymptomatic, clinical findings only	Symptomatic, interfering with function, but not interfering with ADL; intervention not indicated	Symptomatic, interfering with ADL; vitrectomy indicated	—	—
Watery eye (epiphora, tearing)	Watery eye	Symptomatic, intervention not indicated	Symptomatic, interfering with function but not interfering with ADL	Symptomatic, interfering with ADL	—	—
Ocular/Visual – Other (Specify)	Ocular – Other (Specify)	Symptomatic not interfering with function	Symptomatic and interfering with function, but not interfering with ADL	Symptomatic and interfering with ADL	Blindness (20/200 or worse)	Death
**PULMONARY/UPPER RESPIRATORY**						
Cough	Cough	Symptomatic, non-narcotic medication only indicated	Symptomatic and narcotic medication indicated	Symptomatic and significantly interfering with sleep or ADL	—	—
Dyspnea (shortness of breath)	Dyspnea	Dyspnea on exertion, but can walk 1 flight of stairs without stopping	Dyspnea on exertion but unable to walk 1 flight of stairs or 1 city block (0.1 km) without stopping	Dyspnea with ADL	Dyspnea at rest; intubation/ ventilator indicated	Death
Pleural effusion (non-malignant)	Pleural effusion	Asymptomatic	Symptomatic, intervention such as diuretics or up to 2 therapeutic thoracenteses indicated	Symptomatic and supplemental oxygen, >2 therapeutic thoracenteses, tube drainage, or pleurodesis indicated	Life-threatening (e.g., causing hemodynamic instability or ventilatory support indicated)	Death
Pneumonitis/ pulmonary infiltrates	Pneumonitis	Asymptomatic, radiographic findings only	Symptomatic, not interfering with ADL	Symptomatic, interfering with ADL; 0_2 indicated	Life-threatening; ventilatory support indicated	Death

Adverse Event	Short Name	GRADE 1	2	3	4	5
Pulmonary fibrosis (radiographic changes)	Pulmonary fibrosis	Minimal radiographic findings (or patchy or bi-basilar changes) with estimated radiographic proportion of total lung volume that is fibrotic of <25%	Patchy or bi-basilar changes with estimated radiographic proportion of total lung volume that is fibrotic of 25 – <50%	Dense or widespread infiltrates /consolidation with estimated radiographic proportion of total lung volume that is fibrotic of 50 – <75%	Estimated radiographic proportion of total lung volume that is fibrotic is ≥75%; honeycombing	Death
REMARK: Fibrosis is usually a "late effect" seen >3 months after radiation or combined modality therapy (including surgery). It is thought to represent scar/fibrotic lung tissue. It may be dfficult to distinguish from pneumonitis that is generally seen within 3 months of radiation or combined modality therapy.						
Pulmonary/Upper Respiratory – Other (Specify)	Pulmonary – Other (Specify)	Mild	Moderate	Severe	Life-threatening; disabling	Death
**RENAL/GENITOURINARY**						
Renal failure	Renal failure	—	—	Chronic dialysis not indicated	Chronic dialysis or renal transplant indicated	Death
Urinary frequency/urgency	Urinary frequency	Increase in frequency or nocturia up to 2 x normal; enuresis	Increase >2 x normal but <hourly	≥1 x/hr; urgency; catheter indicated	—	—
Renal/ Genitourinary – Other (Specify,)	Renal – Other (Specify)	Mild	Moderate	Severe	Life-threatening; disabling	Death
**SEXUAL/REPRODUCTIVE FUNCTION**						
Breast volume/hypoplasia	Breast	Minimal asymmetry; minimal hypoplasia	Asymmetry exists, ≤1/3 of the breast volume; moderate hypoplasia	Asymmetry exists, >1/3 of the breast volume; severe hypoplasia	—	—
REMARK: Breast volume is referenced with both arms straight overhead.						
Erectile dysfunction	Erectile dysfunction	Decrease in erectile function (frequency/rigidity of erections) but erectile aids not indicated	Decrease in erectile function (frequency/rigidity of erections), erectile aids indicated	Decrease in erectile function (frequency/rigidity of erections) but erectile aids not helpful; penile prosthesis indicated	—	—
Ejaculatory dysfunction	Ejaculatory dysfunction	Diminished ejaculation	Anejaculation or retrograde ejaculation	—	—	—
Endometrial hyperplasia	Endometrial hyperplasia	Mild	Moderate	Severe	Life-threatening; disabling	Death
Gynecomastia	Gynecomastia	—	Asymptomatic breast enlargement	Symptomatic breast enlargement; intervention indicated	—	—
Infertility/sterility	Infertility/sterility	—	Male: oligospermial/low sperm count Female: diminished fertility/ ovulation	Male: sterile/azoospermia Female: infertile/ anovulatory	—	—
Irregular menses (change from baseline)	Irregular menses	1 – 3 months without menses	>3 – 6 months without menses but continuing menstrual cycles	Persistent amenorrhea for >6 months	—	—
Libido	Libido	Decrease in interest but not affecting relationship; intervention not indicated	Decrease in interest and adversely affecting relationship; intervention indicated	—	—	—
Orgasmic dysfunction	Orgasmic function	Transient decrease	Decrease in orgasmic response requiring intervention	Complete inability of orgasmic response; not responding to intervention	—	—
Sexual/Reproductive Function – Other (Specify,)	Sexual – Other (Specify)	Mild	Moderate	Severe	Disabling	Death
Vaginal discharge (non-infectious)	Vaginal discharge	Mild	Moderate to heavy; pad use indicated	—	—	—

Préciser la méthode de réajustement du calcul.

		**GRADE**				
**[illegible]dverse Event**	**Short Name**	**1**	**2**	**3**	**4**	**5**
**VASCULAR**						
Phlebitis (including superficial thrombosis)	Phlebitis	—	Present			Death
Thrombosis/ thrombus/ embolism	Thrombosis/ thrombus/ embolism	—	Deep vein thrombosis or cardiac thrombosis; intervention (e.g., anticoagulation, lysis, filter, invasive procedure) not indicated	Deep vein thrombosis or cardiac thrombosis; intervention (e.g., anticoagulation, lysis, filter, invasive procedure) indicated	Embolic event including Pulmonary embolism or life threatening thrombus	Death
Vascular – Other (Specify,)	Vascular – Other (Specify)	Mild	Moderate	Severe	Life-threatening; disabling	Death
